# An antibody–drug conjugate active against antibiotic-resistant **Neisseria* gonorrhoeae*

**DOI:** 10.1073/pnas.2534217123

**Published:** 2026-07-28

**Authors:** Hayley Lavender, Vincent Oliver, Daniel Lucy, Timothy A. Barendt, Ann E. Jerse, Christoph M. Tang

**Affiliations:** ^a^ Sir William Dunn School of Pathology, University of Oxford, Oxford OX1 3RE, United Kingdom; ^b^ The Department of Clinical Infection, Microbiology, and Immunology, Institute of Infection, Veterinary and Ecological Sciences, University of Liverpool, Liverpool L69 7BE, United Kingdom; ^c^ Henry M. Jackson Foundation for the Advancement of Military Medicine, Bethesda, MD 20817; ^d^ Department of Chemistry, University of Oxford, Oxford OX1 3TA, United Kingdom; ^e^ School of Chemistry, University of Birmingham, Birmingham B15 2TT, United Kingdom; ^f^ Department of Microbiology and Immunology, Uniformed Services, University of the Health Sciences, Bethesda, MD 20814

**Keywords:** *Neisseria gonorrhoeae*, antibody–drug conjugate, IgA protease, antimicrobial peptide, antimicrobial resistance

## Abstract

**Neisseria* gonorrhoeae* is the causative agent of gonorrhea, a sexually transmitted infection which is rising in incidence, with increasing drug-resistant strains posing a significant public health threat. To address the urgent need for novel therapies, we developed an antibody–drug conjugate (ADC) that targets this important human pathogen. We utilized Tridecaptin A_1,_ a potent antimicrobial peptide (AMP) against Gram-negative bacteria that exhibits significant toxicity against human cells, limiting its development for clinical use. By conjugating the Tridecaptin A_1_ analogue, Oct-TriA_1_ to a monoclonal antibody (mAb) that specifically targets gonococcal MtrE, the outer membrane component of a drug efflux pump that is upregulated in resistant strains, we aim to selectively deliver the AMP to the gonococcus. However, Oct-TriA_1_ was not bactericidal when directly conjugated to mAb. To circumvent this, we exploited an immune evasion mechanism employed by the gonococcus by introducing a linker between Oct-TriA_1_ and the mAb which is specifically cleaved by the IgA protease (IgAP) secreted by the gonococcus; the IgAP inactivates human IgA. This ADC has no detectable toxicity for relevant human cells, kills the gonococcus in an MtrE- and IgAP-dependent manner, and is active against a strain which is resistant to first line agents. This modular ADC platform could be extended to other bacterial pathogens which employ proteases to evade immune killing, offering a strategy in the fight against antimicrobial resistance.

## Significance

The global spread multidrug-resistant **Neisseria* gonorrhoeae* threatens to undermine antibiotic treatment. Treatment failures of last-line antibiotics are increasingly reported, highlighting an urgent need to develop novel, innovative treatments. We developed a modular antibody–drug conjugate (ADC) that exploits two conserved gonococcal virulence factors, the efflux pump channel, MtrE, and the IgA protease, to achieve specific delivery of a potent antimicrobial peptide (AMP). Our approach overcomes associated AMP host-cell toxicity, which has historically limited their therapeutic potential. By demonstrating that a bacterial protease can activate an ADC through release of an AMP payload at the pathogen surface, our findings can be expanded to generate precision-targeted antimicrobials against other multidrug-resistant bacterial pathogens.

**Neisseria* gonorrhoeae* is an obligate human pathogen, which causes the sexually transmitted infection (STI), gonorrhea (1). There are estimated to be over 82 million cases of gonococcal infection worldwide every year with the highest incidence found in low- and middle-income countries (2). Complications of gonococcal infection disproportionally affect women, and include pelvic inflammatory disease, ectopic pregnancy, and infertility, as well as increased transmission and acquisition of HIV (3–5). Due to its exquisite adaptation to survival in its host, *N. gonorrhoeae* has evolved sophisticated mechanisms to subvert human immune defense (6–8), and secretes an IgA protease (IgAP) which specifically cleaves the hinge region of human IgA1 (hIgA1) (9).

Due to the rapid emergence of antimicrobial resistance (AMR), the World Health Organization has declared *N. gonorrhoeae* as a priority pathogen for the development of novel interventions (10), while the Centers for Disease Control classified drug-resistant gonorrhea as an urgent threat (11). Of particular concern are multidrug resistant (MDR) strains which are resistant against the first line agents, extended spectrum cephalosporins (ESCs) and azithromycin. Expression of multidrug efflux pumps is an important mechanism conferring AMR in *N. gonorrhoeae*. The MtrCDE tripartite pump belongs to the resistance-nodulation-cell division family (12, 13), and can export multiple antibiotics from the gonococcus. Trimeric MtrE forms an outer membrane export channel with a periplasmic α-barrel (14, 15), which is linked by MtrC to MtrD at the inner membrane. MtrE also acts as the outer membrane channel for other export pumps including the FarAB and MacAB systems (16, 17). Resistance against several antibiotics is enhanced by increased expression of MtrE, either by loss-of-function mutations of *mtrR*, which encodes the repressor of the *mtrCDE* operon or *mtrCDE* promoter mutations (18–23). Therefore, MtrE is an attractive surface-expressed target for therapeutic interventions as it is overexpressed by many drug-resistant isolates (14, 24).

Current antibiotic development for *N. gonorrhoeae* includes zoliflodacin, which inhibits DNA gyrase B, and gepotidacin, which targets two DNA topoisomerases, which both show noninferiority to standard therapies in Phase III trials (25–27). However, while these agents have shown promise, the development of resistance remains a concern and alternative agents including antimicrobial peptides (AMPs) offer another strategy to combat AMR (28, 29). Tridecaptins are acylated tridecapeptide AMPs produced by *Paenibacillus* and *Bacillus* spp. (30) Tridecaptin A_1_ (TriA_1_) is highly active against Gram-negative bacteria although its activity against *N. gonorrhoeae* is unknown (31). Tridecaptins disrupt cell membranes with its acyl chain critical for activity. However, due to its amphiphilic nature, TriA_1_ interacts nonspecifically with cell membranes through electrostatic interactions (32–34), leading to significant host cell cytotoxicity which has limited the clinical development of tridecaptins and other AMPs with membrane-disrupting properties (34).

Here we describe the development of an anti-*N. gonorrhoeae* antibody–drug conjugate (ADC) consisting of a monoclonal antibody (mAb) targeting MtrE which is covalently coupled to the highly active TriA_1_ analogue, Oct-TriA_1_. ADCs have been developed against *Staphylococcus aureus* and *Pseudomonas aeruginosa* in which release of the drug from the antibody is mediated by host intracellular proteases, such as cathepsins (35, 36). These enzymes are only active in certain cellular compartments, limiting the distribution of activity of these ADCs. Instead, we exploited the specific activity of a gonococcal enzyme, the IgAP, to release Oct-TriA_1_ from the mAb targeting MtrE. Thus, the delivery of Oct-TriA_1_ to its intended target, the gonococcal outer membrane, is promoted initially by the ADC binding to MtrE, then by the local release of the AMP mediated by bacterial IgAP. We demonstrate that our ADC, which has an IgAP-cleavable linker between the mAb and tridecaptin, displays MtrE- and IgAP-dependent antimicrobial activity against the gonococcus, and kills an MDR *N. gonorrhoeae* isolate while having no demonstrable host cell toxicity. These modular ADCs offer a flexible and customizable approach to deliver a range of antimicrobial compounds to other AMR bacterial pathogens that secrete proteases that cleave defined linear sequences.

## Results

### Oct-TriA_1_ has Potent Antimicrobial Activity Against *N. gonorrhoeae*.

The tridecaptin analogue, Oct-TriA_1_ (Table 1) has potent bactericidal activity against a range of Gram-negative bacteria including *Klebsiella pneumoniae* and **Acinetobacter* baumannii* (33). To establish whether Oct-Tri_1_ is also active against *N. gonorrhoeae*, we determined its minimum inhibitory concentration (MIC) against eight *N. gonorrhoeae* WHO reference strains with known antibiotic susceptibility profiles (37, 38), along with the well-characterized isolates, FA1090 and MS11 (37, 39–41). Oct-Tri_1_ displays marked anti-gonococcal activity with MICs in the sub-μg mL^−1^ range for all isolates (0.2 to 0.78 μg mL^−1^); Oct-Tri_1_ is also active (MIC, 0.78 μg mL^−1^) against G97687, a MDR isolate which is resistant to ESCs and azithromycin (42) (Table 2). Additionally, the MICs for FA1090Δ*mtrE* (0.1 and 0.2 μg mL^−1^ for the *mtrE* mutant and wild-type, respectively) and MS11Δ*mtrE* (0.2 and 0.78 μg mL^−1^) were lower than the corresponding wild-type strains, indicating that Oct-TriA_1_ is a substrate of MtrE-dependent efflux pumps.

**Table 1.** Oct-TriA_1_ analogue sequences used in this study

Peptide name	Peptide	Peptide sequence	Molecular weight (Da)
Oct-TriA_1_	Oct-TriA_1_	Octanoyl-DVal-DDab-Gly-DSer-DTrp-Ser-Dab-DDab-Phe-Glu-Val-Dalle-Ala-OH	1,520.8
d-Oct-TriA_1_	Oct-TriA_1_-(C)	Octanoyl-DVal-DDab-Gly-DSer-DTrp-Ser-Dab-DDab-Phe-Glu-Val-Dalle-Ala-O2Oc-O2Oc-Cys-NH2	1,934.8
Oct-TriA_1_-c	Oct-TriA_1_-(LPRPP)	Octanoyl-DVal-DDab-Gly-DSer-DTrp-Ser-Dab-DDab-Phe-Glu-Val-Dalle-Ala-O2Oc-Leu-Pro-Arg-Pro-Pro-OH	2,226.8
cl-Oct-TriA_1_	Oct-TriA_1_-(LPRPPAPVFS-C)	Octanoyl-DVal-DDab-Gly-DSer-DTrp-Ser-Dab-DDab-Phe-Glu-Val-Dalle-Ala-O2Oc-Leu-Pro-Arg-Pro-Pro-Ala-Pro-Val-Phe-Ser-O2Oc-Cys-NH2	2,974.8
uncl-Oct-TriA_1_	Oct-TriA_1_-(VKPAPSPAAN-C)	Octanoyl-DVal-DDab-Gly-DSer-DTrp-Ser-Dab-DDab-Phe-Glu-Val-Dalle-Ala-O2Oc-Val-Lys-Pro-Ala-Pro-Ser-Pro-Ala-Ala-Asn-O2Oc-Cys-NH2	2,845.7

**Table 2.** Comparison of the antimicrobial activity of Oct-TriA_1_ with different *N. gonorrhoea* isolates^*^

	**Neisseria* gonorrhoea* strain														
Peptide	WHO F	WHO G	WHO K	WHO L	WHO M	WHO N	WHO O	WHO P	MS11	MS11Δ*mtrE*	MS11Δ*IgAP*	FA1090	FA1090Δ*mtrE*	FA1090Δ*IgAP*	G97687
Oct-TriA_1_	0.2	0.39	0.78	0.2	0.39	0.39	0.39	0.39	0.78	0.2	0.39	0.2	0.1	0.1	0.2
Oct-TriA_1_-*c*	1.56	3.13	3.13	1.56	1.56	3.13	3.13	3.13	3.13	1.56	6.25	0.78	0.78	0.78	1.56

^*^Activities are reported in μg mL^−1^.

### An ADC with Oct-TriA_1_ Directly Conjugated to an α-MtrE mAb Lacks Activity Against *N. gonorrhoeae*.

**Fig. 1.**
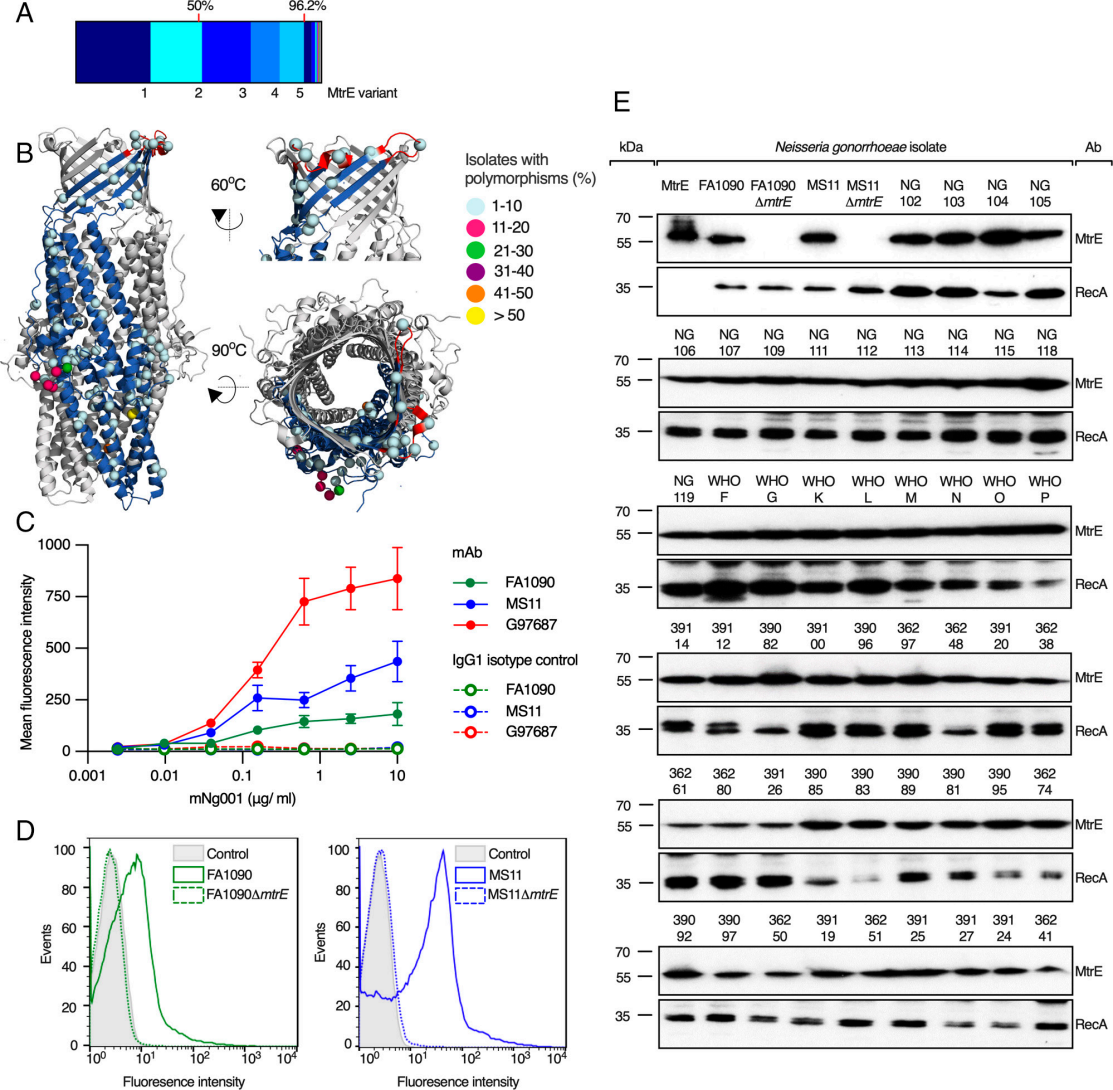
mAb mNg001 recognizes gonococcal MtrE. (*A*) Distribution of MtrE amino acid sequences in *N. gonorrhoeae* isolates on PubMLST (n = 18,635). Percentage of isolates harboring MtrE variants represented as Parts of a Whole. Red lines indicate 50% and 96.2% of *N. gonorrhoeae* isolates. (*B*) Amino acids colored according to the proportion of isolates with polymorphisms compared to the most common MtrE variant (i.e., encoded by allele 1), and mapped onto trimeric MtrE (PDB accession number 4MT0); Outer membrane loops (red) (*C*) Dose-dependent binding of mAb mNg001 (filled circles), or isotype control antibody (unfilled circles), to *N. gonorrhoeae* strains FA1090 (green circles), MS11 (blue circles), and G97687 (red circles). Mean fluorescence intensity ± SD determined by flow cytometry (n = 3). (*D*) mNg001 binds to wild-type strains; FA1090 and MS11 (solid lines) but not the isogenic Δ*mtrE* mutants (dashed lines). (*E*) Western blot analysis of mNg001 binding to MtrE in whole cell lysates of 52 *N. gonorrhoeae* strains selected from a variety of core genome groups. Recombinant MtrE and wild-type FA1090 and MS11 and Δ*mtrE* mutants used as mAb controls; RecA, loading control.

The use of tridecaptins for treating Gram-negative pathogens has been hampered by significant host cell toxicity (34). Therefore, we decided to conjugate an Oct-TriA_1_ analogue to a mAb that binds a gonococcal surface protein, thereby delivering the AMP to *N. gonorrhoeae*. We generated mAbs against MtrE, the surface component of the several gonococcal export pumps (43, 44), because MtrE is overexpressed by many drug-resistant *N. gonorrhoeae* isolates including G97687 (41, 42). We examined the conservation of MtrE in over 18,600 gonococcal isolates in PubMLST, compared to >5,000 isolates analyzed previously (45, 46), and identified 94 unique protein sequences. The five most prevalent MtrE sequences account for >96% of gonococcal isolates (Fig. 1*A* and *SI Appendix*, Table S1). The location of polymorphic residues in MtrE was mapped onto its structure (PDB:4MT0) (15) (Fig. 1*B*), and revealed that the two surface exposed loops of MtrE are highly conserved; a single sequence of loop 1 (GSLSGGNV) and loop 2 (SVELGGLFKSGTG) are found in 99.9% and 98.2% of strains, respectively (*SI Appendix*, Table S2); most polymorphisms are located in the periplasmic α- barrel of MtrE. Furthermore, gonococcal MtrE alleles were present in only six out of >45,000 **Neisseria* meningitidis* isolates and three of 61 **Neisseria* cinerea* isolates (*SI Appendix*, Tables S3 and S4). This makes MtrE a suitable candidate for targeting the gonococcus.

mAbs were generated against recombinant MtrE by standard hybridoma technology. We characterized one mAb, mNg001, in more detail. Flow cytometry demonstrated that mNg001 binds *N. gonorrhoeae* in a dose-dependent manner (Fig. 1*C*) and exhibited higher binding to G97687 and MS11 compared to FA1090, consistent with MtrE overexpression by these strains (13, 42). As expected, mNg001 binding was MtrE-specific with no binding detected to FA1090Δ*mtrE* or MS11Δ*mtrE* [*P* = 0.0003 and 0.0018, respectively, compared to wild-type bacteria (Fig. 1*D*)]. We also assessed the ability of mNg001 to recognize MtrE from 52 gonococcal strains belonging to different core genome groups (47) and included the five most prevalent MtrE alleles by western blot analysis (*SI Appendix*, Table S1); mNg001 bound to all gonococcal strains analyzed (Fig. 1*E*). In contrast, the mAb did not recognize any protein in seven *N. meningitidis* isolates or 16 isolates of other *Neisseria* spp. (*SI Appendix*, Fig. S1 and Table S5). Sequence analysis (*SI Appendix*, Fig. S2) shows that meningococcal and commensal *Neisseria* MtrE share 95.7 to 97.5% amino acid identity to the most common gonococcal MtrE variant. Six amino acids are consistently different in MtrE from *N. meningitidis* and commensals compared with the gonococcus (*SI Appendix*, Fig. S3). Five substitutions (Q39, S43, S46, R248, A334S) are located in the α-helical periplasmic domain and β- barrel of MtrE whereas there is only one substitution, V320A, within the outer membrane loop 2 which may explain the species specificity of mAb mNg001. Taken together, mNg001 binds an epitope that appears to be highly conserved in MtrE from *N. gonorrhoeae* but not other *Neisseria* spp., making it suitable for specific delivery of AMPs.

**Fig. 2.**
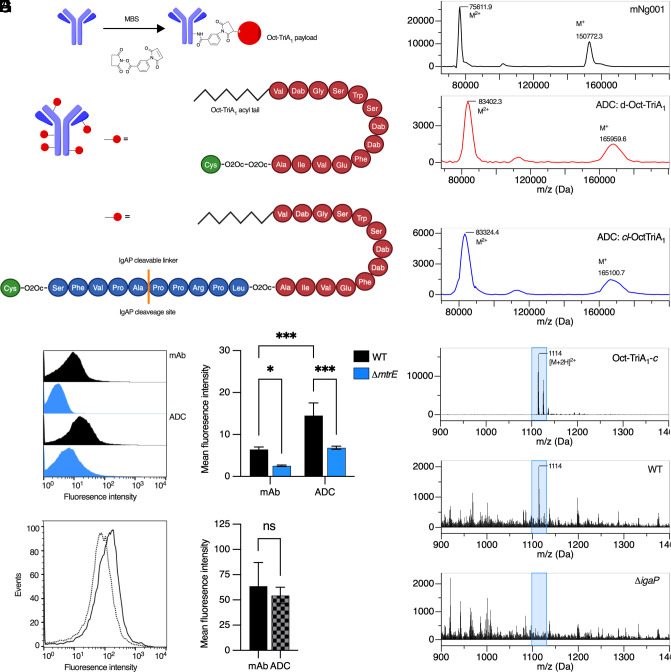
Construction and characterization of ADC with an IgAP cleavable linker. (*A*) Strategy for conjugation of mNg001 (purple) to OctTriA_1_ analogues (Red circle) using an MBS amine-to-sulfhydryl crosslinker containing N- hydroxysuccinimide (NHS) ester and maleimide groups. (*B*) Schematic of directly conjugated ADC (not drawn to scale) consisting of mNg001 (purple) and OctTriA_1_ analogue (red) synthesized with two O2Oc spacers (8-amino-3,6-dioxaoctanoic acid) and a C-terminal cysteine (green), acyl tail indicated (black). Dab = diaminobutyric acid (*C*) MALDI-TOF MS analysis of mNg001 (*Top*, black) and directly conjugated ADC covalently linked to the d-OctTriA_1_ analogue (*Bottom*, red). Change in mass, compared to mNg001 indicates a DAR of approximately 8 peptides per ADC. (*D*) Schematic of *cl*-OctTriA_1_ peptide analogue incorporating an IgAP cleavable linker (LPRPPAPVFS) between two 8-amino-3,6-dioxaoctanoic acid spacers with a C-terminal cysteine for conjugation. (*E*) MALDI-TOF MS of ADC-*cl*-Oct-TriA_1_ indicates a DAR of approximately 5 peptides per antibody compared to mNg001. Binding of mNg001 (mAb) and ADC conjugated to *cl*-OctTriA_1_ (ADC) to gonococcal strains (*F*) FA1090 wild-type (black) and isogenic Δ*mtrE* (blue) or (*G*) G97687 by flow cytometry. Data (*F* and *G*) shown as mean ± SD (n = 3). two-way ANOVA; ***P < 0.001, *P ≤ 0.05 and ns, *P* > 0.05. (*H*) Electrospray ionization (ESI^+^) mass spectrometry analysis of Oct-TriA_1_-*c* (blue box) in PBS as a mass spectrometry control (*Top*) found a species of 1114 [M + 2H]^2+^, [C_105_H_170_N_26_O_27_]^2+^. Species equivalent to OctTriA_1_-*c* (blue box) were only visible after ADC-*cl*-Oct-TriA_1_ was incubated in supernatant from wild-type FA1090 (*Middle*) but not FA1090Δ*igaP* (*Bottom*).

Next, we examined sites in Oct-TriA_1_ that would allow conjugation to mNg001. Oct-TriA_1_ depends on its N-acyl chain and several amino acids, while the C-terminal alanine is dispensable (32). Therefore, we reasoned that this tridecaptin might retain its activity when directly conjugated directly to mAb mNg001 *via* its C terminus. We covalently conjugated mNg001 to Oct-TriA_1_ (d-Oct-TriA_1_) with two 8-amino-3,6-dioxaoctanoic acid [O2Oc] spacer groups and a cysteine at the C terminus; *m*-maleimidobenzoyl-*N*-hydroxysuccinimide (MBS) was used as the crosslinker to generate amine-sulfhydryl linkages (48) (Fig. 2 *A* and *B*). This ADC was analyzed by matrix-assisted laser desorption/ionization time-of-flight mass spectrometry (MALDI-TOF MS), which demonstrated that the average drug-to-antibody ratio (DAR) was approximately 8 (Fig. 2*C*). However, the ADC lacked any detectable bactericidal activity against *N. gonorrhoeae* even at concentrations of >512 µg mL^−1^ (equivalent of 100 µg mL^−1^ of Oct-TriA_1_), indicating that Oct-TriA_1_ is inactive when bound to a mAb.

## Introduction of an IgAP Cleavable Linker Into an ADC Allows Tridecaptin Release

Therefore, we decided to exploit the activity of the IgAP secreted by gonococci to release Oct-TriA_1_ from the α-MtrE mAb. Of note, noninvasive *Neisseria* spp., in contrast to *N. gonorrhoeae* and *N. meningitidis*, do not secrete an IgAP (9) adding another layer of specificity to the ADC (*SI Appendix*, Table S4 and Table S6). The gonococcal IgAP recognizes and cleaves linear 10 amino acid peptides (49). Therefore, we synthesized Oct-TriA_1_ with an IgAP-cleavable linker (LPRPP/APVFS, /indicates the cleavage site) flanked by O2Oc groups to the C terminus of Oct-TriA_1_; a C-terminal cysteine was added after the linker (generating a cleavable Oct-TriA_1_, cl-Oct-TriA_1_) to allow conjugation to the mAb (Table 1 and Fig. 2*D*).

We examined whether cl-Oct-TriA_1_ can be cleaved by the gonococcal IgAP. cl-Oct-TriA_1_ was incubated with culture supernatants from wild-type FA1090 or an isogenic Δ*igaP* mutant. Electrospray ionization mass spectrometry (ESI-MS) analysis of the uncleaved peptide showed species with a mass-to-charge ratio (m/z) of 1488, corresponding to *cl*-Oct-TriA_1_ (*SI Appendix*, Figs. S4 *A* and S5*A*]. After incubation with supernatants from wild-type bacteria but not from the Δ*igAP* mutant, a species with a m/z of 1114 (*SI Appendix*, Figs. S4*B* and S5*B*] was detected from cl-Oct-TriA_1_, consistent with the mass of cleaved Oct-TriA_1_ (i.e., Oct-TriA_1_ with -O2Oc-LPRPP). To determine whether cleaved cl-Oct-TriA_1_ retained its activity, we synthesized a peptide corresponding to cl-Oct-TriA_1_ after cleavage by the IgAP (Oct-TriA_1_-*c*) (Table 1) and found that it is highly active against *N. gonorrhoeae* [MICs, 0.78 to 6.25 μg mL^−1^, (Table 2)].

As gonococcal IgAP can cleave cl-Oct-TriA_1_ and the residual AMP retains bactericidal activity, we next determined if the gonococcal IgAP could cleave cl-Oct-TriA_1_ conjugated to mAb mNg001. Again, we used MBS to covalently link cl-Oct-TriA_1_ to the mAb (Fig. 2*D*). The ADC was subjected to MALDI-TOF MS which demonstrated a DAR of ~5 (Fig. 2*E*). We also generated ADCs conjugated to an identical Oct-TriA_1_ analogue but with an uncleavable 10 amino acid linker (uncl-Oct-TriA_1_, with VKPAPSPAAN) (Table 1) uncl-Oct-TriA_1_, which also yielded a DAR of ~6 (*SI Appendix*, Fig. S6). To assess if the ADCs still recognize *N. gonorrhoeae* after conjugation, we analyzed the binding to FA1090 of mAb mNg001 or the ADCs. Flow cytometry demonstrated that binding of the ADCs was dependent on MtrE expression (Fig. 2*F*) with significantly decreased binding to FA1090Δ*mtrE* compared to FA1090 (*P* = 0.0003). The ADC displayed marginally more binding to FA1090 and FA1090Δ*mtrE* than the unconjugated mAb (*P* = 0.0002 and 0.0093 respectively), (Fig. 2*F*) which may indicate low level, nonspecific electrostatic interactions with the AMP (32–34). Furthermore, we compared binding of mNg001 and the ADCs to the multidrug resistant isolate G97687, and no significant difference in binding was observed to this strain (*P* = 0.5586, Fig. 2*G*).

To examine if the IgAP can release Oct-TriA_1_ from the mAb, we incubated the ADCs with supernatants from wild-type FA1090 or the corresponding Δ*igAP* mutant. ESI-MS confirmed that the presence of IgAP liberates Oct-TriA_1_ from the ADC generated with cl-Oct-TriA_1_ (a species with m/z of 1114} corresponding to free, cleaved cl-Oct-TriA_1_, Oct-TriA_1_-*c*, Fig. 2*H* and *SI Appendix*, Fig. S5*B*) but not the ADC generated with uncl-Oct-TriA_1_ (i.e., with the noncleavable linker), demonstrating that the gonococcal IgAP specifically liberates Oct-TriA_1_ from ADCs containing the cleavable linker (*SI Appendix*, Fig. S7).

### ADCs Exhibit Reduced Host Cytotoxicity.

**Fig. 3.**
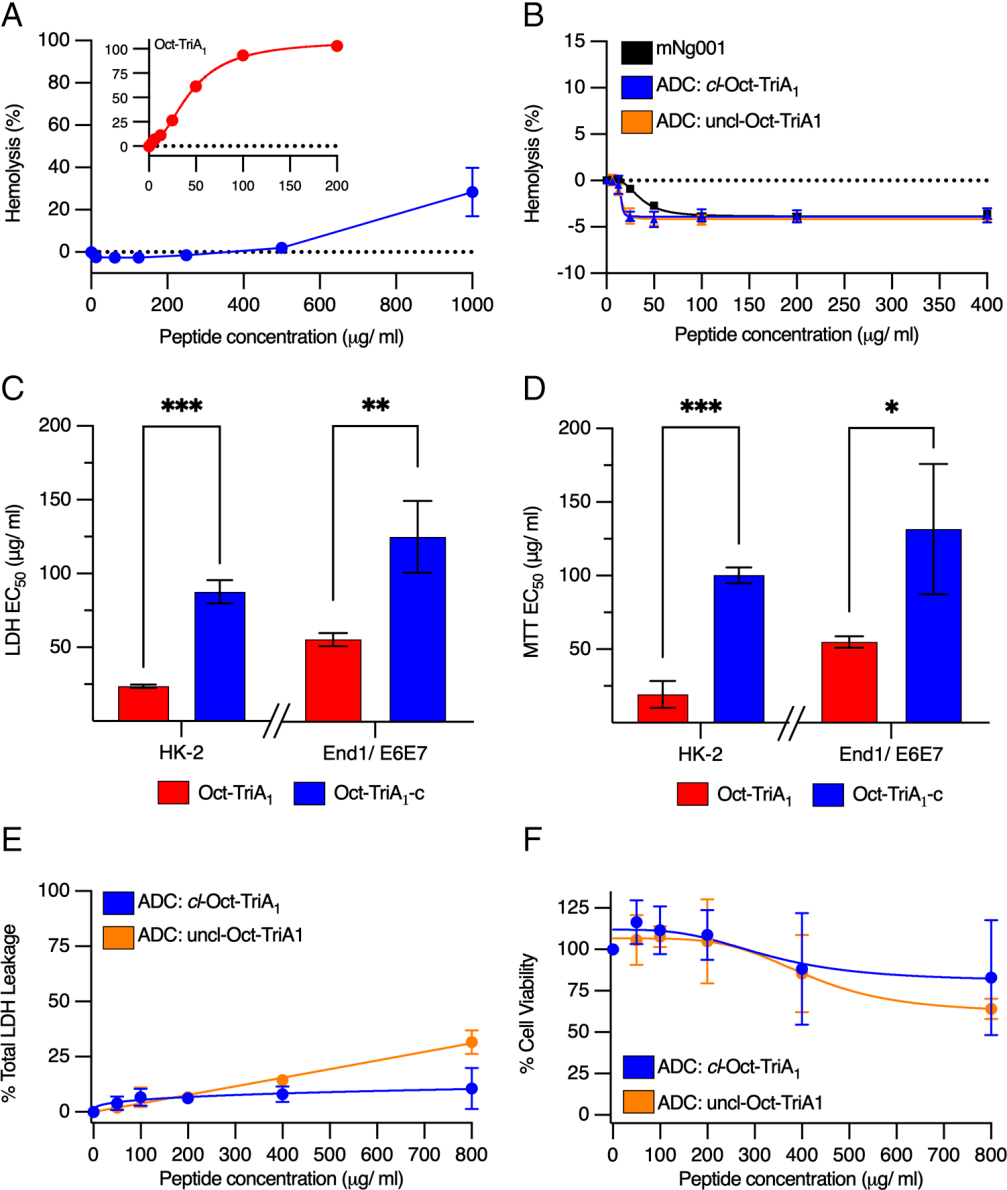
Conjugation of OctTriA_1_ to ADC reduces host cell cytotoxicity. Hemolysis of human erythrocytes by OctTriA_1_ derivatives. (*A*) OctTriA1, (red, *Inset*), OctTriA_1_-*c* (blue) and (*B*) mNg001 (black) with ADCs conjugated to *cl*-OctTriA_1_ (blue) and un*cl*-OctTriA_1_ (orange). Data are the mean ± SD (n = 3). Cytotoxicity of OctTriA_1_ peptides against HK-2 renal proximal tubular and End1 E6/E7 endocervical cells measured by (*C*) LDH release and (*D*) reduction of MTT; OctTriA_1_, (red) and OctTriA_1_-*c* (blue). Cytotoxicity of ADCs conjugated to *cl*-OctTriA_1_ (blue) and un*cl*-OctTriA_1_ (orange) toward HK-2 cells assayed by (*E*) LDH release and (*F*) MTT reduction. Data are the mean ± SD (n = 3). Unpaired *t* test; ***P < 0.001, **P < 0.01, and *P ≤ 0.05.

Tridecaptins cause significant host cell cytotoxicity (32). Therefore, we evaluated the hemolytic activity (HC_50_) of Oct-TriA_1_ derivatives against human erythrocytes (Fig. 3*A* and Table 3). Unmodified Oct-TriA_1_ had a HC_50_ of 39.4 μg mL^−1^, while Oct-TriA_1_-*c* (the peptide generated by IgAP cleavage of the ADC) demonstrated > 25-fold reduced hemolytic activity (HC_50_ of >1 mg mL^−1^). Of note, the HC_50_ of the ADCs was >2.8 mg mL^−1^, equivalent to >400 μg mL^−1^ of Oct-TriA_1_-*c* with a DAR of 8 (Fig. 3*B*), demonstrating that conjugation of Oct-TriA_1_ to a mAb virtually abolishes its toxicity.

**Table 3.** Comparison of human erythrocyte hemolysis (HC_50_) and cytotoxicity (EC_50_) against HK-2 cells on exposure to Oct-TriA_1_ analogues and ADCs^*^

Peptide name	Hemolysis (HC_50_)^†^	LDH (EC_50_^‡^)			MTT (EC_50_^‡^)		
		4 h	24 h	48 h	4 h	24 h	48 h
Oct-TriA_1_	39.4	23.19	14.14	<0.1	21.15	7.93	8.07
Oct-TriA_1_-c	>1,000	86.42	57.71	50.03	101.98	78.78	61.09
cl-Oct-TriA_1_	>200	>400	–	–	315.43	–	–
uncl-Oct-TriA_1_	>200	>400	–	–	>400	–	–

^*^Activities are reported in μg mL^−1^.

^†^The concentration of peptide or ADC that lyses 50% of erythrocytes.

^‡^Half maximal effective concentration.

Additionally, we assessed the toxicity of Oct-TriA_1_ derivatives against HK-2 human proximal renal tubule cells (50), and upper cervical End1/E6E7 cells (51) using LDH and MTT assays. These cell lines were chosen as AMPs can cause nephrotoxicity while the gonococcus infects cells in the upper vagina and cervix. The half-maximal inhibitory concentration (IC_50_) for Oct-TriA_1_-*c* was significantly higher than Oct-TriA_1_ for both the LDH release (*P* = 0.0002 and 0.0081) and cell viability (*P* = 0.0002 and 0.0406) with HK-2 and End1/E6E7 cells, respectively (Fig. 3 *C* and *D* and Table 3); End1/E6E7 cells have increased tolerance for Oct-TriA_1_ analogues, demonstrated by the higher IC_50_ for Oct-TriA_1_-*c* than HK-2 cells (Table 3). Remarkably, the ADCs had an IC_50_ > 5.6 mg mL^−1^, equivalent to >800 μg mL^−1^ of Oct-TriA_1_ (Fig. 3 *E* and *F* and Table 3). Taken together, modification of the C-terminus of Oct-TriA_1_ significantly reduces host-cell toxicity, while the ADCs displayed no detectable toxicity against cells from the renal and genitourinary tracts.

### ADCs have MtrE- and IgAP-Dependent Anti-Gonococcal Activity and Are Active Against an MDR Strain.

**Fig. 4.**
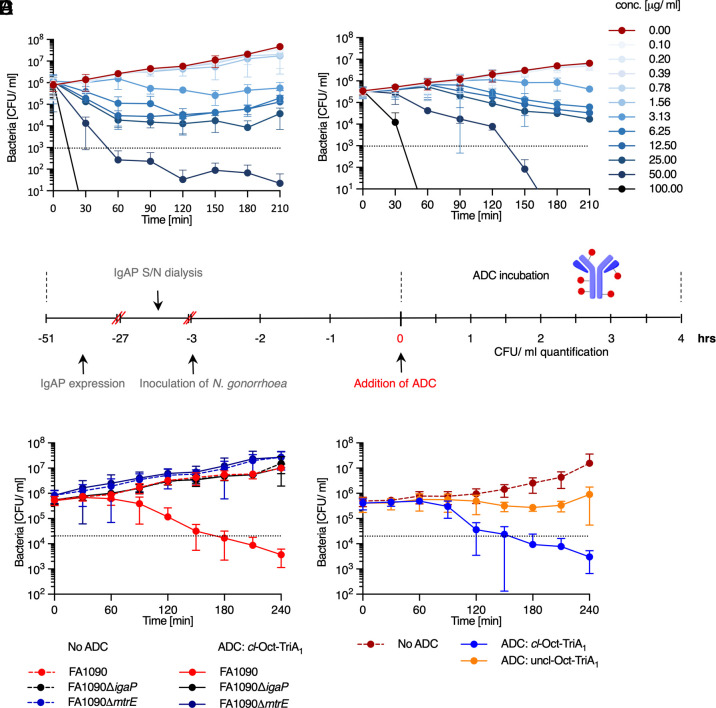
ADCs are bactericidal in the presence of the gonococcal IgAP. Dose-dependent time-to-kill analysis of OctTriA_1_-c against (*A*) FA1090 and (*B*) G97687. No peptide is shown in red with increasing concentrations of peptide from 0.1 μg mL^−1^ (light blue) to μg mL^−1^ (dark blue). (*C*) Time-to-kill curve analysis with ADCs protocol. *N. gonorrhoeae* were grown for 24 h prior to harvesting supernatant with or without IgAP, wild-type, and Δ*igAP,* respectively. Supernatant was dialyzed into fresh media prior to inoculating with respective *N. gonorrhoeae* strains. After 3 h growth, ADCs or peptides were added, and CFU/mL was calculated every 30 min for 4 h. (*D*) Time-to-kill curve analysis of ADC-*cl*-OctTriA_1_ against FA1090 (red), isogenic *ΔIgAP* (black), and *ΔMtrE* (blue). No ADC (dashed line) and ADC (line). (*E*) Time-to-kill curve analysis of ADC-*cl*-OctTriA_1_ (blue), ADC-un*cl*-OctTriA_1_ (orange), and no mAb control (red, dashed line) against the MDR gonococcal strain, G97687. All data shown are the mean ± SD of CFU/ml (n = 3).

Critically, we next assessed the bactericidal activity of the ADCs against *N. gonorrhoeae*. As bacteria need to secrete IgAP to release the AMP from ADCs, the activity of ADCs was assessed in time-to-kill assays (Fig. 4). Oct-TriA_1_-*c* exhibited dose-dependent bactericidal activity against FA1090 and the MDR strain G97687 in time-to-kill assays, with undetectable survival of FA1090 in 50 μg mL^−1^ of the AMP after 60 min; there was no survival of G97687 after 180 min (Fig. 4 *A* and *B*); G97687 has a higher MIC than FA1090 for Oct-TriA_1_-*c* (Table 2).

As each ADC has an average DAR of 5 to 8 we used a concentration equivalent to approximately 100 μg mL^−1^ of Oct-TriA_1_ in time-to-kill assays; initial bacterial growth allowed expression of the IgAP prior to treatment with ADC’s (Fig. 4*C*) as preliminary data indicated maximal IgA cleavage after 8 h (*SI Appendix*, Fig. S8*A*]. The survival of FA1090 decreased after 90 min incubation with the ADC containing Oct-TriA_1_ conjugated *via* the cleavable linker and was below the limit of detection (LOD) after 180 min (Fig. 4*D*). Survival of FA1090 without the initial growth in supernatants containing IgAP reduced to below the limit of detection after 240 min (*SI Appendix*, Fig. S8 *B* and *C*). In contrast, the ADC with Oct-TriA_1_ conjugated *via* a noncleavable linker had no bactericidal activity against FA1090 (*SI Appendix*, Fig. S9). The ADC with the cleavable linker displayed no bactericidal activity in the absence of MtrE, the mAb target (i.e., against FA1090Δ*mtrE*), or the IgAP (i.e., against FA1090Δ*igaP*). Importantly, this ADC efficiently killed the MDR isolate G97687 (Fig. 4*E*).

### ADCs Promote Clearance of *N. gonorrhoeae* in a Lower Genital Tract Infection Model.

**Fig. 5.**
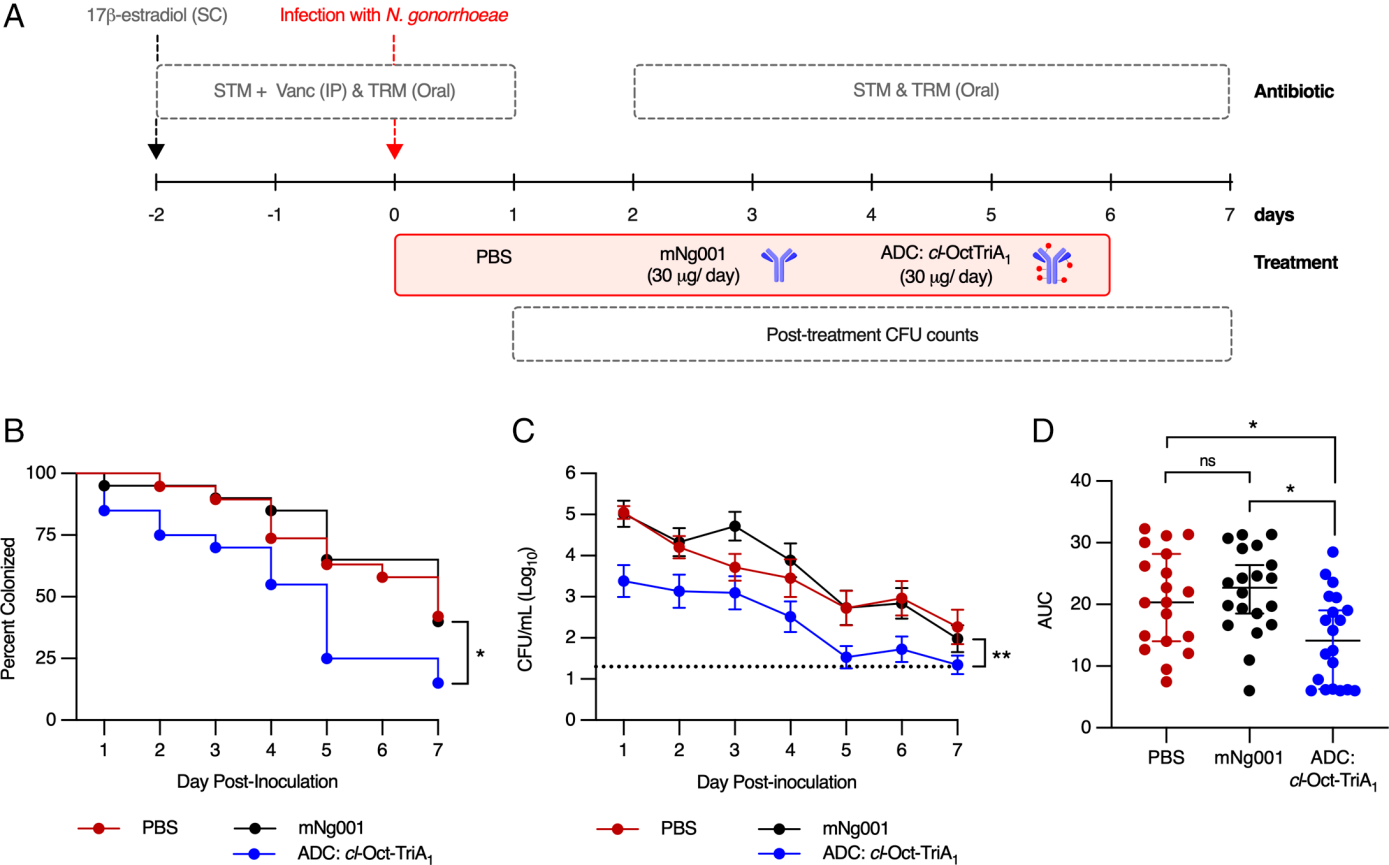
ADC- *cl*-OctTriA_1_ enhances gonococcal clearance in the lower genital tract infection model. (*A*) Timeline of murine study to assess the efficacy of the ADC-*cl*-OctTriA_1_, the unconjugated mAb, mNg001, and PBS. (*B*–*D*) Mice were treated intravaginally with mNg001 (black) or ADC-*cl*-OctTriA_1_ (blue) 2 h prior to inoculation with *N. gonorrhoeae* FA1090 on Day 0 with 10^6^ CFU; the control group received PBS (red) and subsequently treatments were administered 1 h after sampling. (*B*) Percentage of mice in each group colonized with *N. gonorrhoeae* postinfection and treatment shown using Kaplan–Meier curves. Significance established using the Mantel-Cox log-rank test. (*C*) Bacterial burden (Log_10_ CFU l^−1^) in vaginal washes on days postinfection: groups were compared using 2-way ANOVA with Bonferroni’s multiple comparisons test. (*D*) Mean ± SEM AUC of bacterial recovery after infection; n = 19 for the PBS control. and 20 for test groups mNg001 and ADC:*cl*-OctTriA_1_. Horizontal bars indicate the median with 95% CI AUC for each group and were compared using the Kruskal–Wallis test. Significance cut-offs, **P < 0.01, *P ≤ 0.05, and ns > 0.05.

Having established the MtrE- and IgAP-dependent bactericidal activity of our ADC in vitro, we next evaluated its therapeutic efficacy in the lower genital tract murine model of infection (52). Mice were treated with slow-release estradiol pellets to promote gonococcal colonization and antibiotics to reduce the vaginal commensal flora prior to inoculation with *N. gonorrhoeae* FA1090 (Fig. 5*A*); gonococcal colonization was assayed daily following bacterial challenge (52). Mice treated with ADC exhibited significantly faster clearance of FA1090 compared to mNg001 alone (*P* = 0.0113) or PBS (*P* = 0.0269) (Fig. 5*B*); only 15% of mice receiving ADC treatment were still colonized by day 7 compared to 40% and 42.1% for mNg001 and PBS, respectively (Fig. 5*B*). Mice treated with the ADC also had significantly reduced daily bacterial burden (Fig. 5*C*) and total area under the curve (AUC, Fig. 5*D*) compared to mNg001 alone (*P* = 0.004 and 0.0105, respectively) or the PBS control (*P* = 0.0156 and 0.0492, respectively) (Fig. 5 *C* and *D*). Together, these results demonstrate that the ADC accelerates clearance and reduces bacterial burden in a lower genital tract model of *N. gonorrhoeae* infection.

### *N. gonorrhoeae* Displays Low Levels of Resistance Development to Oct-TriA_1_-c.

**Fig. 6.**
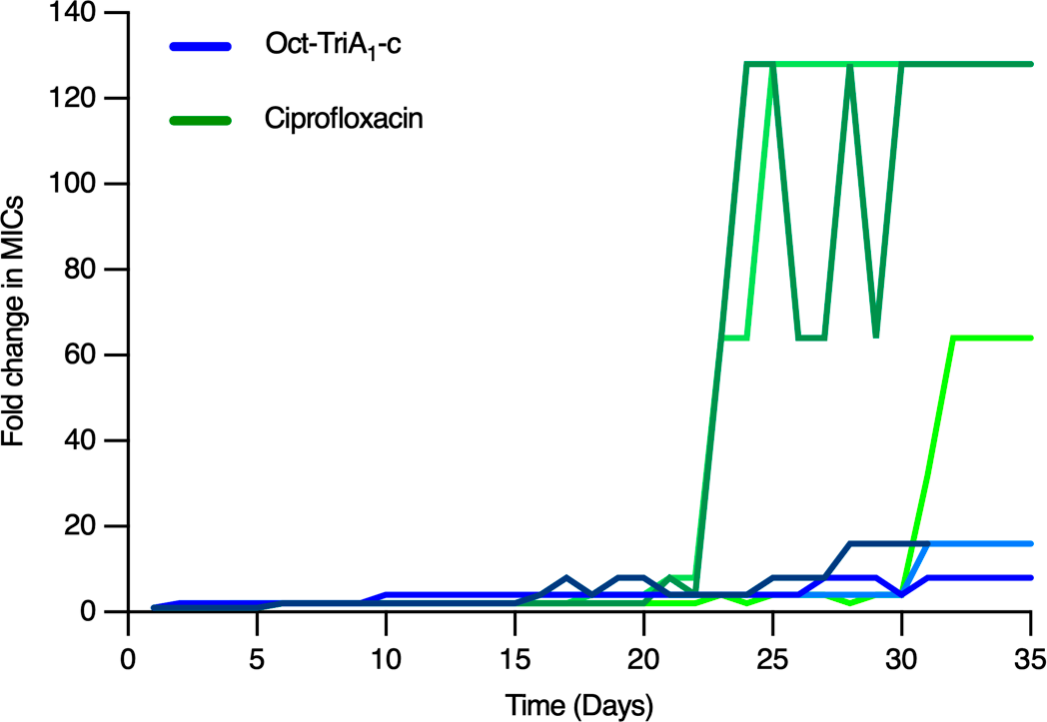
Limited evolution of resistance of *N. gonorrhoeae* to Oct-TriA_1_. Three colonies of *N. gonorrhoeae* were subjected to increasing concentrations of Oct-TriA_1_-c (blue) or ciprofloxacin (green) for 35 d. Data indicate fold-change in MIC at each timepoint.

Due to the propensity of *N. gonorrhoeae* to rapidly develop resistance to antimicrobials and the intrinsic resistance of *N. gonorrhoeae* to the AMP, polymyxin B due to the expression of EptA catalyzing the transfer of phosphoethanolamine (PEA) to the LOS lipid A (53). We evaluated the in vitro evolution of resistance of three *N. gonorrhoeae* FA1090 colonies after continuous exposure to sub-MIC concentrations of Oct-TriA1-c, the product after cleavage by the IgAP, or ciprofloxacin (Fig. 6). Our results demonstrate that the nucleic acid synthesis inhibitor ciprofloxacin had an 64- 128-fold change in MIC from 0.002 to 0.26 μg mL^−1^ over 35 d in-line with previous observations (54). In contrast, only low levels of resistance, 8- 16-fold increase in MIC, (1.56 to 12.5 μg mL^−1^) was observed for *N. gonorrhoeae* against Oct-TriA_1_-c indicating that in FA1090, resistance development to Oct-TriA_1_-c occurred at a significantly lower level than observed for ciprofloxacin under the same experimental conditions.

Taken together, our data demonstrate that Oct-TriA_1_ conjugated to an α-MtrE mAb *via* a IgAP cleavable linker offers targeted delivery of an AMP that efficiently kills MDR *N. gonorrhoeae* while reducing off-target cytotoxicity. The mechanism of action of this ADC is dependent on both its ability to bind MtrE on the bacterial surface, and the IgA protease secreted by the gonococcus as an immune evasion strategy.

## Discussion

*N. gonorrhoeae* is an urgent public health threat due to its increasing incidence and increasing AMR (2). Resistance has emerged in the gonococcus within a few years of introduction of every antibiotic (55). The rise in AMR not only undermines the treatment of patients with gonococcal infection but also jeopardizes the control of infection, which relies on successful treatment of their contacts (42, 56). Postexposure prophylaxis (PEP) is being used to protect individuals who have potentially been exposed to STIs after unprotected intercourse (57). Efforts to use doxycycline against gonorrhea have been less successful against the gonococcus than for other STIs due to plasmid-mediated AMR (47, 57). Therefore, there is an urgent need to develop alternative treatment options against this critically important human pathogen.

Tridecaptins are potent AMPs which we show are highly active against *N. gonorrhoeae* (33). Furthermore, our data indicate that the laboratory strain FA1090 has a relatively low propensity for developing resistance against Oct-TriA_1_ compared with ciprofloxacin, consistent with previous observations with *E. col*i (33). This contrasts with the intrinsic resistance of *N. gonorrhoeae* to polymyxin B, primarily due to PEA modification of lipid A of the LOS (53) which makes the tridecaptins an attractive class of AMPs. However, these cationic peptides also interact nonspecifically with lipid-containing membranes, leading to unwanted cytotoxicity and hemolytic activity (34). This inherent toxicity has limited the development of tridecaptins and other AMPs for treating bacterial infection. Instead, we repurposed these potent AMPs by conjugating them to a gonococcal specific mAb to deliver them directly to the bacterial surface. Although ADCs have been extensively used in oncology (58), only three mAbs have been approved for treating bacterial infection, all of which neutralize secreted toxins (59–61) rather than having direct antimicrobial activity. Efforts have been made to develop ADCs which are bactericidal against *S. aureus* and *P. aeruginosa* by incorporating a host cathepsin-cleavable linker to release a bactericidal payload (62, 63). Unfortunately, cathepsin is only activated in the phagolysosome so such a strategy would not be effective against extracellular pathogens. Instead, we engineered ADCs with a bacterially cleavable linker to allow release of a highly active AMP in close proximity to the gonococcus. This additional targeting mechanism led to an ADC with virtually no host cell cytotoxicity without compromising bactericidal activity.

The mAb in our ADC targets MtrE, which is conserved and constitutively expressed by *N. gonorrhoeae* (12). MtrE is the outer membrane channel of three gonococcal efflux pumps, MtrCDE, FarAB, and MacAB, which contribute to AMR (64). Importantly, the MtrCDE system is overexpressed by many MDR gonococcal strains (13, 42). The two surface exposed loops of MtrE are highly conserved and our mAb recognizes all 51 isolates tested to date, spanning the genetic diversity of the gonococcus (47, 65). In contrast to other mAbs which target the gonococcus (52, 66, 67), mNg001 does not enhance clearance of FA1090 in the lower genital tract model of infection. The lack of in vivo activity for mNg001 might reflect a low abundance of the antibody epitope, impairing complement effector functions (68), or the isotype of the mAb (IgG1) which has limited ability to activate the classical complement pathway (69).

Direct conjugation of Oct-TriA_1_ to the mAb did not result in bactericidal activity suggesting that release of the tridecaptin is required. We investigated the linear peptide, LPRPPAPVFS, as a bacterial-cleavable linker containing an IgAP cleavage site to couple Oct-TriA_1_ to mNg001 (49). The IgAP is a sophisticated immune evasion mechanism deployed by the gonococcus which specifically cleaves hIgA1, although it has also been shown to proteolytically cleave the lysosomal protein LAMP-1 (9, 70, 71). Therefore, mutants which escape killing by the ADC by not expressing MtrE or the IgAP will be more susceptible to other antibiotics or local immune responses, respectively. Although rare MtrE variants lacking the mNg001 epitope could evade antibody recognition while retaining function, our analysis indicates that the predicted epitope, in MtrE loop 2, is conserved in >98% of gonococcal isolates. Therefore, such variants might be uncommon but highlight the need for enhanced surveillance to monitor resistance development after implementation of any ADC.

To synthesize the ADC, we identified a site on Oct-TriA_1_ for its attachment to the mAb. The length of the acyl tail and several residues [i.e., D-Trp^5^, D-Dab (8), and D-*allo*-Ile (12)] are critical for the antimicrobial activity of Oct-TriA_1_ (34). Glu^10^ of an unacylated tridecaptin analogue has been modified for addition of erythromycin and vancomycin analogues against *K. pneumoniae* (72), but the toxicity of these conjugates against host cells is unknown. We found that a 10 amino acid linker (LPRPPAPVFS) can be added to the C-terminal Ala of Oct-TriA_1_ with a negligible effect on antimicrobial activity of the AMP (Table 1). *cl*-Oct-TriA_1_ was covalently conjugated to our gonococcal-specific, α-MtrE mAb by maleimide cross-linking between a C-terminal Cys group on *cl*-Oct-TriA_1_ and lysine residues on the mAb (73). This resulted in an average DAR of 5 to 8.

Time-kill curves demonstrate that the ADCs with a cleavable linker are bactericidal against *N. gonorrhoeae* in an MtrE- and IgAP-dependent manner. Furthermore, the cleavable ADC kills the highly resistant isolate, G97687, which is resistant to all first line agents (42). In contrast, ADCs with an uncleavable linker had no bactericidal activity at lower concentrations (50 μg mL^−1^), but at higher concentrations (150 μg mL^−1^) exhibited nonspecific bactericidal activity. NHS-esters form partially stable linkages which can be hydrolyzed in aqueous environments with spontaneous release of the AMP (73). A minor peak in the ESI-MS spectra (m/z = 804) indicates nonspecific hydrolysis of uncl-Oct-TriA_1_ between Pro and Ala (VKPAPSP^AAN) (*SI Appendix*, Fig. S7). This is most likely due to instability of the peptide; previous analysis of this peptide showed no detectable cleavage after 24 h in the presence of IgAP (49). Linker stability is critical for maintaining specificity and minimizing off-target peptide release. In the future, specific AMP:mAb linkages will be generated by engineering the AMP and mAbs, resulting in a similar yet defined DAR and known sites of conjugation (36, 62).

Our strategy for developing ADCs against *N. gonorrhoeae* is modular, allowing selection of different mAbs, linkers, and AMPs. The mAb we employed is recognized by the five most common variants of gonococcal MtrE without binding MtrE from other *Neisseria* spp., providing precise targeting of the ADC to the gonococcus. Additionally, commensal *Neisseria* spp. rarely express the IgAP (*SI Appendix*, Table S6) making it unlikely that Oct-TriA_1_ is inadvertently released from the ADC by this group of bacteria. The meningococcus does express an IgAP related to the gonococcal enzyme so could cleave the ADC although *N. meningitidis* MtrE is not recognized by mNg001; the IgAP from other genera recognize other amino acid sequences (74). Thus, the mechanism of release of the AMP provides another layer of specific activity for the ADC, which should be active against the gonococcus irrespective of their site in the host as it relies on a constitutively expressed bacterial immune escape mechanism and not a host protease.

In conclusion, our findings indicate that our ADC is a potent antimicrobial against MDR *N. gonorrhoeae* with virtually undetectable host cell cytotoxicity or hemolytic activity. We have exploited two key virulence factors of the gonococcus, MtrE and IgAP, which have important roles in gonococcal AMR and pathogenesis. This ADC, containing a linker which is cleaved by a specific bacterial protease, should induce minimal disruption of the microbiome, which is an advantage over other small molecule therapeutics, antibiotics, and AMPs. Finally, our ADCs can also be utilized as a platform for the development of other ADCs targeting other bacterial species as the ADC platform is entirely modular so the components can be exchanged to target other organisms.

## Materials and Methods

Detailed methods are provided in *SI Appendix*, *Appendix 1*. *N. gonorrhoeae mtrE* sequences were downloaded from PubMLST. Gonococcal base (GCB) (75), and bacteria grown in fastidious broth (FB) (76, 77), and Graver and Wade medium (GW) (78) were prepared as previously, and supplemented with 1% Vitox; 1.5% (w/v) agar (Oxoid) was added for solid media. Sequences up- and down-stream fragments of *mtrE* and *igaP* were amplified using Herculase II Fusion DNA Polymerase (Agilent); primers were designed with the NEBuilder® assembly tool. Amplified fragments were inserted into pUC19 flanking a kanamycin resistance cassette using Gibson Assembly® master mix (New England Biolabs) then transformed into *E. coli* DH5α. To generate mAbs, female BALB/c mice (6 to 8 wk old; Charles River, Margate, UK) were immunized with recombinant MtrE (20 μg) adsorbed to aluminum hydroxide (final composition, 0.5 mg mL^−1^ Al-OH_3_, 10 mM histidine-HCl) by mixing overnight at 4 °C. Antigens were given by the intraperitoneal route on days 0, 21, and 35. Spleens and sera were harvested on day 49. Animal experiments were conducted according to protocols approved by the Uniformed Services University’s Institutional Animal Care and Use Committee. Female BALB/c mice (6–8 wk old; Charles River Laboratories) were randomized and synchronized in diestrus or the anestrus stage prior to infection.

## Supplementary Material

Appendix 01 (PDF)

## Data Availability

All study data are included in the article and/or *SI Appendix*.
