# Supplementary material for: An antibody–drug conjugate active against antibiotic-resistant Neisseria gonorrhoeae
Source: Proc Natl Acad Sci U S A. 2026 Jul 28;123(31):e2534217123. doi: 10.1073/pnas.2534217123 (PMC13439108; doi:10.1073/pnas.2534217123)
Supplement: Supplementary file 1 [file pnas.2534217123.sapp.pdf]

SUPPORTING INFORMATION FOR:

An antibody-drug conjugate active against antibiotic-resistant *Neisseria gonorrhoeae*

Hayley Lavender[a, b], Vincent Oliver[c], Daniel Lucy[d, e] Timothy. A. Barendt[d, f], Ann E. Jerse[g], Christoph M. Tang[a*,]

Author affiliations: [a] Sir William Dunn School of Pathology, University of Oxford, South Parks Road, Oxford OX1 3RE, United Kingdom;

[b] The Dept of Clinical Infection, Microbiology, and Immunology, Institute of Infection and Global Health, University of Liverpool, Liverpool L69 7BE, United Kingdom;

[c] Henry M. Jackson Foundation for the Advancement of Military Medicine, Bethesda, MD 20817, USA

[d] Department of Chemistry, University of Oxford, Mansfield Road, Oxford OX1 3TA, United Kingdom;

[e] Current affiliation, Department of Chemistry, Imperial College London, South Kensington Campus, London SW7 2AZ, United Kingdom;

[f] School of Chemistry, University of Birmingham, Edgbaston, Birmingham B15 2TT, United Kingdom

[g] Uniformed Services, University of the Health Sciences, Bethesda, MD 20814, USA

[*]corresponding author: Christoph Tang

E.mail: christoph.tang@path.ox.ac.uk

APPENDIX 1

MATERIALS AND METHODS

MtrE *and* IgAP *analyses*

N. gonorrhoeae mtrE sequences were downloaded from PubMLST(1) (https://pubmlst.org/organisms/neisseria-spp, accessed June/July 2023), translated, then aligned by Muscle in Geneious Prime 2023. The number of isolates associated with each single amino acid polymorphism (SAAP) was imported into GraphPad Prism v10.2.2, then mapped onto the structure of MtrE (PDB accession no. 4MT0)(2). MtrE conservation across commensal *Neisseria* spp. was analysed, and the presence or absence of the IgAP were evaluated among the commensal *Neisseria* spp. listed in **Supplementary Table 5** (https://pubmlst.org/organisms/neisseria-spp, accessed January 2026).

Bacterial strains and growth

Gonococcal base (GCB)(3), Fastidious broth (FB)(4, 5), and Graver and Wade medium (GW)(6) were prepared as previously, and supplemented with 1% Vitox; 1.5% (w/v) agar (Oxoid) was added for solid media. *N. gonorrhoeae* strains (**Supplementary Table 7**) were grown on GCB agar for 16–18 hr at 37°C with 5% CO_2. For liquid growth, bacteria were inoculated into 125 ml conical flasks containing 20 ml of liquid medium at an OD_{A600} of 0.1, then incubated in 5% CO_2 at 37°C shaking at 150 r.p.m.; kanamycin was added to media at 80 μg ml^{-1} as required. *Escherichia coli* was grown in Luria-Bertani (LB) broth, Terrific broth (TB), or on LB with 1.5% (w/v) agar. Antibiotics were added at the following concentrations for *E. coli*: kanamycin, 50 μg ml^{-1}, and carbenicillin, 100 μg ml^{-1}.

*Generation of Δ*mtrE *and ΔigaP strains*

Genomic DNA was isolated using the Wizard Genomic DNA purification kit (Promega), and up- and down-stream fragments of *mtrE* and *igaP* were amplified using Herculase II Fusion DNA Polymerase (Agilent); primers (**Supplementary Table 8**) were designed with the NEBuilder® assembly tool.

Amplified fragments were inserted into pUC19 flanking a kanamycin resistance cassette using Gibson Assembly® master mix (New England Biolabs) then transformed into *E. coli* DH5α. Plasmids were confirmed by sequencing, digested with *Aat*II and *Bsa*I prior to transforming *N. gonorrhoeae* as previously described(3).

Generation of α-MtrE mAbs

mtrE was amplified from *N. gonorrhoeae* FA1090 and ligated into pET14b, generating pET14b:*mtrE*. *E. coli* B834 containing pET14b:*mtrE* was grown in TB for 24 h at 22°C and harvested by centrifugation at 5,000 × *g* for 30 min at 4°C prior to lysis using an EmulsiFlex-C5 homogenizer (Avestin, 15,000 lb/in^2). Lysates were centrifuged at 20,000 × *g* for 30 min at 4°C, and recombinant MtrE was bound to a HisTrap column (GE Healthcare), eluted with 300 mM imidazole, and purified by size exclusion (AKTA, HiLoad 16/600 Superdex® 200 pg column; GE Healthcare). Protein concentrations were estimated using a Nanodrop 2000c {Thermo Scientific).

Female BALB/c mice (6 to 8 weeks old; Charles River, Margate, UK) were immunised with recombinant MtrE (20 µg) adsorbed to aluminium hydroxide (final composition, 0.5 mg ml^{-1} Al-OH$_3$, 10 mM histidine-HCl) by mixing overnight at 4°C. Antigens were given by the intraperitoneal route on days 0, 21, and 35. Spleens and sera were harvested on day 49. B lymphocytes were fused to NS0 myeloma cells with polyethylene glycol (PEG 1500, Roche). Fused cells were plated in RPMI 1640 containing 2 mM L-glutamine, penicillin (100 U ml^{-1}), streptomycin (100 µg ml^{-1}), and 1% Ultroser G (Pal France). After 24 hr, 2% hypoxanthine aminopterin thymidine (Life technologies) was added to the wells. Hybridomas were screened by ELISA against recombinant MtrE then cloned by limiting dilution. Animal experiments were carried out under protocols approved by the Home Office UK (PPL 30/3194). mAbs were purified from hybridoma supernatants in Gibco® PFHM II serum-free media supplemented

with 2% (w/v) cholesterol by protein G affinity chromatography (Pierce, ThermoFisher Scientific). mAb isotypes were determined using a Mouse Monoclonal Antibody Isotyping Test Kit (Bio-Rad).

SDS-PAGE and Western blot analysis

Bacteria were grown overnight and 10^9 CFU ml^{-1} suspended in SDS-PAGE loading buffer (100 mM Tris-HCl, pH 6.8, 20 µM β-mercaptoethanol, 4% SDS, 0.2% bromophenol blue, 20% glycerol) prior to separation on 14% polyacrylamide gels. Proteins were transferred to Immobilon P polyvinylidene difluoride membranes (Millipore, USA) using the Trans-Blot SD semi-dry transfer system (Bio-Rad, USA), then blocked in 3% (w/v) skimmed milk-PBS with 0.05% (v/v) Tween 20 overnight and incubated with hybridoma supernatants or anti-MtrE mAb (5 µg ml^{-1}) . Membranes were washed three times in PBS with 0.05% (v/v) Tween 20, and incubated with goat anti-mouse HRP (P0447, Dako, UK), washed again, and binding detected using ECL (Amersham, USA).

Flow cytometry

Approximately 10^8 CFU ml^{-1} of bacteria were re-suspended in mAb (5 µg ml^{-1}) for 30 min, washed in wash buffer (0.05% (w/v) BSA/PBS), then incubated for with 2 µg ml^{-1} goat anti-mouse IgG-Alexa fluor® 647 conjugate (A-21235, Life Technologies) at room temperature. Samples were washed three times then fixed in 3% paraformaldehyde for 30 min, washed three times then resuspended in PBS prior to running on a FACSCalibur (BD Biosciences) or BD LSRFortessa™ Cell Analyzer. Results were analysed by calculating the geometric mean fluorescence (FL-4) in FlowJo v10.10 software (Tree Star). Statistical significance was tested using an unpaired Student *t* test or 2-way ANOVA (GraphPad Prism v.10.2.2).

Conjugation of Oct-TriA$_1$ derivatives with mAbs

m-maleimidobenzoyl-*N*-hydoxysuccinimide (MBS, 5 mM) was added to purified mAb (0.1 mM) and incubated at room temperature for 60 min. Excess MBS was removed over ZebraTM Spin desalting

columns (ThermoFisher scientific). Activated mAb was added to 0.9 mg of Oct-TriA$_1$ analogues (synthesised by ISCA Biochemicals, UK) and incubated at room temperature for 60 min. ADCs were dialysed using slide-A-Lyzer cassette (20K MWCO, ThermoFisher scientific) into PBS overnight at 4°C and filter sterilised.

MALDI-TOF MS analysis for determination of intact mass

mAbs or ADCs with a concentration of 1 mg/mL were acidified with 1% trifluoroacetic acid and mixed with Super DHB matrix, 1 µl was spotted onto an MTP Anchorchip 600/384. For every mAb, three sample spots were prepared from the same solution using three protocols advised by the manufacturer. MALDI-TOF measurements were performed on a Microflex LRF mass spectrometer, equipped with a 60Hz nitrogen laser at 337nm. Number of peptides conjugated was calculated using the MALDI-TOF molecular weights (MW) [MW = M^+] of each ADC, the unconjugated antibody (mAb), and the molecular weights of Oct-TriA$_1$ analogues using the following equation.

$$\text{Number of peptides} = \frac{\text{MW ADC} - \text{MW mAb}}{\text{MW peptide}}$$

Cleavage and detection of Oct-TriA$_1$ derivatives and ADC activity

IgA1 protease cleavage was assayed using supernatants from *N. gonorrhoeae* grown for 24 hr in FB medium that were subsequently dialysed into PBS (slide-A-Lyzer, 20K MWCO, ThermoFisher scientific). Peptides (50 µg) or ADCs (750 µg) were incubated for 4 hr at 37°C with the dialysed supernatants. Samples were desalted then analysed by mass spectrometry with a Waters Micromass LCT Mass spectrometer using electrospray ionization (ESI-MS).

Determination of Oct-TriA$_1$ Minimum Inhibitory Concentration (MIC)

N. gonorrhoeae was resuspended in PBS to an OD_{A600} 1.0, and ~5 × 10^4 CFU added to wells of a microdilution plate containing dilutions of antimicrobials in FB. Plates were incubated for 24 hr at

37°C, in 5% CO_2 then 10 µl aliquots plated onto GCB agar. The MIC was defined as the lowest concentration of an antimicrobial that prevented bacterial growth.

Determination of* N. gonorrhoeae *resistance to Oct-TriA$_1$

Single colonies of *N. gonorrhoeae* FA1090 were inoculated into 2 mL of FB media supplemented with 1% Vitox (Oxoid) and grown overnight. After 24 hours, 240 µl of a 1/ 5,000 dilution of FA1090 in 2x FB was added to a 48-well plate containing 2-fold dilutions of either Oct-TriA$_1$-*c* (final concentration, 12.5 µg/ml to 0.2 µg/ml) or ciprofloxacin (final concentration, 16.4 to 0.001 µg/ml) in a total volume of 480 µl. Plates were incubated at 37 °C, 5% CO_2 with shaking (180 rpm). After 24 hours, OD_{A600} of each well was quantified and MIC determined as less than 50% growth compared to no antimicrobial control. Aliquots from the highest antimicrobial concentration that displayed growth were diluted 1/ 10,000 to fresh media containing all concentrations of antimicrobials for a total of 35 days.

Peptide concentration in ADC preparations

Peptide concentrations for ADCs were calculated from the antibody concentration, the drug-to-antibody ratio (DAR), and the peptide molecular weight using the following formula:

$$\text{Conc. peptide } (\mu g/mL) \;=\; \text{Conc. ADC } (\mu g/mL) \;\times\; \frac{\text{DAR x MWpeptide (Da)}}{\text{MW}_{\text{ADC}}\text{(Da)}}$$

Where Conc. ADC is the ADC/ mAb concentration, MW_{ADC} is the mAb molecular weight (150 kDa), MW*peptide* is the peptide molecular weight and DAR is the average number of peptides per conjugate. This formula was used to convert ADC concentrations to peptide concentrations for all assays.

Time-to-kill assays

For time-kill assays, bacterial suspensions (10^8 CFU) were diluted in GW medium, and 30 µl added to 7.5 ml pre-warmed GW medium. A total of 90 µl of the suspension was added per well (Nunclon™ microtiter plates, ThermoFisher scientific), then incubated for 3 h. Next 110 µl of an antimicrobial or

media alone was added to each well and incubated for 4 hr. Samples (10 µl) were plated every 30 min in triplicate onto solid media. Peptides were added at a final concentration of 50 µg ml^{-1} and ADCs at 0.75 µg ml^{-1} for FA1090 or 1.5 µg ml^{-1} for G97687.

Cytotoxicity assays

For hemolysis, whole human blood (1 ml; K2EDTA; Cambridge Bioscience) was washed in PBS then 0.5% added to an equal volume of peptides or ADCs in 96-well V-bottomed polypropylene plates and incubated at 37°C for 1 hr. Cells were pelleted at 1,000 × *g* for 10 min and the absorbance at OD_{415} measured. Positive (melittin, 2.5 µM, Merck Life Science) and negative (PBS) controls were normalised as 100 and 0% hemolysis, respectively.

HK-2 cells (CRL-2190™, Americab Type Culutre Collection (ATCC)), a proximal renal tubule cell(7), and End1/E6E7 (CRL-2615™, ATCC) upper cervical epithelial cells(8) were cultured in Keratinocyte Serum Free Medium (Invitrogen, Life Technologies Ltd.) with human recombinant EGF (5 ng ml^{-1}) and bovine pituitary extract (0.05 mg ml^{-1}) at 37°C in 5% CO_2. Cells were seeded in 96-well plates (Corning, Polystyrene) at a density of 2×10^4 cells/well 24 hr prior; Triton-X-100 (0.1%) and Digitonin (30 µg ml^{-1}) were used as controls. Lactate dehydrogenase (LDH) release was measured using the Biovision kit; the OD_{490} was assessed in a SpectraMax M5 plate reader.

Cell viability was assessed by 3-(4,5-dimethylthiazol-2-yl)-2,5-diphenyl-2H-tetrazolium bromide (MTT) assay(9); 200 µl fresh cell media and 250 µg of MTT was added to cells. Plates were Incubated at 37 °C, 5% CO_2 for 3 hr then 200 µl of DMSO was added to wells with 25 µl glycine buffer (0.1 M glycine, 0.1 M NaCl pH 10.5) and shaken at 1000 r.p.m. for 10 min. Absorbance was read at OD_{570} nm. Cell viability was calculated as (sample absorbance – cell free blank/media control absorbance) x100.

Murine model of* N. gonorrhoeae *infection

Animal experiments were conducted according to protocols approved by the Uniformed Services University's Institutional Animal Care and Use Committee. Female BALB/c mice (6–8 weeks old; Charles River Laboratories) were randomised and synchronised in diestrus or the anestrus stage prior to infection. To enhance susceptibility to *N. gonorrhoeae* colonisation, mice were implanted subcutaneously with a 5 mg slow-release 17-β estradiol pellet (Innovative Research of America) and to limit commensal overgrowth, mice received streptomycin (24 mg ml^{-1}) and vancomycin (4 mg ml^{-1}) by intraperitoneal injection (0.2 ml on day −2 and -1; 0.15 ml on days 0 and +1), and trimethoprim (0.4 g l^{-1}) in drinking water through day +1; from day +2 onward, trimethoprim (0.4 g l^{-1}) and streptomycin (5 g l^{-1}) were provided in drinking water. Two days after 17-β estradiol implantation, mice were inoculated intravaginally with 20 µL of *N. gonorrhoeae* strain FA1090 (~1×10^6 CFU). For efficacy studies, mNg001 or ADC were administered intravaginally daily starting 2 hr prior to infection and continued daily 1 h after sampling throughout the experiment; control group received PBS. mAbs/ ADCs were administered at 30 µg/ day in 20 µl. Vaginal swabs were cultured on GCB plates with VCNT with streptomycin using a Spiral Plater (Interscience). Clearance was measured as percentage of culture-positive mice post-inoculation and analysed by the Log-Rank (Mantel-Cox) test. CFU/ ml recovered from each group was compared by 2-way ANOVA with Bonferroni's multiple comparisons test. Kruskal-Wallis's test with Dunn's multiple comparison test were employed to assess the total area under the curve (AUC) over seven days.

REFERENCES

1. K. A. Jolley, J. E. Bray, M. C. J. Maiden, Open-access bacterial population genomics: BIGSdb software, the PubMLST.org website and their applications. *Wellcome Open Res* 3, 124 (2018).
2. H. T. Lei *et al.*, Crystal structure of the open state of the Neisseria gonorrhoeae MtrE outer membrane channel. *PLoS One* 9, e97475 (2014).
3. J. P. Dillard, Genetic Manipulation of Neisseria gonorrhoeae. *Curr Protoc Microbiol* Chapter 4, Unit4A.2.16 (2011).
4. C. P. Cartwright, F. Stock, V. J. Gill, Improved enrichment broth for cultivation of fastidious organisms. *J Clin Microbiol* 32, 1825-1826 (1994).
5. S. Kuss *et al.*, Versatile Electrochemical Sensing Platform for Bacteria. *Anal Chem* 91, 4317-4322 (2019).
6. J. J. Wade, M. A. Graver, A fully defined, clear and protein-free liquid medium permitting dense growth of Neisseria gonorrhoeae from very low inocula. *FEMS Microbiol Lett* 273, 35-37 (2007).
7. M. Vaara, T. Vaara, The novel polymyxin derivative NAB739 is remarkably less cytotoxic than polymyxin B and colistin to human kidney proximal tubular cells. *Int J Antimicrob Agents* 41, 292-293 (2013).
8. R. N. Fichorova, P. J. Desai, F. C. Gibson, C. A. Genco, Distinct proinflammatory host responses to Neisseria gonorrhoeae infection in immortalized human cervical and vaginal epithelial cells. *Infect Immun* 69, 5840-5848 (2001).
9. M. J. Santander-Ortega *et al.*, Nanoparticles made from novel starch derivatives for transdermal drug delivery. *J Control Release* 141, 85-92 (2010).

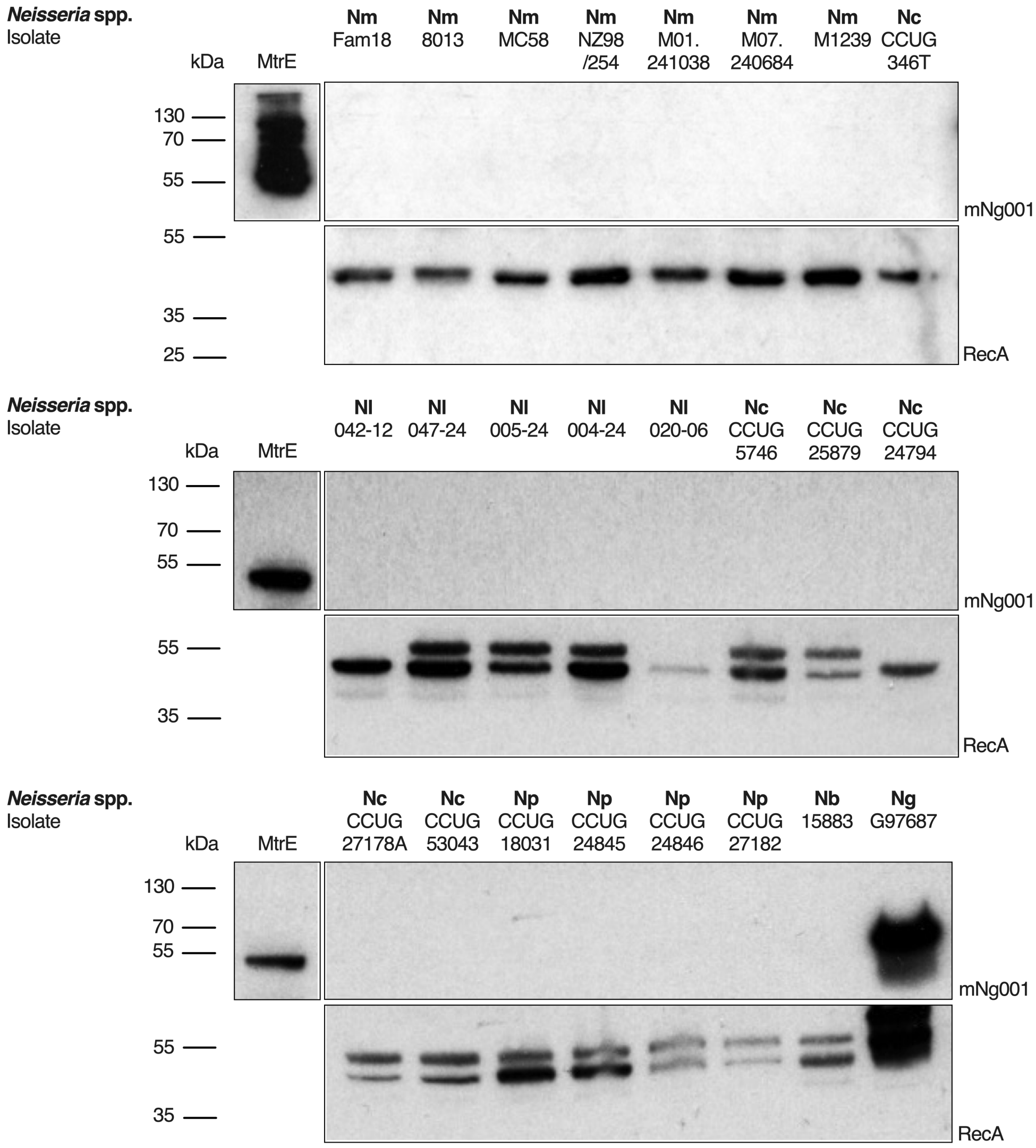

Supplementary Figure 1. Monoclonal antibody, mNg001 does not recognise MtrE from *Neisseria meningitidis* or non-invasive *Neisseria* spp. Western blot analysis for recognition of MtrE from; *N. meningitidis* (Nm), *Neisseria lactamica* (Nl), *Neisseria cinerea* (Nc), *Neisseria polysaccharea* (Np) and *Neisseria bergeri* (Nb) using mAb, mNg001. Recombinant MtrE and wild-type MDR gonococcal strain (Ng), G97687 were used as positive controls; RecA used as a loading control.

```
Neisseria_gonorrhoeae|G97687|          MNTTLKTTLTSVAAAFALSACTMIPQYEQPKVEVAETFQNDTSVSSIRAVDLGWHDYFAD   60
Neisseria_meningitidis|MC58|           MDTTLKTTLTSVAAAFALSACTMIPQYEQPKVEVAETFKNDTADSGIRAVDLGWHDYFAD   60
Neisseria_lactamica|020-06|            MNTTLKTTLTSVAAAFALSACTMIPQYGQPKVEVAETFKNDTADSGIRAVDLGWHDYFAD   60
Neisseria_cinerea|CCUG_346T|           MNTTLKTTLTSVAAAFALSACTMIPQYEQPKVEVAETFKNDTADSGIRAVDLGWHDYFAD   60
Neisseria_polysaccharea|CCUG_18031|    MNTTLKTTLTSVAAAFALSACTMIPQYEQPKVEVAETFKNDTADSGIRAVDLGWHDYFAD   60
Neisseria_bergeri|15883|               MDTTLKTTLTSVAAVFALSACTMIPQYEQPKVEVAETFKNDTADSGIRAVDLGWHDYFAD   60
                                       *:************.************ **********:***: *.**************

Neisseria_gonorrhoeae|G97687|          PRLQKLIDIALERNTSLRTAVLNSEIYRKQYMIERNNLLPTLAANANGSRQGSLSGGNVS   120
Neisseria_meningitidis|MC58|           PRLQKLIDIALERNTSLRTAVLNSEIYRKQYMIERNNLLPTLAANANDSRQGSLSGGNVS   120
Neisseria_lactamica|020-06|            PRLQKLIDIALERNTSLRTAVLNSEIYRKQYMIERNNLLPTLAANANGSRQGSLDGGNVS   120
Neisseria_cinerea|CCUG_346T|           PRLQKLIDIALERNTSLRTAVLNSEIYRKQYMIERNNLLPTLAANAGGSRQGSLSGGNVS   120
Neisseria_polysaccharea|CCUG_18031|    PRLQKLIDIALERNTSLRTAVLNSEIYRKQYMIERNNLLPTLAANANGSRQGSLDGGNVS   120
Neisseria_bergeri|15883|               PRLQKLIDIALERNTSLRTAVLNSEIYRKQYMIERNNLLPTLAANANGSRQGSLSGGNVS   120
                                       **********************************************..******.*****

Neisseria_gonorrhoeae|G97687|          SSYNVGLGAASYELDLFGRVRSSSEAALQGYFASVANRDAAHLSLIATVAKAYFNERYAE   180
Neisseria_meningitidis|MC58|           SSYKVGLGAASYELDLFGRVRSSSEAALQGYFASTANRDAAHLSLIATVAKAYFNERYAE   180
Neisseria_lactamica|020-06|            SSYNVGLGAASYELDLFGRVRSSSEAALQGYFASTANRDAAHLSLIATVAKAYFNERYAE   180
Neisseria_cinerea|CCUG_346T|           SSYNVGLGAASYELDLFGRVRSSSEAALQGYFASVANRDAAHLSLIATVAKAYFNERYAE   180
Neisseria_polysaccharea|CCUG_18031|    SSYNVGLGAASYELDLFGRVRSSSEAALQGYFASVANRDAAHLSLIATVAKAYFNERYAE   180
Neisseria_bergeri|15883|               SSYNVGLGAASYELDLFGRVRSSSEAALQGYFASVANRDAAHLSLIATVAKAYFNERYAE   180
                                       ***:******************************.*************************

Neisseria_gonorrhoeae|G97687|          EAMSLAQRVLKTREETYKLSELRYKAGVISAVALRQQEALIESAKADYAHAARSREQARN   240
Neisseria_meningitidis|MC58|           EAMSLAQRVLKTREETYKLSELRYKAGVISAVALRQQEALIESAKADYAHAARSREQARN   240
Neisseria_lactamica|020-06|            EAMSLAQRVLKTREATYKLSELRYKAGVISAVALRQQEALIESAKADYANAVRSREQARN   240
Neisseria_cinerea|CCUG_346T|           EAMSLAQRVLKTREATYKLSELRYKAGVISAVALRQQEALIESAKADYANAVRSREQARN   240
Neisseria_polysaccharea|CCUG_18031|    EAMSLAQRVLKTREETYKLSELRYKAGVISAVALRQQEALIESAKADYAHAARSREQARN   240
Neisseria_bergeri|15883|               EAMSLAQRVLKTREETYKLSELRYKAGVISAVALRQQEALIESAKADYANAVRSREQARN   240
                                       ************** **********************************:*.********

Neisseria_gonorrhoeae|G97687|          ALATLINRPIPEDLPAGLPLDKQFFVEKLPAGLSSEVLLDRPDIRAAEHALKQANANIGA   300
Neisseria_meningitidis|MC58|           ALATLINQPIPEDLPAGLPLDKQFFVEKLPAGLSSEVLLDRPDIRAAEHALKQANANIGA   300
Neisseria_lactamica|020-06|            ALATLINQPIPDDLPAGLPLDKQFFVEKLPAGLSSEVLLDRPDIRAAEHALKQANANIGA   300
Neisseria_cinerea|CCUG_346T|           ALATLINQPIPNDLPAGLPLDKQFFVEKLPAGLSSEVLLDRPDIRAAEHALKQANANIGA   300
Neisseria_polysaccharea|CCUG_18031|    ALATLINQPIPDDLPAGLPLDKQFFVEKLPAGLSSEVLLDRPDIRAAEHALKQANANIGA   300
Neisseria_bergeri|15883|               ALATLINQPIPDDLPAALPLDKQFFVEKLPAGLSSEVLLDRPDIRAAEHALKQANANIGA   300
                                       *******:***:****.*******************************************

Neisseria_gonorrhoeae|G97687|          ARAAFFPSIRLTGSVGTGSVELGGLFKSGTGVWAFAPSITLPIFTWGTNKANLDVAKLRQ   360
Neisseria_meningitidis|MC58|           ARAAFFPSIRLTGTVGTGSAELGGLFKSGTGVWSFAPSITLPIFTWGTNKANLDVAKLRQ   360
Neisseria_lactamica|020-06|            ARAAFFPSIRLTGSVGTGSAELGGLFKSGTGVWSFAPSITLPIFTWGTNKANLDAAKLRR   360
Neisseria_cinerea|CCUG_346T|           ARAAFFPSIRLTGSVGTGSTELSGLFKSGTGVWSFAPSITLPIFTWGTNKANLDAAKLRQ   360
Neisseria_polysaccharea|CCUG_18031|    ARAAFFPSIRLTGSVGTGSAELGGLFKSGTGVWSFAPSITLPIFTWGTNKANLDIAKLRQ   360
Neisseria_bergeri|15883|               ARAAFFPSIRLTGSVGTGSAELGGLFKSGTGVWSFAPSITLPIFTWGTNKANLDVAKLRQ   360
                                       *************:*****.**.**********:******************** ****:

Neisseria_gonorrhoeae|G97687|          QAQIVAYESAVQSAFQDVANALAAREQLDKAYDALSKQSRASKEALRLVGLRYKHGVSGA   420
Neisseria_meningitidis|MC58|           QVQIVAYESAVQSAFQDVANALAAREQLDKAYDALSKQSRASKEALRLVGLRYKHGVSGA   420
Neisseria_lactamica|020-06|            QAQIVAYEAAVQSAFQDVANALAAREQLDKAYDALSKQSRASKEALRLVGLRYKHGVSGA   420
Neisseria_cinerea|CCUG_346T|           QAQIVAYESAVQSAFQDVANALTAREQLDKAYAALSKQSRAYKEALRLVGLRYKHGVSGA   420
Neisseria_polysaccharea|CCUG_18031|    QAQIVAYEAAVQSAFQDVANALTAREQLDKAYAALSKQSRAYKEALRLVGLRYKHGVSGA   420
Neisseria_bergeri|15883|               QAQIVAYESAVQSAFQDVANALTAREQLDKAYAALSKQSRASKEALRLVGLRYKHGVSGA   420
                                       *.******:*************:********* ******** ******************

Neisseria_gonorrhoeae|G97687|          LDLLDAERISYSAEGAALSAQLTRAENLADLYKALGGGLKRDTQTGK   467
Neisseria_meningitidis|MC58|           LDLLDAERSSYAAEGAALSAQLTRAENLADLYKALGGGLKRDTQTDK   467
Neisseria_lactamica|020-06|            LDLLDAERSSYSAEGAALSAQLTRAENLADLYKALGGGLKRDTQTDK   467
Neisseria_cinerea|CCUG_346T|           LDLLDAERSSYSAEGAVLSAQLTRAENLADMYKALGGGLKRDTQTGK   467
Neisseria_polysaccharea|CCUG_18031|    LDLLDAERSSYSAEGAALSAQLTRAENLADLYKALGGGLKRDTQTGK   467
Neisseria_bergeri|15883|               LDLLDAERSSYAAEGAALSAQLTRAENLADLYKALGGGLKRDTQTGK   467
                                       ******** **:****.*************:**************.*
```

Supplementary Figure 2. MtrE from *N. gonorrhoeae* shows consistent sequence differences compared to other *Neisseria* spp. Sequence alignment of MtrE from a representative strain from different Neisseria spp. compared to MtrE from *N. gonorrhoeae* strain G97687, the most common gonococcal MtrE protein variant. Red residues indicate the two surface exposed loops whereas blue residues indicate consistent sequence differences from gonococcal MtrE.

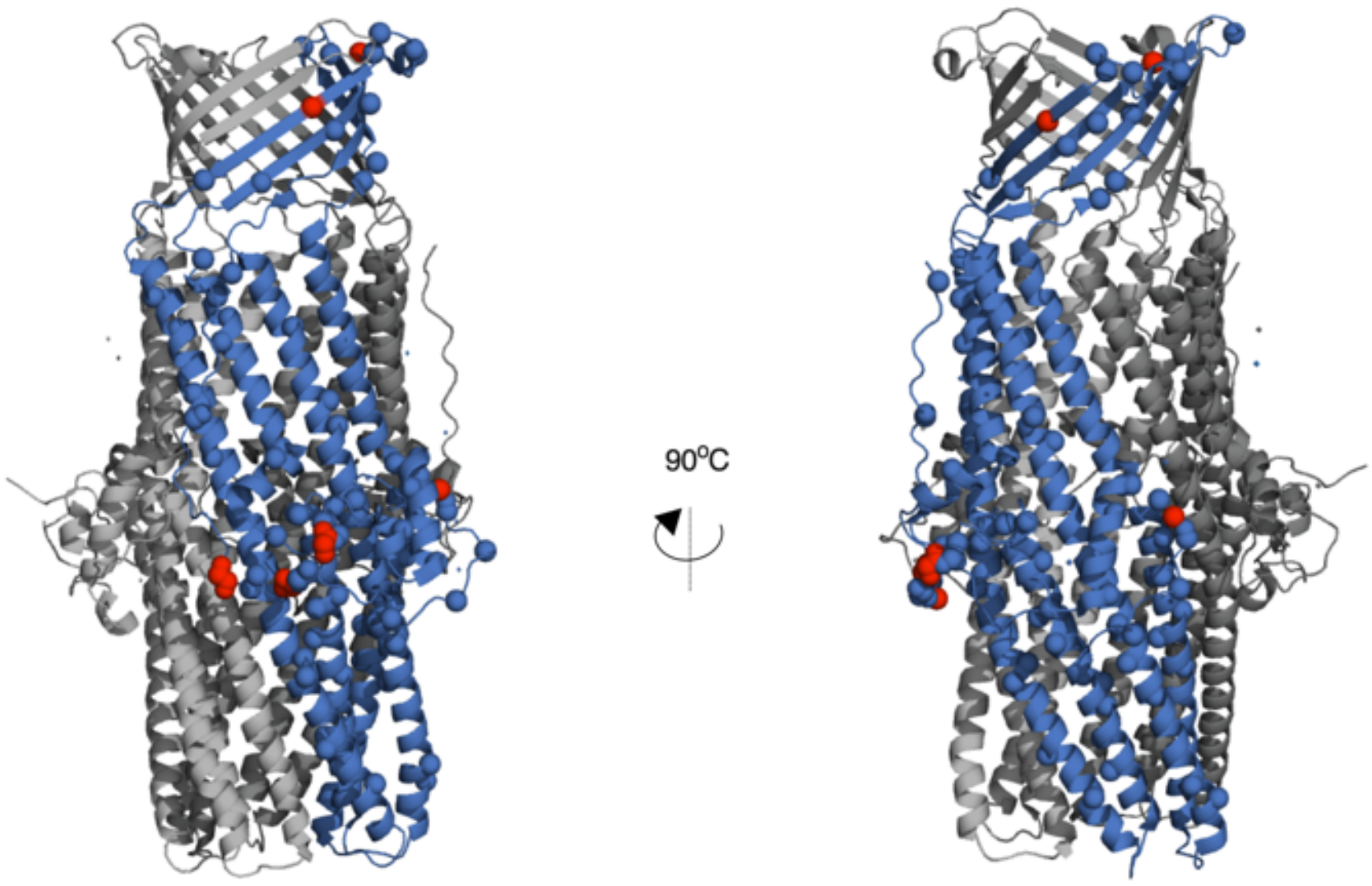

Supplementary Figure 3. Polymorphisms in MtrE from *N. meningitidis* and non-invasive *Neisseria* spp.

Red amino acids indicate polymorphisms which are different between the five most common gonococcal MtrE variants and MtrE found in *N. meningitidis* and non-invasive *Neisseria* mapped onto trimeric MtrE (PDB accession number 4MT0); MtrE monomer (blue)

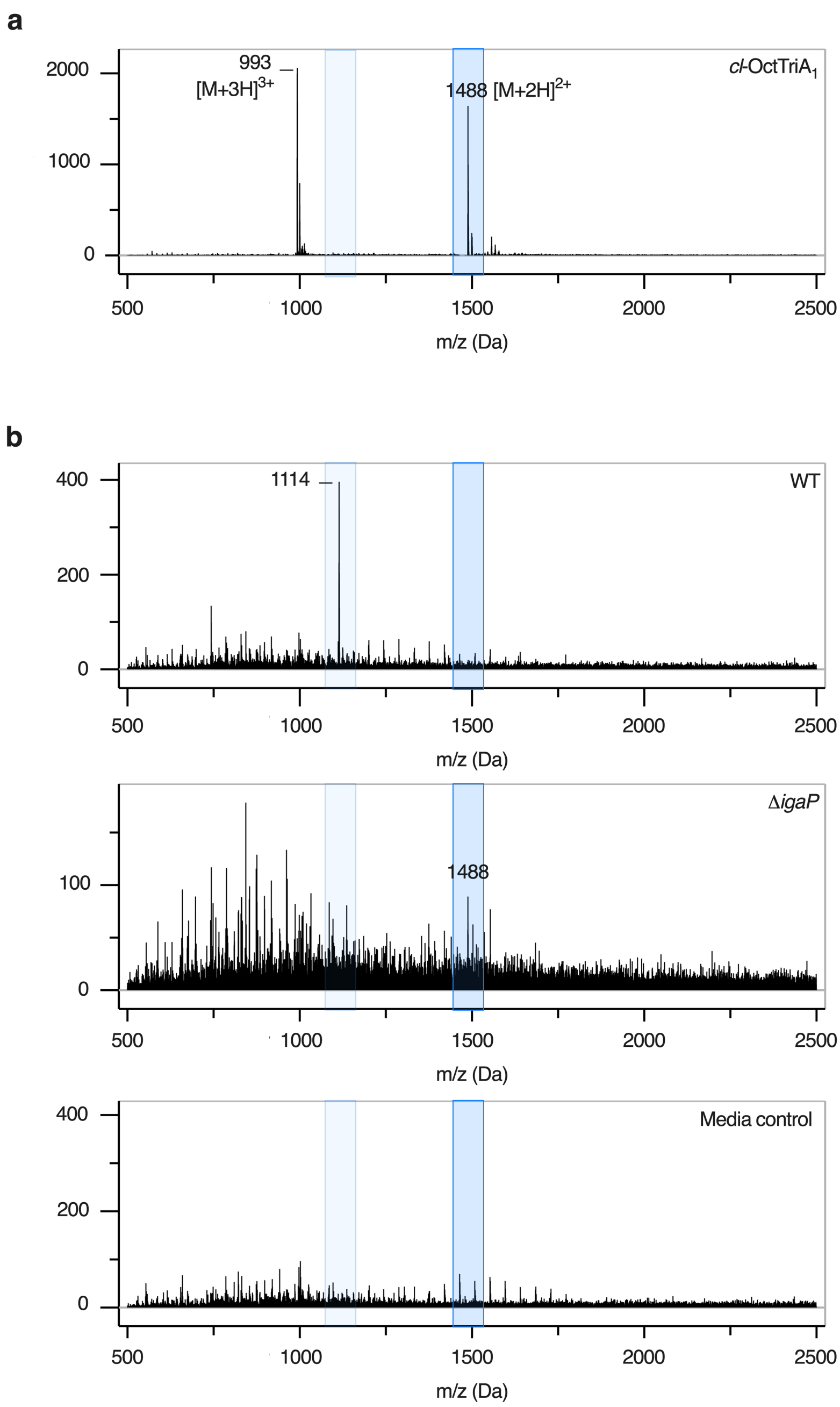

Supplementary Figure 4. Peptide *cl*-OctTriA$_1$ is specifically cleaved by the gonococcal IgAP. (a) Synthesised *cl*-OctTriA$_1$-*c* (dark blue box) in PBS shown as a mass spectrometry control. **(b)** Analysis of *cl*-OctTriA$_1$ after incubation in supernatants from wild-type FA1090 (top) but not FA1090Δ*igaP* (middle) by Electrospray ionisation (ESI) mass spectrometry. MS species corresponding to peptide *cl*-OctTriA$_1$ (dark blue box) and the cleaved product OctTriA$_1$-*c* (light blue box) are indicated in each spectrum. Media only control to indicate background (bottom).

a

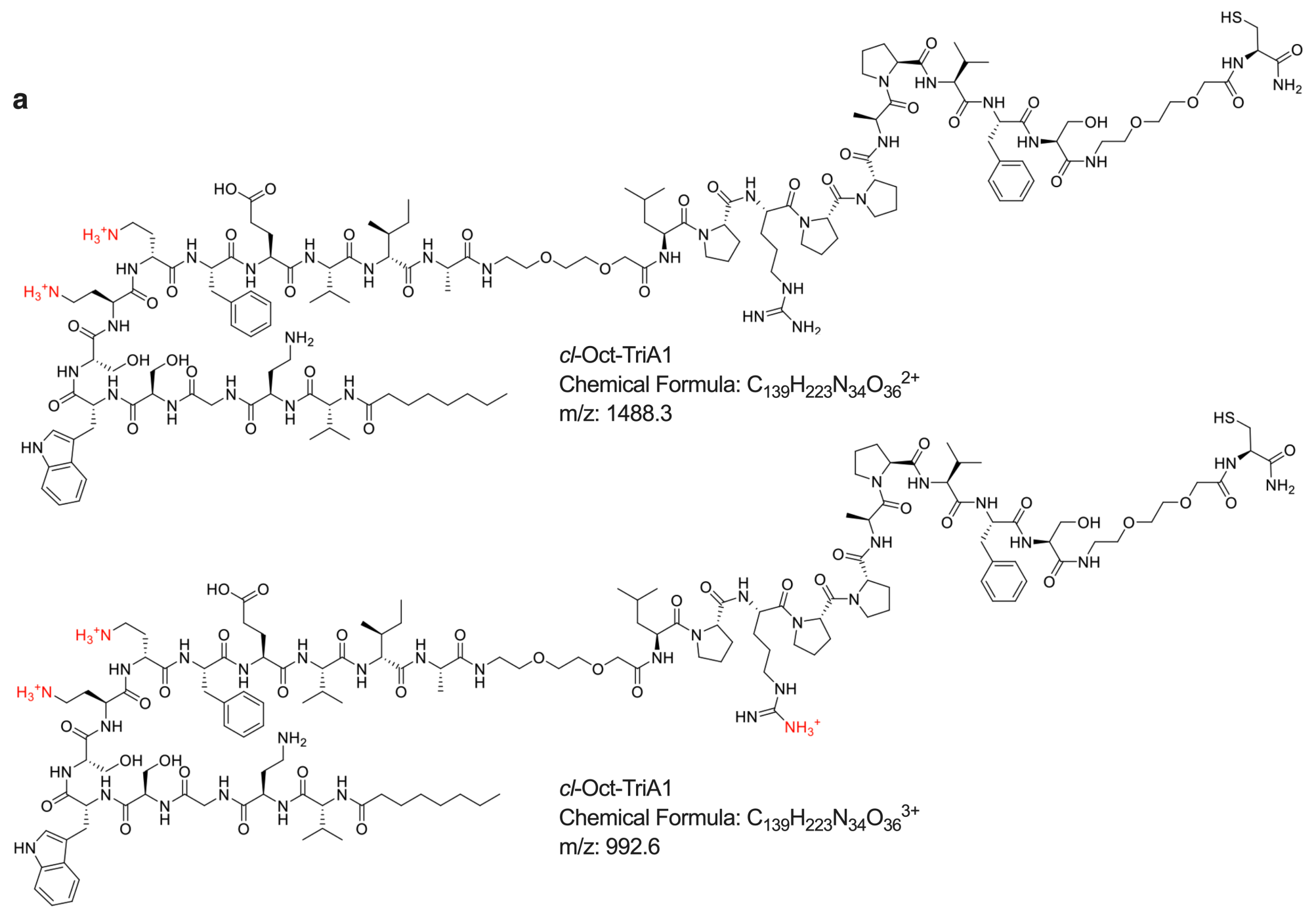

cl-Oct-TriA1
Chemical Formula: $C_{139}H_{223}N_{34}O_{36}^{2+}$
m/z: 1488.3

cl-Oct-TriA1
Chemical Formula: $C_{139}H_{223}N_{34}O_{36}^{3+}$
m/z: 992.6

b

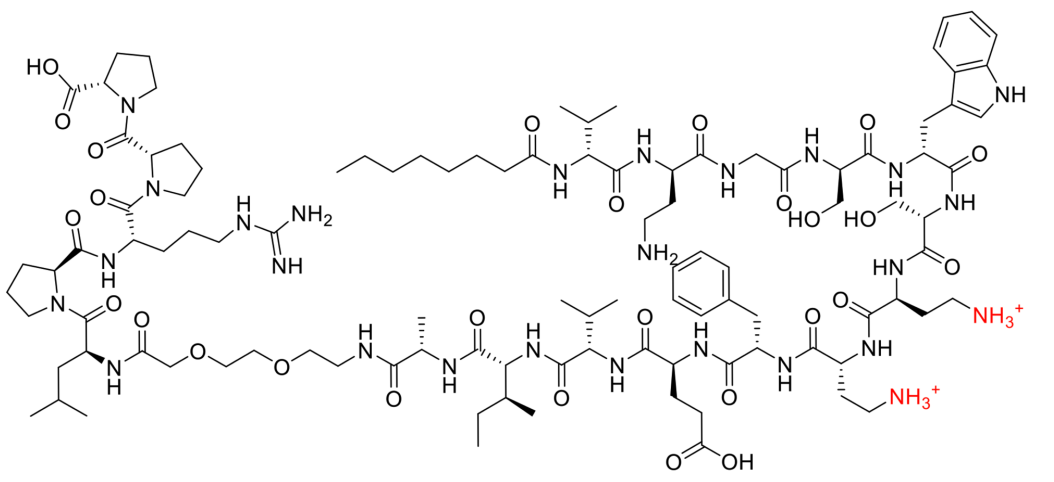

Oct-TriA1-c
Chemical Formula: $C_{105}H_{170}N_{26}O_{27}^{2+}$
m/z: 1114.1

c

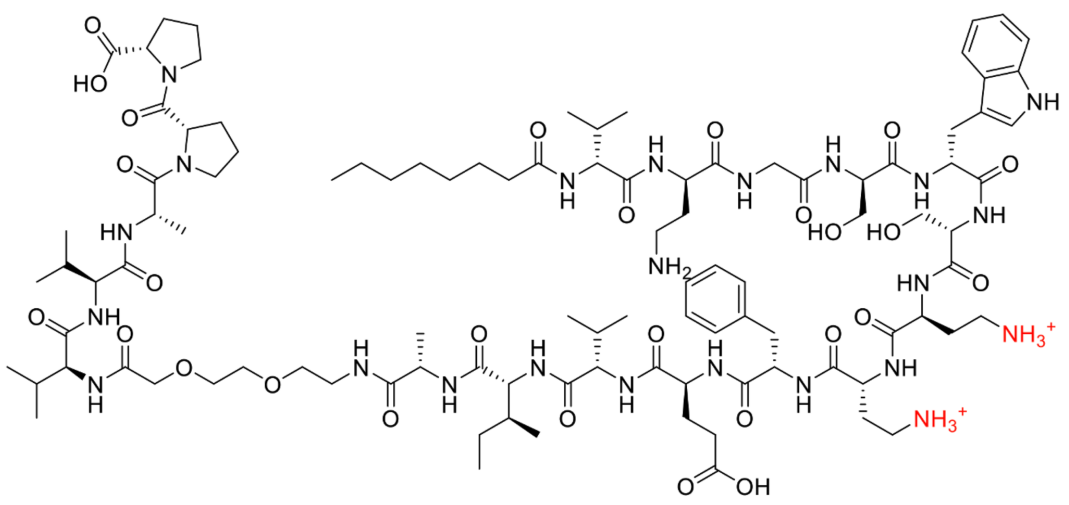

Oct-TriA1-c
Chemical Formula: $C_{101}H_{163}N_{23}O_{27}^{2+}$
m/z: 1065.6

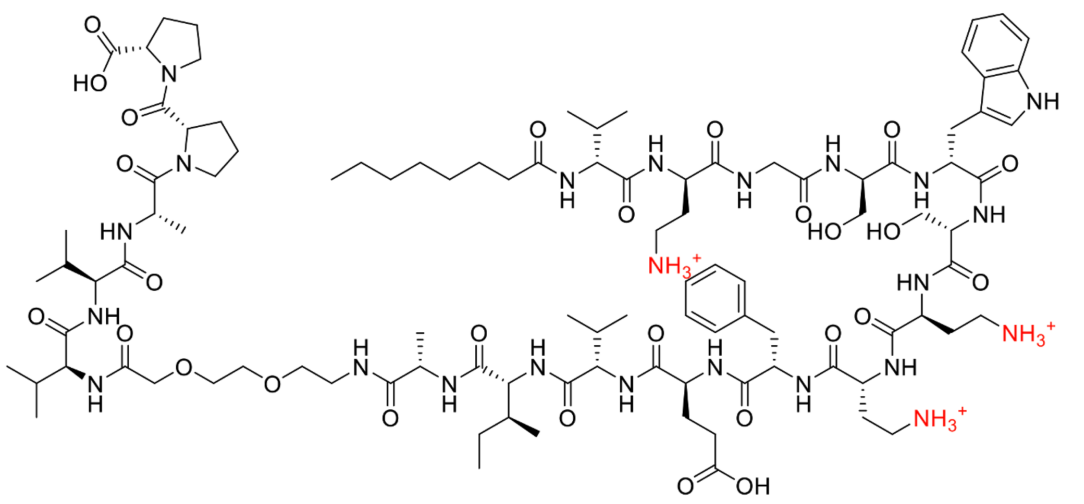

Oct-TriA1-c
Chemical Formula: $C_{101}H_{163}N_{23}O_{27}^{3+}$
m/z: 710.7

Supplementary Figure 5. Chemical structures of MS species identified in cleavage experiments. The species identified in ESI-MS$^+$ with peaks at; **(a)** 1488 (top) and 993 (bottom) m/z correspond to the doubly and triply protonated *cl*-Oct-TriA$_1$ peptide species respectively; at **(b)** 1114 m/z is the doubly protonated Oct-TriA1 -*c* peptide and **(c)** 1066 and 711 correspond to the doubly and triply protonated

peptide species (Octanoyl-DVal-DDab-Gly-DSer-DTrp-Ser-Dab-DDab-Phe-Glu-Val-DalIe-Ala-O2Oc-Val-Val-Ala-Pro-Pro-OH) respectively. Likely protonated residues {red), chemical formula, and the m/z of these species are indicated.

a

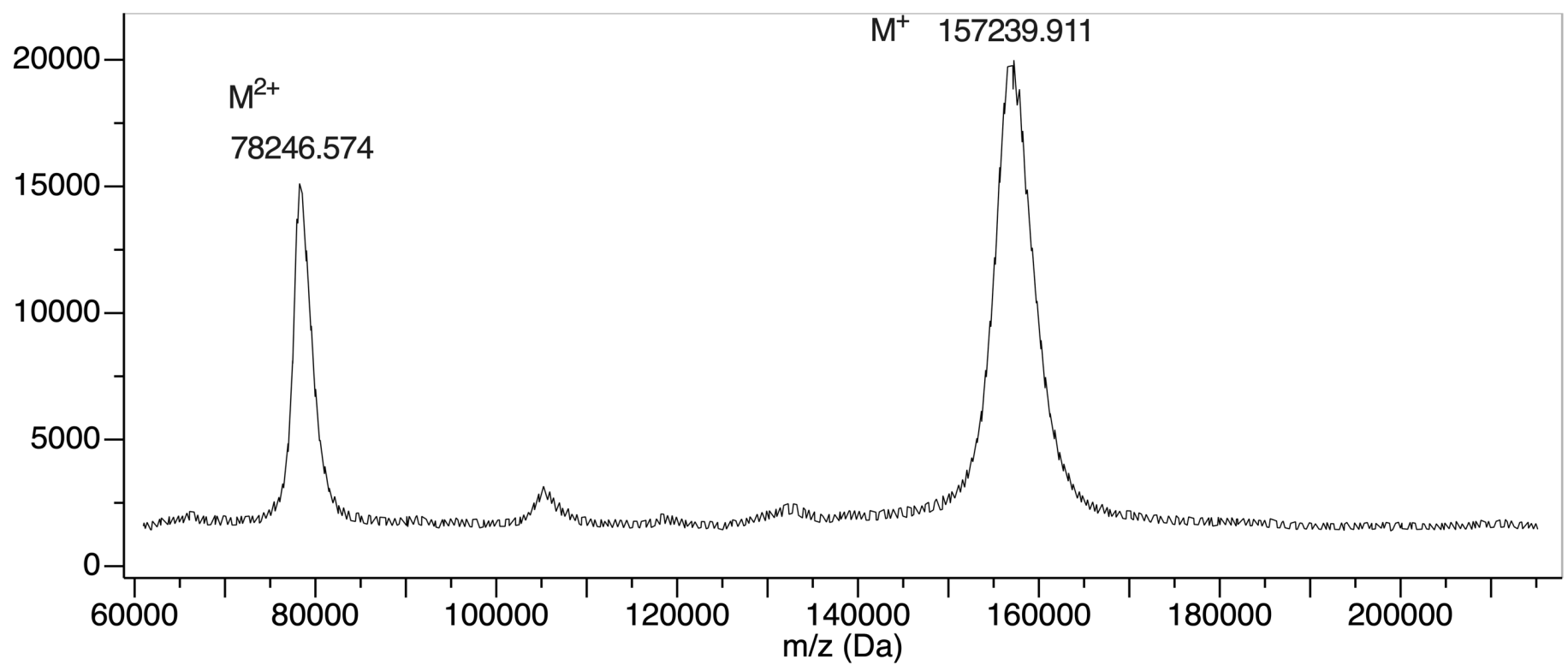

b

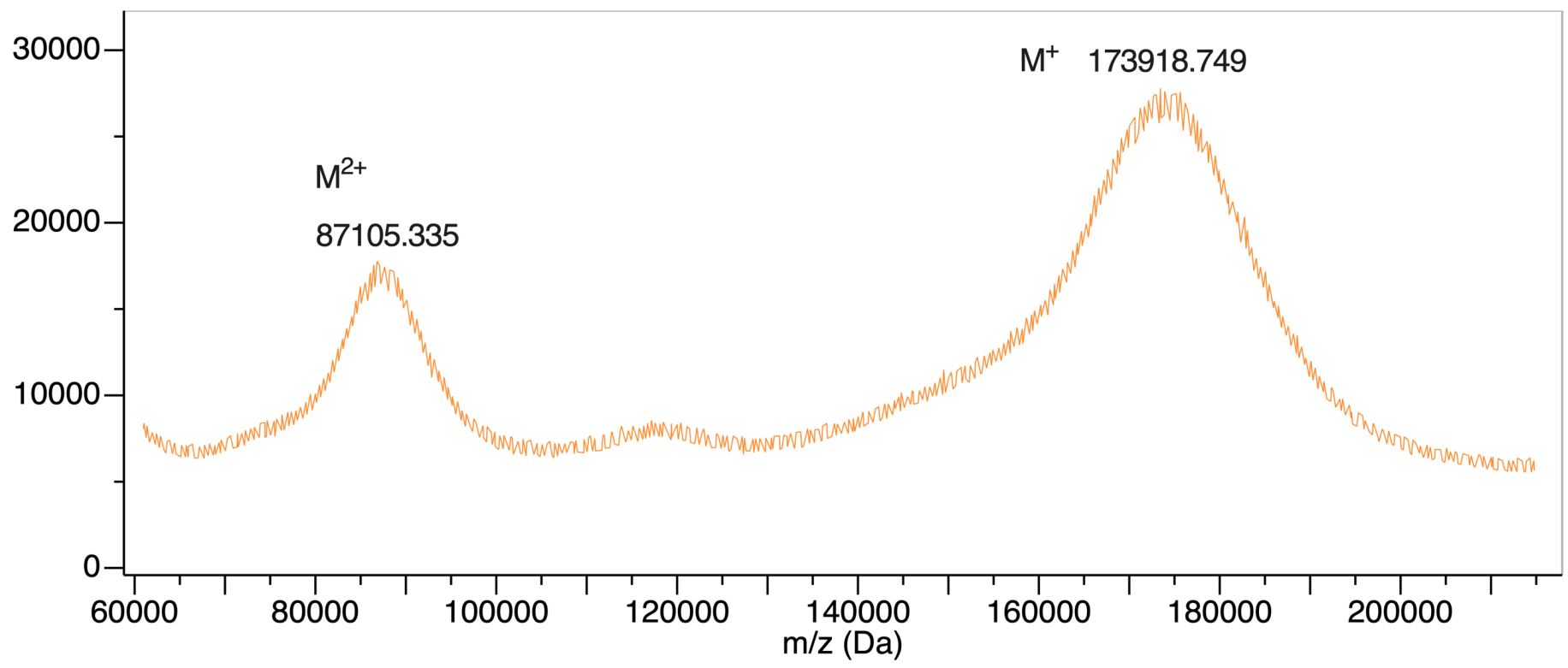

Supplementary Figure 6. Non-cleavable ADCs have a similar DAR to IgAP cleavable ADCs. MALDI-TOF analysis of mNg001 (top, black) and ADC covalently linked to synthesized uncleavable OctTriA$_1$ analogue, uncl-Oct-TriA$_1$ (bottom, orange). Change in mass, compared to mNg001 indicates a DAR of approximately 6 peptides per ADC.

a

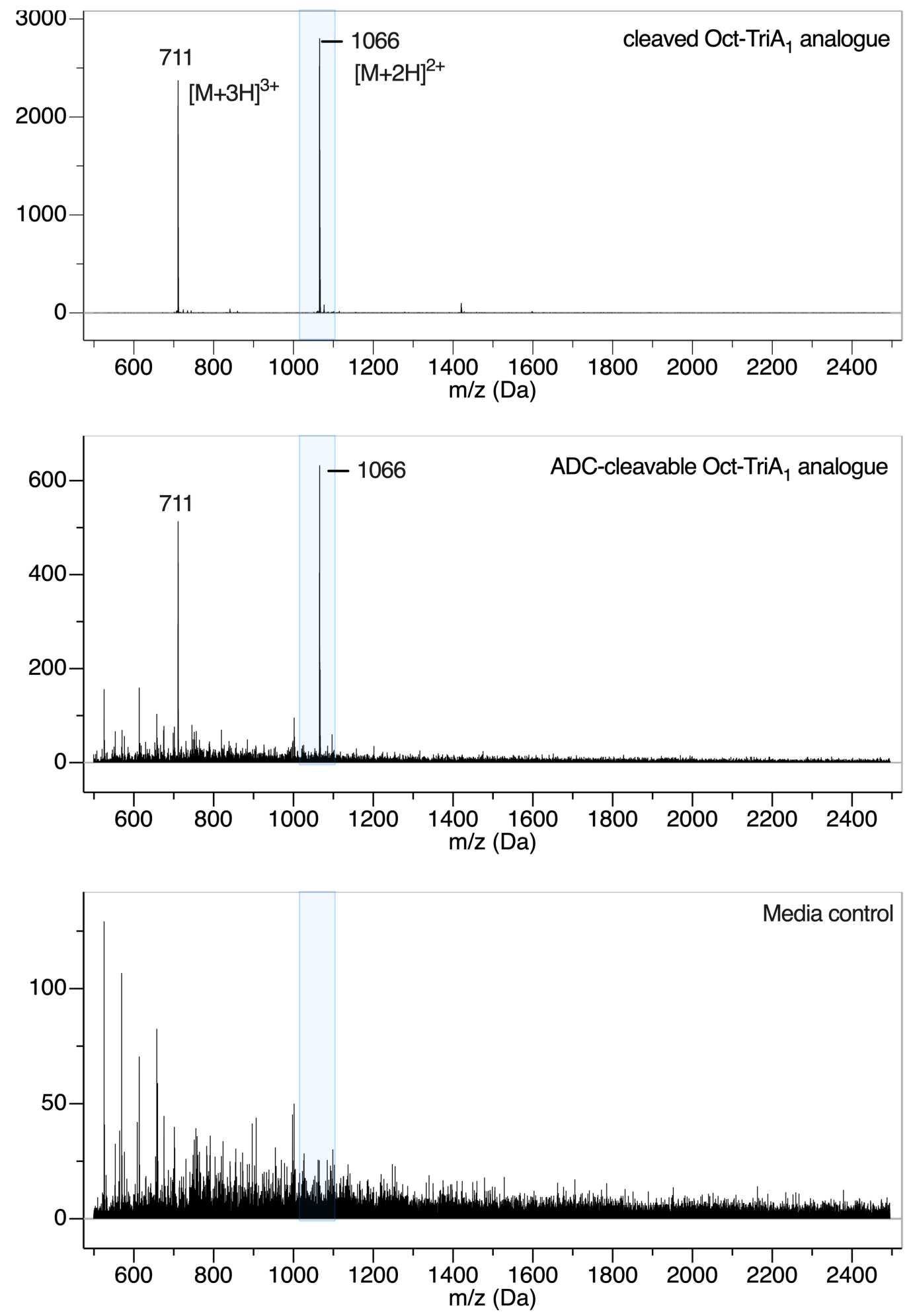

Peptide name	Peptide	Peptide sequence	Molecular weight (Da)
Cleaved Oct-TriA1 analogue	Oct-TriA1-(VVAPP)	Octanoyl-DVal-DDab-Gly-DSer-DTrp-Ser-Dab-DDab-Phe-Glu-Val -Dalle-Ala-O2Oc-Val-Val-Ala-Pro-Pro-OH	2129.7
ADC conjugated cleaveable Oct-TriA1 analogue	Oct-TriA1-(VVAPPSPQAN-C)	Octanoyl-DVal-DDab-Gly-DSer-DTrp-Ser-Dab-DDab-Phe-Glu-Val -Dalle-Ala-O2Oc-Val-Val-Ala-Pro-Pro-Ser-Pro-Gln-Ala-Asn-O2Oc-Cys-NH2	2873.7

b

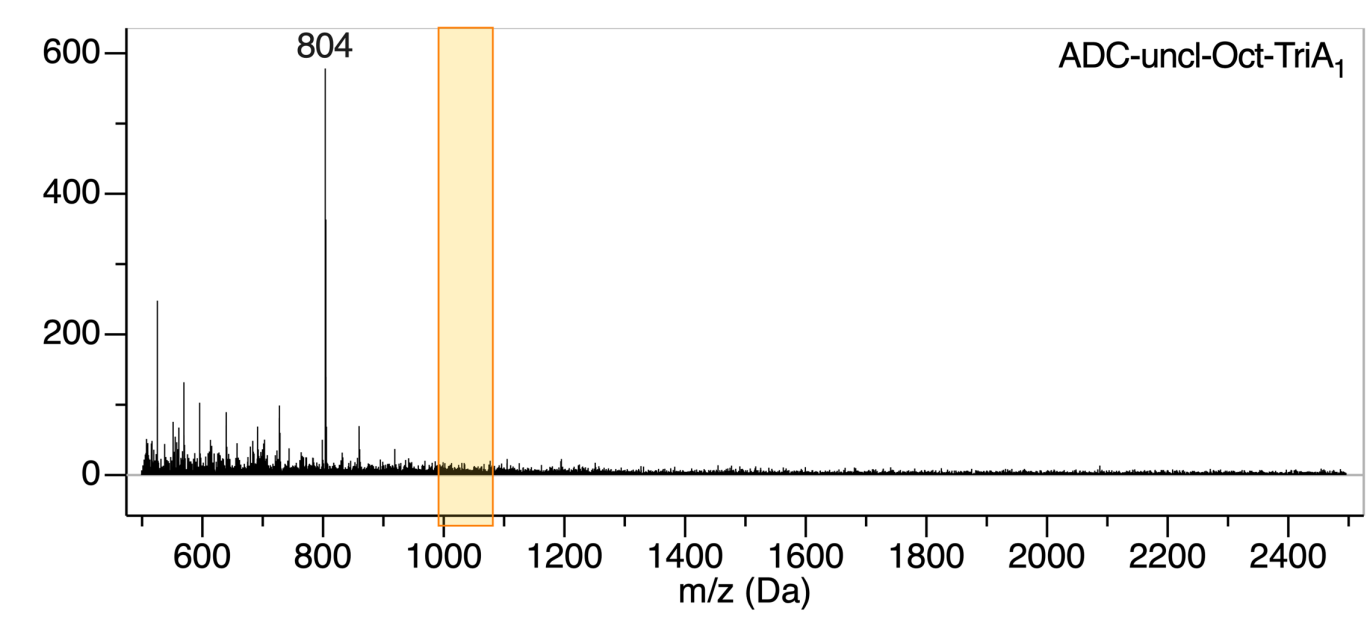

Supplementary Figure 7. Peptide *uncl*-OctTriA$_1$ is not cleaved from the ADC by the gonococcal IgAP. (a) Synthesised cleaved OctTriA$_1$ (cleaved linker sequence Val-Val-Ala-Pro-Pro) (blue box, top) in PBS shown as a mass spectrometry control. Analysis of cleavage of OctTriA$_1$ from ADC after incubation in supernatant from wild-type FA1090 (middle) by Electrospray ionisation (ESI) mass spectrometry. Media only control to indicate background (bottom). (b) No peptide release was observed after incubation of ADC-uncl-OctTriA$_1$ with supernatant from FA1090 (orange box).

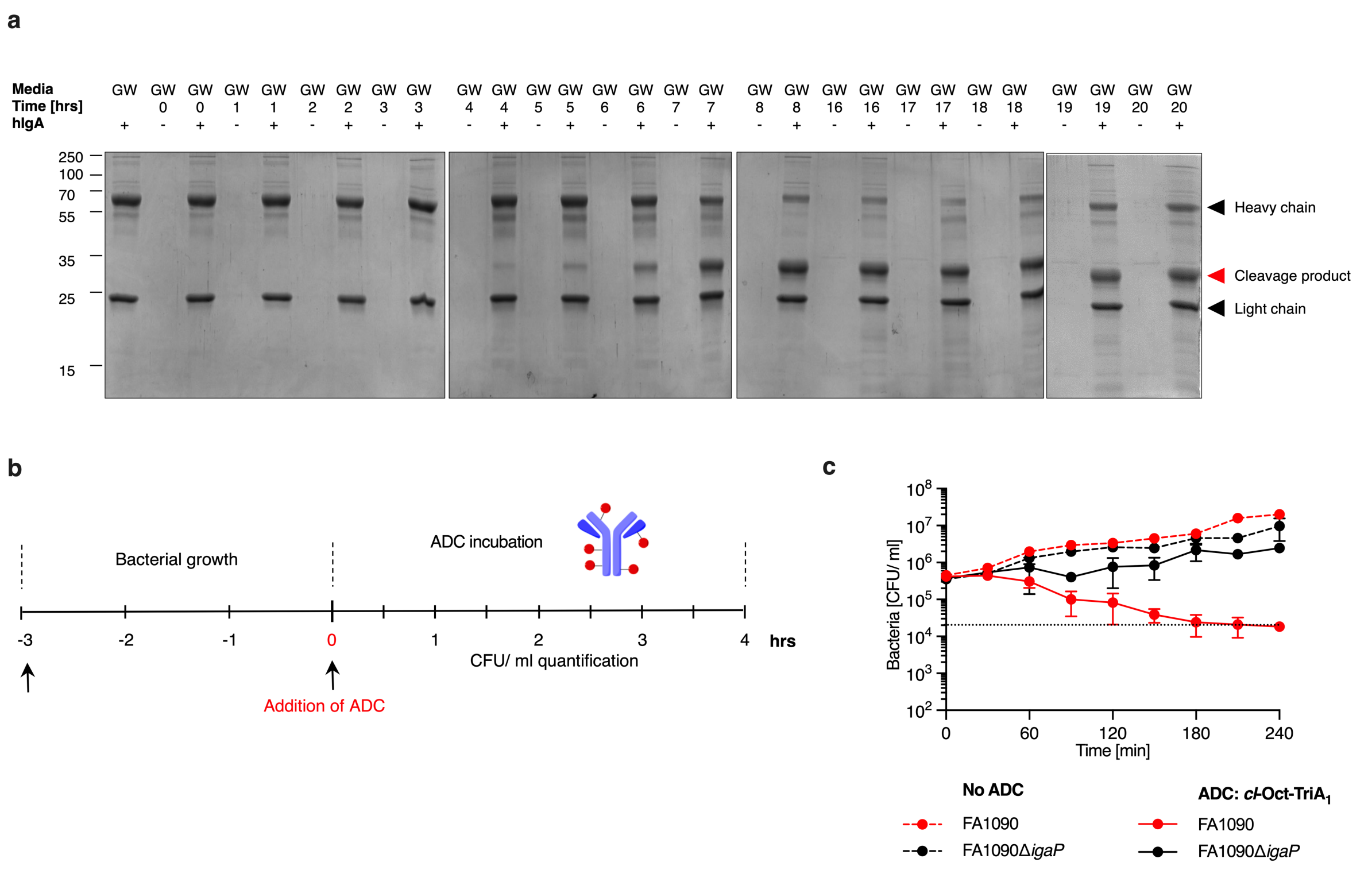

Supplementary Figure 8. Gonococcal IgAP cleaves human IgA and mediates ADC bactericidal activity. (a) 5 µg of human IgA1 was incubated for 4 hours with supernatant from FA1090 growth taken at the indicated timepoints and resolved by SDS-PAGE electrophoresis prior to visualising with Coomassie blue. The heavy and light chains of IgA1 are shown with the heavy chain cleavage products, Fcα and Fabα. **(b)** Time to kill curve analysis with ADCs protocol. *N. gonorrhoeae* were grown for 3 hours prior to the addition of ADC and and CFU/ ml was calculated every 30 minutes for 4 hours. **(c)** Time to kill curve analysis of ADC-cl-OctTriA$_1$ against FA1090 (red), isogenic *ΔIgAP* (black). No ADC (dashed line) and ADC (line). All data shown are the mean ± standard deviation of CFU/ml (n = 2).

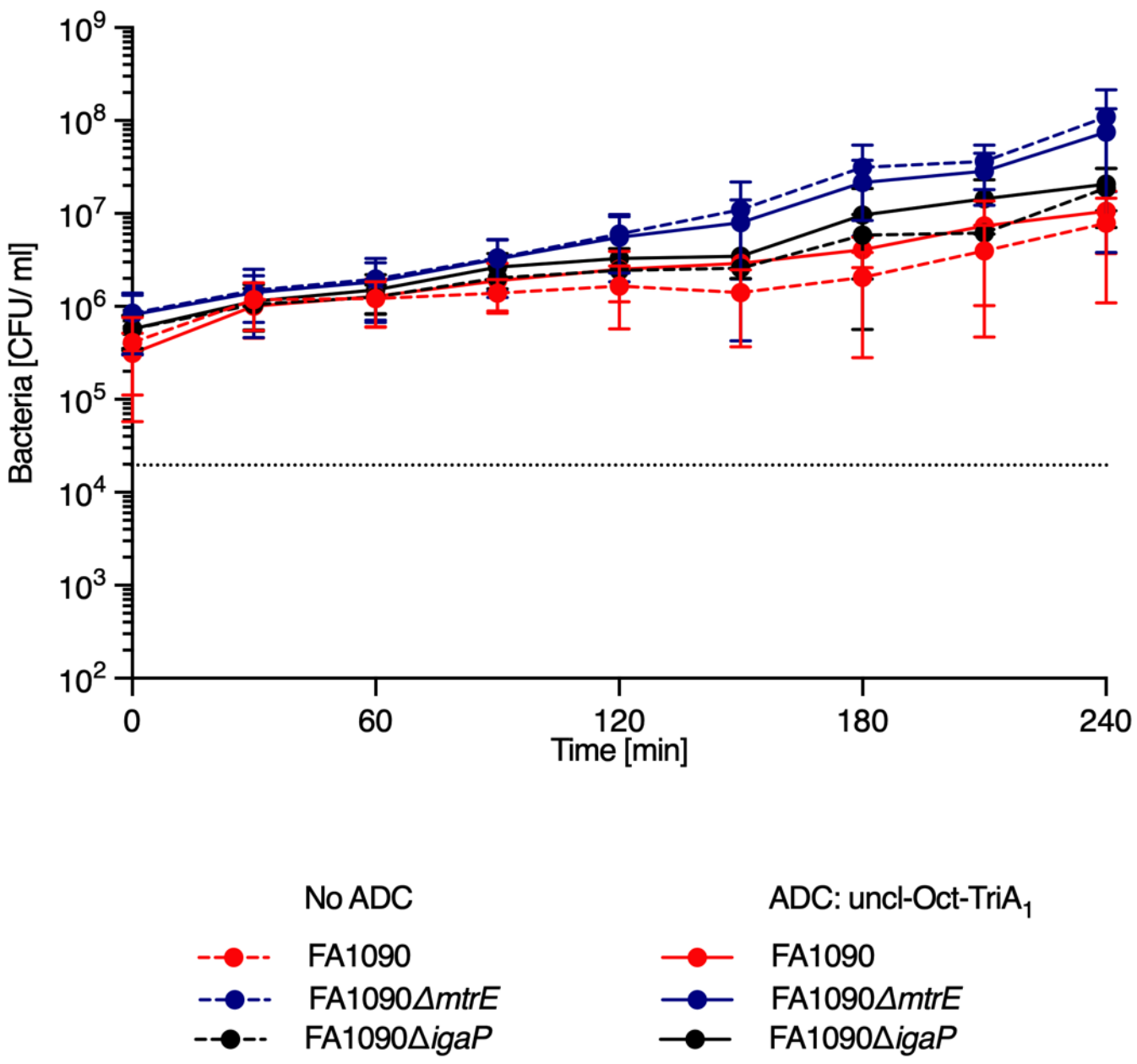

Supplementary Figure 9. Non-cleavable ADCs are not bactericidal in the presence of the gonococcal IgAP.

Time to kill curve analysis of ADC-unc*l*-OctTriA$_1$ against FA1090 (red), isogenic *ΔigAP* (black) and *ΔmtrE* (blue). No ADC (dashed line) and ADC (line). All data shown are the mean ± standard deviation of CFU/ml (n = 3).

Supplementary Table 1. MtrE variants in *N. gonorrhoeae*

MtrE protein variant	MtrE (NEIS1632) alleles with the same a.a. sequence	Number of isolates	Percentage of isolates
1	237, 238, 239, 286, 289, 300, 965, 1244, 1357, 1391, 1413, 1467	5863	31.4623
2	234, 247, 248, 283, 284, 285, 292, 295, 298, 365, 849, 906, 955, 1035, 1205,1206, 1359, 1385, 1387, 1411, 1434, 1452	4043	21.6957
3	232, 240 241,280, 287, 288, 971, 1389, 1453	3833	20.5688
4	235, 243, 293, 439, 919, 960, 967, 969, 1386, 1394, 1426, 1463	2276	12.2136
5	233	1902	10.2066
6	1570	282	1.5133
7	246, 281, 297	120	0.6439
8	1217, 1227, 1356, 1408	22	0.1181
9	302, 1448	21	0.1127
10	593	20	0.1073
11	1073	20	0.1073
12	236, 368,839, 1010, 1409, 1433	18	0.0966
13	1237	17	0.0912
14	279	14	0.0751
15	330	13	0.0698
16	966	11	0.0590
17	818	9	0.0483
18	242	7	0.0376
19	304, 366, 1203	6	0.0322
20	838	6	0.0322
21	1031	6	0.0322
22	296	5	0.0268
23	1086	5	0.0268
24	1172	5	0.0268
25	1314	5	0.0268
26	920	4	0.0215
27	1465	4	0.0215
28	1468	4	0.0215
29	606	3	0.0161
30	610	3	0.0161
31	1084	3	0.0161
32	1208	3	0.0161
33	1435	3	0.0161
34	282	2	0.0107
35	290	2	0.0107
36	303, 1201	2	0.0107
37	592	2	0.0107
38	605	2	0.0107
39	611	2	0.0107
40	649	2	0.0107
41	817, 1245	2	0.0107
42	874	2	0.0107
43	959	2	0.0107
44	968	2	0.0107
45	1198	2	0.0107
46	1358	2	0.0107
47	1390	2	0.0107
48	1392	2	0.0107
49	1424	2	0.0107
50	1425	2	0.0107
51	1619	2	0.0107
52	245	1	0.0054
53	291	1	0.0054
54	294	1	0.0054
55	299	1	0.0054
56	301	1	0.0054
57	329	1	0.0054
58	367	1	0.0054

59	406	1	0.0054
60	428	1	0.0054
61	594	1	0.0054
62	607	1	0.0054
63	608	1	0.0054
64	609	1	0.0054
65	615	1	0.0054
66	715	1	0.0054
67	956	1	0.0054
68	957	1	0.0054
69	961	1	0.0054
70	1173	1	0.0054
71	1197	1	0.0054
72	1199	1	0.0054
73	1200	1	0.0054
74	1202	1	0.0054
75	1204	1	0.0054
76	1207	1	0.0054
77	1209	1	0.0054
78	1218	1	0.0054
79	1228	1	0.0054
80	1229	1	0.0054
81	1230	1	0.0054
82	1250	1	0.0054
83	1360	1	0.0054
84	1361	1	0.0054
85	1362	1	0.0054
86	1384	1	0.0054
87	1388	1	0.0054
88	1393	1	0.0054
89	1395	1	0.0054
90	1410	1	0.0054
91	1412	1	0.0054
92	1464	1	0.0054
93	1466	1	0.0054
94	1577	1	0.0054

Supplementary Table 2. ***N. gonorrhoeae*** **MtrE outer membrane loop sequences**

	Sequence	MtrE alleles	Number of isolates	Percentage of isolates
**Loop 1**	SLSGGNV	All except below	18633	99.9893
	SLIGGNV	290	2	0.0107
**Loop 2**	SVELGGLFKSGTG	All except below	18299	98.1969
	SVELGGLFRSGTG	279, 282, 299	17	0.0912
	SAELGGLFRSGTG	428, 649, 1084, 1172, 1237, 1770	310	1.6635
	SVELGGLFKSDTG	605	2	0.0107
	SAELSGLFRSGTG	615	1	0.0054
	SVELGGLFKSGNG	1031	6	0.0322

Supplementary Table 3. Presence of *N. gonorrhoeae* MtrE (NEIS1632) alleles in *N. meningitidis* and commensal *Neisseria* spp.

MtrE (NEIS1632) alleles	Species	Number of Isolates
233	*Neisseria meningitidis*	1/ 45,938
838	*Neisseria meningitidis*	3/ 45,938
649	*Neisseria meningitidis*	2/ 45,938
649	*Neisseria cinerea*	3/ 61

Supplementary Table 4. *Commensal Neisseria* spp. used in this study for bioinformatic analysis of MtrE and IgAP alleles

PubMLST ID	*Species (Neisseria)*	Isolate	Country	Year	MtrE (NEIS1632) allele	MtrE protein varient	Presence of IgAP (NEIS0651)
1770	*lactamica*	028-12	UK	1997	704		No
1777	*lactamica*	049-12	UK	1998	705		No
1780	*lactamica*	016-24	UK	1998	706		No
1809	*lactamica*	224	UK	2000	707		No
1827	*lactamica*	005-12	UK	1997	75		No
3426	*lactamica*	OX9932380	UK	1999	1872		No
4410	*lactamica*	OX9931639	UK	1999	48		No
4411	*lactamica*	OX9931924	UK	1999	1511		No
4945	*lactamica*	Y92-1009	UK	1992	NA		No
5544	*lactamica*	ATCC 23970	USA	1969	1874		No
8778	*lactamica*	004-12	UK	1997	708		No
8790	*lactamica*	012-12	UK	1997	709		No
8837	*lactamica*	017-02	UK	1997	41		No
8851	*lactamica*	020-06	UK	1997	4		No
8917	*lactamica*	039-03	UK	1997	710		No
26478	*lactamica*	BB97	UK	2012	48		No
26479	*lactamica*	NL-Y92-1009-1	UK	1992	48		No
26531	*lactamica*	NL-Y92-1009-2	UK	1992	48		No
26735	*lactamica*	ERR026908	UK	Unknown	48		No
26736	*lactamica*	ERR026909	UK	Unknown	48		No
26737	*lactamica*	ERR026910	UK	Unknown	48		No
26738	*lactamica*	ERR026911	UK	Unknown	48		No
26739	*lactamica*	ERR026912	UK	Unknown	48		No
26740	*lactamica*	ERR026913	UK	Unknown	48		No
26741	*lactamica*	ERR026914	UK	Unknown	48		No
26742	*lactamica*	ERR026915	UK	Unknown	48		No
26743	*lactamica*	ERR026916	UK	Unknown	48		No
26744	*lactamica*	ERR026917	UK	Unknown	48		No
26745	*lactamica*	ERR026919	UK	Unknown	48		No
26746	*lactamica*	ERR026920	UK	Unknown	48		No
26747	*lactamica*	ERR026921	UK	Unknown	48		No
26748	*lactamica*	ERR026922	UK	Unknown	48		No
26749	*lactamica*	ERR026923	UK	Unknown	48		No
26750	*lactamica*	ERR026924	UK	Unknown	48		No
26751	*lactamica*	ERR026925	UK	Unknown	48		No
26752	*lactamica*	ERR026926	UK	Unknown	48		No
26753	*lactamica*	ERR026927	UK	Unknown	48		No
26754	*lactamica*	ERR026928	UK	Unknown	48		No
26755	*lactamica*	ERR028518	UK	Unknown	48		No
26756	*lactamica*	ERR028519	UK	Unknown	48		No
26757	*lactamica*	ERR028520	UK	Unknown	48		No
26758	*lactamica*	ERR028521	UK	Unknown	48		No
26759	*lactamica*	ERR028522	UK	Unknown	48		No
26760	*lactamica*	ERR028523	UK	Unknown	48		No
26761	*lactamica*	ERR028524	UK	Unknown	48		No
26762	*lactamica*	ERR028525	UK	Unknown	48		No
26763	*lactamica*	ERR028526	UK	Unknown	48		No

26764	*lactamica*	ERR028527	UK	Unknown	48	No
26765	*lactamica*	ERR028528	UK	Unknown	48	No
26766	*lactamica*	ERR028530	UK	Unknown	48	No
26767	*lactamica*	ERR028531	UK	Unknown	48	No
26768	*lactamica*	ERR028532	UK	Unknown	48	No
26769	*lactamica*	ERR028533	UK	Unknown	48	No
26770	*lactamica*	ERR028534	UK	Unknown	48	No
26771	*lactamica*	ERR028537	UK	Unknown	48	No
26772	*lactamica*	ERR028538	UK	Unknown	48	No
26773	*lactamica*	ERR028539	UK	Unknown	48	No
26774	*lactamica*	ERR028540	UK	Unknown	48	No
26775	*lactamica*	ERR028542	UK	Unknown	48	No
26776	*lactamica*	ERR028543	UK	Unknown	48	No
26777	*lactamica*	ERR028544	UK	Unknown	48	No
26778	*lactamica*	ERR028545	UK	Unknown	48	No
26779	*lactamica*	ERR028546	UK	Unknown	48	No
26780	*lactamica*	ERR028547	UK	Unknown	48	No
26781	*lactamica*	ERR028548	UK	Unknown	48	No
26782	*lactamica*	ERR028549	UK	Unknown	48	No
26783	*lactamica*	ERR028550	UK	Unknown	48	No
26784	*lactamica*	ERR028551	UK	Unknown	48	No
26785	*lactamica*	ERR028552	UK	Unknown	48	No
26786	*lactamica*	ERR028554	UK	Unknown	48	No
26787	*lactamica*	ERR028555	UK	Unknown	48	No
26788	*lactamica*	ERR028556	UK	Unknown	48	No
26789	*lactamica*	ERR028557	UK	Unknown	48	No
26790	*lactamica*	ERR028558	UK	Unknown	48	No
26791	*lactamica*	ERR028559	UK	Unknown	48	No
26792	*lactamica*	ERR028560	UK	Unknown	48	No
26793	*lactamica*	ERR028561	UK	Unknown	48	No
26794	*lactamica*	ERR028562	UK	Unknown	48	No
26795	*lactamica*	ERR028563	UK	Unknown	48	No
26796	*lactamica*	ERR028564	UK	Unknown	48	No
26797	*lactamica*	ERR028566	UK	Unknown	48	No
26798	*lactamica*	ERR028567	UK	Unknown	48	No
26799	*lactamica*	ERR028568	UK	Unknown	48	No
26800	*lactamica*	ERR028569	UK	Unknown	48	No
26801	*lactamica*	ERR028570	UK	Unknown	48	No
26802	*lactamica*	ERR028571	UK	Unknown	48	No
26803	*lactamica*	ERR028572	UK	Unknown	48	No
26804	*lactamica*	ERR028573	UK	Unknown	48	No
26805	*lactamica*	ERR028574	UK	Unknown	48	No
26806	*lactamica*	ERR028575	UK	Unknown	48	No
26807	*lactamica*	ERR028535	UK	Unknown	48	No
26808	*lactamica*	ERR028536	UK	Unknown	48	No
26809	*lactamica*	WCB1	UK	Unknown	48	No
26810	*lactamica*	WCB2	UK	Unknown	48	No
26811	*lactamica*	WCB3	UK	Unknown	48	No
26812	*lactamica*	WCB4	UK	Unknown	48	No
26813	*lactamica*	WCB5	UK	Unknown	48	No
26814	*lactamica*	WCB6	UK	Unknown	48	No
26815	*lactamica*	WCB7	UK	Unknown	48	No

26816	*lactamica*	WCB8	UK	Unknown	48	No
26817	*lactamica*	WCB9	UK	Unknown	48	No
26818	*lactamica*	WCB10	UK	Unknown	48	No
27000	*lactamica*	90142v4	UK	Unknown	711	No
27001	*lactamica*	90142v5	UK	Unknown	711	No
29271	*lactamica*	BM35a	Greece	1996	195	No
29272	*lactamica*	BM35b	Greece	1996	195	No
29274	*lactamica*	BM68a	Greece	1997	1884	No
30770	*lactamica*	M45.1	Ireland	2013	250	No
30771	*lactamica*	M46.2	Ireland	2013	250	No
36172	*lactamica*	M10.1	Ireland	2013	358	No
36173	*lactamica*	M11.1	Ireland	2013	358	No
36174	*lactamica*	M15.1	Ireland	2013	754	No
36175	*lactamica*	M17.1	Ireland	2013	250	No
36176	*lactamica*	M18.1	Ireland	2013	250	No
36177	*lactamica*	M21.1	Ireland	2013	250	No
36178	*lactamica*	M22.1	Ireland	2013	250	No
36179	*lactamica*	M23.1	Ireland	2013	250	No
36180	*lactamica*	M24.1	Ireland	2013	250	No
36181	*lactamica*	M26.1	Ireland	2013	250	No
36182	*lactamica*	M27.1	Ireland	2013	250	No
36183	*lactamica*	M28.1	Ireland	2013	250	No
36184	*lactamica*	M33.1	Ireland	2013	250	No
36185	*lactamica*	M4.1	Ireland	2013	250	No
36186	*lactamica*	M43.1	Ireland	2013	250	No
36189	*lactamica*	M5.1	Ireland	2013	250	No
36190	*lactamica*	M7.1	Ireland	2013	250	No
36854	*lactamica*	Q68	UK	2015	706	No
36855	*lactamica*	Q36	UK	2015	363	No
36856	*lactamica*	S291	UK	2015	364	No
36857	*lactamica*	S68	UK	2015	706	No
36858	*lactamica*	Q118	UK	2015	736	No
36859	*lactamica*	S104	UK	2015	48	No
36860	*lactamica*	S258	UK	2015	48	No
36861	*lactamica*	S300	UK	2015	48	No
36862	*lactamica*	S36	UK	2015	363	No
36863	*lactamica*	S201	UK	2015	48	No
36864	*lactamica*	S244	UK	2015	48	No
36865	*lactamica*	S290	UK	2015	48	No
36866	*lactamica*	S114	UK	2015	48	No
36867	*lactamica*	S207	UK	2015	48	No
36868	*lactamica*	S108	UK	2015	48	No
36869	*lactamica*	U291	UK	2015	364	No
36870	*lactamica*	S190	UK	2015	48	No
36871	*lactamica*	U68	UK	2015	48	No
36872	*lactamica*	S118	UK	2015	706	No
36873	*lactamica*	S279	UK	2015	48	No
36874	*lactamica*	U36	UK	2015	363	No
36875	*lactamica*	S259	UK	2015	48	No
36876	*lactamica*	S82	UK	2015	48	No
36877	*lactamica*	S166	UK	2015	48	No
36878	*lactamica*	S181	UK	2015	48	No

36879	*lactamica*	S284	UK	2015	48	No
36880	*lactamica*	Q139	UK	2015	1891	No
36881	*lactamica*	S264	UK	2015	48	No
36882	*lactamica*	S221	UK	2015	48	No
36883	*lactamica*	S64	UK	2015	48	No
36884	*lactamica*	S63	UK	2015	48	No
36885	*lactamica*	S113	UK	2015	48	No
36886	*lactamica*	S289	UK	2015	48	No
36887	*lactamica*	S260	UK	2015	48	No
36888	*lactamica*	W68	UK	2015	706	No
36889	*lactamica*	W258	UK	2015	48	No
36890	*lactamica*	W207	UK	2015	48	No
36891	*lactamica*	W114	UK	2015	48	No
36892	*lactamica*	S293	UK	2015	48	No
36893	*lactamica*	S315	UK	2015	48	No
36894	*lactamica*	S112	UK	2015	48	No
36895	*lactamica*	S170	UK	2015	48	No
36896	*lactamica*	S313	UK	2015	48	No
36897	*lactamica*	W118	UK	2015	736	No
36898	*lactamica*	W72	UK	2015	48	No
36899	*lactamica*	S158	UK	2015	48	No
36900	*lactamica*	S303	UK	2015	48	No
36901	*lactamica*	S139	UK	2015	1891	No
36902	*lactamica*	W88	UK	2015	48	No
36903	*lactamica*	S253	UK	2015	48	No
36904	*lactamica*	W290	UK	2015	48	No
36905	*lactamica*	W201	UK	2015	48	No
36906	*lactamica*	S255	UK	2015	48	No
36907	*lactamica*	W190	UK	2015	48	No
36908	*lactamica*	W36	UK	2015	363	No
36909	*lactamica*	S227	UK	2015	48	No
36910	*lactamica*	S222	UK	2015	48	No
36911	*lactamica*	W279	UK	2015	48	No
36912	*lactamica*	U160	UK	2015	48	No
36913	*lactamica*	W38	UK	2015	48	No
36914	*lactamica*	W172	UK	2015	48	No
36915	*lactamica*	W181	UK	2015	48	No
36916	*lactamica*	W82	UK	2015	48	No
36917	*lactamica*	U101	UK	2015	48	No
36918	*lactamica*	U255	UK	2015	48	No
36919	*lactamica*	U170	UK	2015	48	No
36920	*lactamica*	U227	UK	2015	48	No
36921	*lactamica*	W64	UK	2015	48	No
36922	*lactamica*	W264	UK	2015	48	No
36923	*lactamica*	W77	UK	2015	48	No
36924	*lactamica*	W113	UK	2015	48	No
36925	*lactamica*	W260	UK	2015	48	No
36926	*lactamica*	W63	UK	2015	48	No
36927	*lactamica*	W139	UK	2015	1891	No
36928	*lactamica*	W158	UK	2015	48	No
36929	*lactamica*	W170	UK	2015	48	No
36930	*lactamica*	W227	UK	2015	48	No

36931	*lactamica*	W309	UK	2015	48	No
36932	*lactamica*	W312	UK	2015	48	No
36933	*lactamica*	W303	UK	2015	48	No
36934	*lactamica*	W59	UK	2015	1892	No
36935	*lactamica*	W93	UK	2015	48	No
36936	*lactamica*	W222	UK	2015	48	No
36937	*lactamica*	Y315	UK	2015	48	No
36938	*lactamica*	Y291	UK	2015	364	No
36939	*lactamica*	W293	UK	2015	48	No
36940	*lactamica*	W284	UK	2015	48	No
36941	*lactamica*	Y688	UK	2015	706	No
36942	*lactamica*	Y104	UK	2015	48	No
36943	*lactamica*	W253	UK	2015	48	No
36944	*lactamica*	Y88	UK	2015	48	No
36945	*lactamica*	Y72	UK	2015	48	No
36946	*lactamica*	Y36	UK	2015	363	No
36947	*lactamica*	Y244	UK	2015	48	No
36948	*lactamica*	Y290	UK	2015	48	No
36949	*lactamica*	Y201	UK	2015	48	No
36950	*lactamica*	Y190	UK	2015	48	No
36951	*lactamica*	Y264	UK	2015	48	No
36952	*lactamica*	Y172	UK	2015	48	No
36953	*lactamica*	Y38	UK	2015	48	No
36954	*lactamica*	Y221	UK	2015	48	No
36955	*lactamica*	Y64	UK	2015	48	No
36956	*lactamica*	Y316	UK	2015	48	No
36957	*lactamica*	Y93	UK	2015	48	No
36958	*lactamica*	Y227	UK	2015	48	No
36959	*lactamica*	Y313	UK	2015	48	No
36960	*lactamica*	Y253	UK	2015	48	No
36961	*lactamica*	Y312	UK	2015	48	No
36962	*lactamica*	Y170	UK	2015	48	No
36963	*lactamica*	Y77	UK	2015	48	No
36964	*lactamica*	Y113	UK	2015	48	No
36965	*lactamica*	AA72	UK	2015	48	No
36966	*lactamica*	AA290	UK	2015	48	No
36967	*lactamica*	AA172	UK	2015	48	No
36968	*lactamica*	CC68	UK	2015	706	No
36969	*lactamica*	AA36	UK	2015	363	No
36970	*lactamica*	AA201	UK	2015	48	No
36971	*lactamica*	AA82	UK	2015	48	No
36972	*lactamica*	AA118	UK	2015	48	No
36973	*lactamica*	CC290	UK	2015	48	No
36974	*lactamica*	CC287	UK	2015	48	No
36975	*lactamica*	CC298	UK	2015	48	No
36976	*lactamica*	AA63	UK	2015	48	No
36977	*lactamica*	AA244	UK	2015	48	No
36978	*lactamica*	CC291	UK	2015	NA	No
36979	*lactamica*	AA121	UK	2015	1893	No
36980	*lactamica*	CC21	UK	2015	727	No
36981	*lactamica*	AA221	UK	2015	48	No
36982	*lactamica*	AA64	UK	2015	48	No

36983	*lactamica*	CC201	UK	2015	48	No
36984	*lactamica*	AA181	UK	2015	48	No
36985	*lactamica*	AA160	UK	2015	48	No
36986	*lactamica*	AA158	UK	2015	48	No
36987	*lactamica*	AA93	UK	2015	48	No
36988	*lactamica*	CC38	UK	2015	48	No
36989	*lactamica*	CC172	UK	2015	48	No
36990	*lactamica*	AA101	UK	2015	48	No
36991	*lactamica*	AA260	UK	2015	48	No
36992	*lactamica*	CC121	UK	2015	1893	No
36993	*lactamica*	AA227	UK	2015	48	No
36994	*lactamica*	AA166	UK	2015	48	No
36995	*lactamica*	AA113	UK	2015	48	No
36996	*lactamica*	AA139	UK	2015	1891	No
36997	*lactamica*	AA170	UK	2015	48	No
36998	*lactamica*	AA313	UK	2015	48	No
36999	*lactamica*	CC63	UK	2015	48	No
37000	*lactamica*	CC213	UK	2015	48	No
37001	*lactamica*	CC279	UK	2015	48	No
37002	*lactamica*	CC160	UK	2015	48	No
37003	*lactamica*	CC166	UK	2015	48	No
37004	*lactamica*	CC313	UK	2015	48	No
37005	*lactamica*	CC260	UK	2015	48	No
37006	*lactamica*	CC227	UK	2015	48	No
37007	*lactamica*	CC77	UK	2015	48	No
37008	*lactamica*	CC315	UK	2015	48	No
37009	*lactamica*	CC113	UK	2015	48	No
37010	*lactamica*	CC170	UK	2015	48	No
37011	*lactamica*	CC309	UK	2015	48	No
37012	*lactamica*	CC182	UK	2015	48	No
37013	*lactamica*	DZER0	UK	2015	48	No
37014	*lactamica*	CC295	UK	2015	48	No
37015	*lactamica*	CC118	UK	2015	48	No
37016	*lactamica*	S160	UK	2015	48	No
37017	*lactamica*	S213	UK	2015	48	No
37018	*lactamica*	Y260	UK	2015	48	No
37261	*lactamica*	07AZI_T_009	USA	Unknown	1894	No
38175	*lactamica*	M37084	Brazil	2014	1895	No
38390	*lactamica*	TRI-648	Greece	2014	1896	No
38975	*lactamica*	LNP17075	France	Unknown	1900	No
38985	*lactamica*	LNP22985	France	Unknown	751	No
42198	*lactamica*	LNP28499	France	2016	456	No
42495	*lactamica*	M16 240028	UK	2016	714	No
43546	*lactamica*	BR40087	UK	2014	724	No
43547	*lactamica*	BR40408	UK	2014	706	No
43549	*lactamica*	BR41153	UK	2014	725	No
43555	*lactamica*	BR41212	UK	2014	727	No
43557	*lactamica*	BR41222	UK	2014	728	No
43562	*lactamica*	BR41283	UK	2014	727	No
43563	*lactamica*	BR41284	UK	2015	729	No
43574	*lactamica*	BR40191	UK	2014	730	No
43581	*lactamica*	BR40282	UK	2014	195	No

43585	*lactamica*	BR40313	UK	2014	4	No
43592	*lactamica*	BR40406	UK	2014	731	No
43594	*lactamica*	BR40429	UK	2014	4	No
43617	*lactamica*	BR41350	UK	2015	514	No
43618	*lactamica*	BR41361	UK	2015	733	No
43619	*lactamica*	BR41362	UK	2015	480	No
43620	*lactamica*	BR41372	UK	2015	734	No
43628	*lactamica*	BR41427	UK	2015	4	No
43629	*lactamica*	BR41436	UK	2015	515	No
43647	*lactamica*	BR41506	UK	2015	725	No
43657	*lactamica*	BR41632	UK	2015	735	No
43659	*lactamica*	BR41660	UK	2015	516	No
43664	*lactamica*	BR41700	UK	2015	96	No
43665	*lactamica*	BR41703	UK	2015	725	No
43670	*lactamica*	BR41776	UK	2015	736	No
43673	*lactamica*	BR41783	UK	2015	456	No
43675	*lactamica*	BR41788	UK	2015	4	No
43678	*lactamica*	BR41803	UK	2015	517	No
43736	*lactamica*	BR40507	UK	2014	706	No
43773	*lactamica*	BR40790	UK	2014	519	No
43775	*lactamica*	BR40811	UK	2014	724	No
43780	*lactamica*	BR40976	UK	2014	520	No
43782	*lactamica*	BR40977	UK	2014	520	No
43786	*lactamica*	BR41030	UK	2014	475	No
43787	*lactamica*	BR41033	UK	2014	476	No
43788	*lactamica*	BR41072	UK	2014	709	No
43791	*lactamica*	BR41110	UK	2014	737	No
43798	*lactamica*	BR41799	UK	2015	48	No
44075	*lactamica*	ST41846	UK	2015	731	No
44077	*lactamica*	ST41828	UK	2015	738	No
44098	*lactamica*	ST41711	UK	2015	48	No
44105	*lactamica*	ST41658	UK	2015	733	No
44109	*lactamica*	BR40616	UK	2014	4	No
44132	*lactamica*	ST41390	UK	2015	525	No
44135	*lactamica*	ST41374	UK	2015	48	No
44149	*lactamica*	ST41540	UK	2015	526	No
44175	*lactamica*	ST41190	UK	2015	4	No
44177	*lactamica*	ST41350	UK	2015	795	No
44180	*lactamica*	ST41302	UK	2015	4	No
44218	*lactamica*	GL41050	UK	2014	529	No
44243	*lactamica*	ST41937	UK	2015	723	No
44254	*lactamica*	ST42213	UK	2015	725	No
44263	*lactamica*	ST42011	UK	2015	727	No
44268	*lactamica*	ST42026	UK	2015	530	No
44351	*lactamica*	ST40632	UK	2015	707	No
44352	*lactamica*	ST40619	UK	2015	725	No
44354	*lactamica*	ST40592	UK	2015	740	No
44358	*lactamica*	ST40406	UK	2015	48	No
44359	*lactamica*	ST40535	UK	2015	741	No
44360	*lactamica*	ST40688	UK	2015	707	No
44374	*lactamica*	ST40873	UK	2015	727	No
44384	*lactamica*	ST40746	UK	2015	537	No

44385	*lactamica*	ST40741	UK	2015	538	No
44413	*lactamica*	ST40345	UK	2015	742	No
44421	*lactamica*	ST40261	UK	2015	530	No
44448	*lactamica*	OX40115	UK	2014	533	No
44452	*lactamica*	OX40143	UK	2014	472	No
44453	*lactamica*	OX40150	UK	2014	744	No
44455	*lactamica*	OX40156	UK	2014	745	No
44459	*lactamica*	OX40170	UK	2014	472	No
44472	*lactamica*	OX40301	UK	2014	476	No
44473	*lactamica*	OX40308	UK	2014	746	No
44480	*lactamica*	OX40430	UK	2015	472	No
44481	*lactamica*	OX40433	UK	2015	472	No
44487	*lactamica*	OX40550	UK	2015	195	No
44488	*lactamica*	OX40595	UK	2015	707	No
44492	*lactamica*	OX40631	UK	2015	481	No
44494	*lactamica*	OX40633	UK	2015	472	No
44505	*lactamica*	OX40797	UK	2015	48	No
44506	*lactamica*	OX40810	UK	2015	480	No
44508	*lactamica*	OX40813	UK	2015	748	No
44511	*lactamica*	OX40841	UK	2015	94	No
44512	*lactamica*	OX40849	UK	2015	518	No
44515	*lactamica*	OX40886	UK	2015	94	No
44535	*lactamica*	OX41163	UK	2015	472	No
44546	*lactamica*	OX41248	UK	2015	749	No
44553	*lactamica*	OX41321	UK	2015	534	No
44555	*lactamica*	OX41331	UK	2015	750	No
44558	*lactamica*	OX41393	UK	2015	533	No
44560	*lactamica*	OX41455	UK	2015	751	No
44567	*lactamica*	OX41503	UK	2015	752	No
44569	*lactamica*	OX41505	UK	2015	753	No
44584	*lactamica*	OX41691	UK	2015	480	No
44590	*lactamica*	OX41708	UK	2015	480	No
44594	*lactamica*	OX41761	UK	2015	94	No
44605	*lactamica*	OX41871	UK	2015	532	No
44611	*lactamica*	OX41959	UK	2015	706	No
44622	*lactamica*	OX42059	UK	2015	736	No
44634	*lactamica*	OX42035	UK	2015	562	No
44922	*lactamica*	AR01252_T8	Ethiopia	2015	709	No
46053	*lactamica*	M31.5	Ireland	2013	250	No
46055	*lactamica*	M31.7	Ireland	2013	250	No
46056	*lactamica*	M31.8	Ireland	2013	250	No
46057	*lactamica*	M31.9	Ireland	2013	250	No
46062	*lactamica*	M35.6	Ireland	2013	250	No
46066	*lactamica*	M45.3	Ireland	2013	250	No
46070	*lactamica*	M45.7	Ireland	2013	250	No
46077	*lactamica*	M46.10	Ireland	2013	250	No
46267	*lactamica*	NS19	Unknown	Unknown	1896	No
46547	*lactamica*	PL41414	UK	2015	751	No
46551	*lactamica*	PL41609	UK	2015	517	No
46552	*lactamica*	GL40145	UK	2014	754	No
46557	*lactamica*	GL40222	UK	2014	476	No
46558	*lactamica*	GL40260	UK	2014	755	No

46563	*lactamica*	GL40437	UK	2014	706	No
46564	*lactamica*	GL40486	UK	2014	756	No
46580	*lactamica*	GL40670	UK	2014	561	No
46582	*lactamica*	GL40709	UK	2014	757	No
46601	*lactamica*	CA40805	UK	2015	562	No
46622	*lactamica*	GL41391	UK	2014	4	No
46629	*lactamica*	GL41566	UK	2014	564	No
46636	*lactamica*	GL41710	UK	2014	725	No
46640	*lactamica*	CA40176	UK	2015	567	No
46654	*lactamica*	GL41902	UK	2015	754	No
46655	*lactamica*	GL41923	UK	2015	472	No
46670	*lactamica*	GL42119	UK	2015	48	No
46671	*lactamica*	GL42134	UK	2015	758	No
46683	*lactamica*	GL42524	UK	2015	725	No
46685	*lactamica*	GL42545	UK	2015	480	No
46703	*lactamica*	GL42700	UK	2015	569	No
46712	*lactamica*	GL42800	UK	2015	4	No
46714	*lactamica*	GL42844	UK	2015	753	No
46715	*lactamica*	GL42847	UK	2015	48	No
46720	*lactamica*	GL42893	UK	2015	48	No
46731	*lactamica*	WG40094	UK	2015	744	No
49335	*lactamica*	030-24	UK	1999	710	No
49336	*lactamica*	014-24	UK	1998	94	No
49337	*lactamica*	09002S1	UK	1999	95	No
49338	*lactamica*	8206	UK	2000	96	No
49396	*lactamica*	CA40129	UK	2015	760	No
49402	*lactamica*	CA40181	UK	2015	472	No
49408	*lactamica*	CA40219	UK	2015	562	No
49410	*lactamica*	CA40250	UK	2015	562	No
49414	*lactamica*	CA40342	UK	2015	722	No
49418	*lactamica*	CA40375	UK	2015	562	No
49431	*lactamica*	CA40578	UK	2015	762	No
49434	*lactamica*	CA40591	UK	2015	480	No
49437	*lactamica*	CA40619	UK	2015	472	No
49454	*lactamica*	CA40839	UK	2015	562	No
49455	*lactamica*	CA40840	UK	2015	619	No
49458	*lactamica*	CA40852	UK	2015	48	No
49465	*lactamica*	CA40890	UK	2015	763	No
49475	*lactamica*	CA41014	UK	2015	710	No
49478	*lactamica*	CA41036	UK	2015	764	No
49522	*lactamica*	CA41703	UK	2015	754	No
49524	*lactamica*	CA41718	UK	2015	758	No
49532	*lactamica*	CA41793	UK	2015	706	No
49538	*lactamica*	CA41860	UK	2015	765	No
49541	*lactamica*	CA41874	UK	2015	766	No
49549	*lactamica*	CA41958	UK	2015	710	No
49566	*lactamica*	CA42332	UK	2015	767	No
49569	*lactamica*	CA42422	UK	2015	457	No
49598	*lactamica*	CA44022	UK	2015	480	No
49605	*lactamica*	CA44110	UK	2015	763	No
49608	*lactamica*	CA44136	UK	2015	624	No
49620	*lactamica*	CA44231	UK	2015	562	No

49626	*lactamica*	CA44279	UK	2015	625	No
49630	*lactamica*	CA44314	UK	2015	707	No
49633	*lactamica*	CA44339	UK	2015	NA	No
49642	*lactamica*	CA44417	UK	2015	768	No
49656	*lactamica*	CA44699	UK	2015	195	No
49667	*lactamica*	GL40581	UK	2014	4	No
49675	*lactamica*	GL41030	UK	2014	472	No
49682	*lactamica*	LO40016	UK	2014	626	No
49693	*lactamica*	LO40584	UK	2014	769	No
49694	*lactamica*	LO40696	UK	2015	770	No
49695	*lactamica*	LO40703	UK	2015	627	No
49698	*lactamica*	LO40800	UK	2015	706	No
49712	*lactamica*	LO41023	UK	2015	736	No
49715	*lactamica*	LO41456	UK	2015	771	No
49716	*lactamica*	LO41459	UK	2015	480	No
49718	*lactamica*	LO41523	UK	2015	758	No
49720	*lactamica*	CM40023	UK	Unknown	772	No
49731	*lactamica*	CM40386	UK	2015	706	No
49734	*lactamica*	CM40397	UK	2015	744	No
49743	*lactamica*	CM40479	UK	2015	628	No
49754	*lactamica*	CM40568	UK	Unknown	195	No
49769	*lactamica*	CM40742	UK	2015	561	No
49805	*lactamica*	CM41107	UK	2015	725	No
49821	*lactamica*	CM41804	UK	2015	725	No
49826	*lactamica*	CM41850	UK	2015	625	No
49840	*lactamica*	PL40033	UK	2015	709	No
49844	*lactamica*	PL40153	UK	2015	706	No
49845	*lactamica*	PL40181	UK	2015	4	No
49854	*lactamica*	PL40385	UK	2015	195	No
49864	*lactamica*	PL40553	UK	2015	631	No
49870	*lactamica*	PL40625	UK	2015	707	No
49873	*lactamica*	PL40640	UK	2015	773	No
49874	*lactamica*	PL40656	UK	2015	517	No
49881	*lactamica*	PL40811	UK	2015	472	No
49891	*lactamica*	PL40992	UK	2015	195	No
49900	*lactamica*	PL41038	UK	2015	48	No
49902	*lactamica*	PL41088	UK	2015	195	No
49903	*lactamica*	PL41098	UK	2015	706	No
49905	*lactamica*	PL41133	UK	2015	774	No
49908	*lactamica*	PL41154	UK	2015	725	No
49914	*lactamica*	PL41184	UK	2015	775	No
49916	*lactamica*	PL41185	UK	2015	776	No
49918	*lactamica*	PL41208	UK	2015	632	No
49919	*lactamica*	PL41248	UK	2015	633	No
49921	*lactamica*	PL41272	UK	2015	633	No
49923	*lactamica*	PL41312	UK	2015	633	No
49927	*lactamica*	PL41361	UK	2015	96	No
49933	*lactamica*	PL41413	UK	2015	96	No
49951	*lactamica*	PL41704	UK	2015	706	No
49962	*lactamica*	PL41845	UK	2015	736	No
49968	*lactamica*	PL42038	UK	2015	95	No
49972	*lactamica*	PL42083	UK	2015	744	No
50044	*lactamica*	PR40505	UK	2015	778	No
50106	*lactamica*	ST41803	UK	2015	48	No
50107	*lactamica*	ST42008	UK	2015	95	No
50116	*lactamica*	WG40033	UK	2015	195	No
50134	*lactamica*	WG40767	UK	2015	639	No
50151	*lactamica*	WG40922	UK	2015	640	No
50169	*lactamica*	WG41063	UK	2015	641	No
50178	*lactamica*	WG41095	UK	2015	642	No
50193	*lactamica*	WG41305	UK	2015	95	No

50194	*lactamica*	WG41313	UK	2015	779	No
50200	*lactamica*	WG41335	UK	2015	780	No
50207	*lactamica*	WG41366	UK	2015	725	No
50217	*lactamica*	WG41435	UK	2015	4	No
50232	*lactamica*	PL41597	UK	2015	517	No
50891	*lactamica*	MT40039	UK	2015	472	No
50899	*lactamica*	MT40094	UK	2015	4	No
50900	*lactamica*	MT40099	UK	2015	195	No
50922	*lactamica*	MT40444	UK	2015	654	No
50928	*lactamica*	MT40505	UK	2015	4	No
50929	*lactamica*	MT40522	UK	2015	707	No
50930	*lactamica*	MT40530	UK	2015	195	No
50933	*lactamica*	MT40550	UK	2015	195	No
50939	*lactamica*	PL41991	UK	2015	655	No
52580	*lactamica*	CA44016	UK	2015	781	No
52586	*lactamica*	CA44292	UK	2015	532	No
52723	*lactamica*	WG41050B	UK	2015	782	No
56428	*lactamica*	Y92-1009_Pacbio	UK	1992	48	No
58872	*lactamica*	LNP29217	France	2017	2002	No
59577	*lactamica*	TS1121	Australia	2017	2009	No
60878	*lactamica*	BRI012	UK	2017	997	No
60881	*lactamica*	BRI013	UK	2017	997	No
60886	*lactamica*	BRI001	UK	2017	754	No
60936	*lactamica*	BRI003	UK	2017	1582	No
60948	*lactamica*	BRI010	UK	2017	725	No
62970	*lactamica*	020-06	Unknown	Unknown	4	No
63702	*lactamica*	BT00461V1C	UK	2018	744	No
63704	*lactamica*	BT00531V1C	UK	2018	1517	No
63710	*lactamica*	BT03268V1C	UK	2018	709	No
63711	*lactamica*	BT03306V1C	UK	2018	95	No
63716	*lactamica*	BT03414V1C	UK	2018	754	No
63718	*lactamica*	BT03434V1C	UK	2018	724	No
63719	*lactamica*	BT03441V1C	UK	2018	1518	No
63730	*lactamica*	BT03704V1C	UK	2018	2021	No
63733	*lactamica*	BT03840V1C	UK	2018	997	No
63775	*lactamica*	BT15993V1C	UK	2018	2022	No
63793	*lactamica*	BT18109V1C	UK	2018	744	No
63801	*lactamica*	BT19039V1C	UK	2019	195	No
63805	*lactamica*	BT21081V1C	UK	2018	48	No
63809	*lactamica*	BT21312V1C	UK	2018	725	No
63811	*lactamica*	BT21394V1C	UK	2018	764	No
63812	*lactamica*	BT21394V1C2	UK	2018	764	No
63815	*lactamica*	BT21504V1C	UK	2018	1529	No
63817	*lactamica*	BT21514V1C	UK	2018	753	No
63834	*lactamica*	BT24326V1C	UK	2018	41	No
63835	*lactamica*	BT24377V1C	UK	2018	476	No
63838	*lactamica*	BT25131V1C	UK	2018	250	No
63848	*lactamica*	BT27247V1C	UK	2018	1530	No
63854	*lactamica*	BT27296V1C	UK	2018	2023	No
63860	*lactamica*	BT27361V1C	UK	2018	706	No
63865	*lactamica*	BT30159V1C	UK	2018	707	No
63866	*lactamica*	BT30398V1C	UK	2018	997	No
63871	*lactamica*	BT33019V1C	UK	2018	2024	No
63872	*lactamica*	BT33053V1C	UK	2018	731	No
63873	*lactamica*	BT33166V1C2	UK	2018	727	No
63879	*lactamica*	BT36010V1C	UK	2018	625	No
63882	*lactamica*	BT36097V3C	UK	2019	725	No
63913	*lactamica*	BT37078V1C	UK	2018	2025	No
63914	*lactamica*	BT37088V1C	UK	2018	2026	No
63915	*lactamica*	BT37109V1C	UK	2018	480	No
63919	*lactamica*	BT37209V1C	UK	2018	2027	No
63921	*lactamica*	BT37226V1C	UK	2018	1527	No
63924	*lactamica*	BT38139V1C	UK	2018	1526	No
63934	*lactamica*	BT38320V1C	UK	2018	4	No
63946	*lactamica*	BT39540V1C	UK	2018	2028	No
63950	*lactamica*	BT39581V1C	UK	2018	1524	No
63957	*lactamica*	BT39730V1C	UK	2019	754	No
63965	*lactamica*	BT39827V1C	UK	2019	780	No

63967	*lactamica*	BT51011V1C	UK	2018	562	No
71179	*lactamica*	BT00010V1C	UK	2018	195	No
71180	*lactamica*	BT00405V1C	UK	2018	2036	No
71181	*lactamica*	BT00500V1C	UK	2018	2037	No
71186	*lactamica*	BT03638V1C	UK	2018	754	No
71187	*lactamica*	BT03760V1C	UK	2018	1992	No
71198	*lactamica*	BT16201V1C	UK	2018	1531	No
71200	*lactamica*	BT18874V1C	UK	2019	2039	No
71204	*lactamica*	BT19030V1C	UK	2019	2040	No
71206	*lactamica*	BT19060V1C	UK	2019	744	No
71209	*lactamica*	BT21037V1C	UK	2018	480	No
71215	*lactamica*	BT21839V1C	UK	2018	1532	No
71216	*lactamica*	BT25203V1C	UK	2018	4	No
71218	*lactamica*	BT27016V1C	UK	2018	1533	No
71223	*lactamica*	BT30039V1C	UK	2018	725	No
71228	*lactamica*	BT30666V1C	UK	2018	1891	No
71229	*lactamica*	BT30775V1C	UK	2018	1582	No
71230	*lactamica*	BT30831V1C	UK	2018	517	No
71233	*lactamica*	BT36010V3C	UK	2019	625	No
71234	*lactamica*	BT36089V1C	UK	2018	754	No
71235	*lactamica*	BT36094V1C	UK	2018	997	No
71236	*lactamica*	BT36094V3C	UK	2019	997	No
71261	*lactamica*	BT38172V1C	UK	2018	195	No
71271	*lactamica*	BT6333V1C	UK	2018	710	No
71276	*lactamica*	BT00050V1C	UK	2018	480	No
71277	*lactamica*	BT00052V1C	UK	2018	1506	No
71282	*lactamica*	BT03358V1C	UK	2018	2041	No
71300	*lactamica*	BT09034V1C	UK	2019	562	No
71302	*lactamica*	BT09131V1C	UK	2019	709	No
71317	*lactamica*	BT16510V1C	UK	2018	94	No
71318	*lactamica*	BT16646V1C	UK	2018	195	No
71320	*lactamica*	BT19013V1C	UK	2019	4	No
71323	*lactamica*	BT21389V1C	UK	2018	764	No
71331	*lactamica*	BT27243V1C	UK	2018	2043	No
71332	*lactamica*	BT27321V1C	UK	2018	94	No
71340	*lactamica*	BT30572V1C	UK	2018	706	No
71342	*lactamica*	BT30672V1C	UK	2018	1582	No
71344	*lactamica*	BT30761V1C	UK	2018	4	No
71345	*lactamica*	BT30850V1C	UK	2018	NA	No
71347	*lactamica*	BT33381V1C	UK	2018	736	No
71357	*lactamica*	BT36732V1C	UK	2018	195	No
71362	*lactamica*	BT38516V1C	UK	2019	725	No
82614	*lactamica*	ExNm704	Australia	Unknown	195	No
82615	*lactamica*	ExNm705	Australia	Unknown	2081	No
82616	*lactamica*	ExNm706	Australia	Unknown	2081	No
82620	*lactamica*	ExNm712	Australia	Unknown	456	No
82622	*lactamica*	ExNm714	Australia	Unknown	1874	No
82627	*lactamica*	ExNm720	Australia	Unknown	706	No
83066	*lactamica*	Nei0341	China	2016	990	No
83682	*lactamica*	KL1-161	South Africa	2016	2082	No
83684	*lactamica*	KL1-118	South Africa	2016	2083	No
84195	*lactamica*	Nei0012	China	2013	2085	No
84198	*lactamica*	Nei0018	China	2013	2086	No
84202	*lactamica*	Nei0033	China	2014	990	No
84206	*lactamica*	Nei0042	China	2014	2087	No
84261	*lactamica*	Nei0422	China	2018	510	No
84265	*lactamica*	Nei0443	China	2014	2096	No
84474	*lactamica*	Nei0110	China	2014	NA	No
84477	*lactamica*	Nei0139	China	2015	2104	No
84478	*lactamica*	Nei0145	China	2015	95	No
85858	*lactamica*	EF-285	Greece	2017	195	No
88405	*lactamica*	BT00168V1C	UK	2018	4	No
88406	*lactamica*	BT00207V1C	UK	2018	758	No
88411	*lactamica*	BT00257V1C	UK	2018	987	No
88420	*lactamica*	BT00414V1C	UK	2018	2118	No
88426	*lactamica*	BT03055V1C	UK	2018	989	No
88431	*lactamica*	BT03335V1C	UK	2018	706	No
88446	*lactamica*	BT18057V1C	UK	2018	561	No

88453	*lactamica*	BT18163V1C	UK	2018	625	No
88455	*lactamica*	BT18185V1C	UK	2018	624	No
88457	*lactamica*	BT18228V1C	UK	2018	988	No
88458	*lactamica*	BT18245V1C	UK	2018	754	No
88461	*lactamica*	BT18442V1C	UK	2018	733	No
88465	*lactamica*	BT18516V1C	UK	2018	625	No
88472	*lactamica*	BT18633V1C	UK	2018	195	No
88473	*lactamica*	BT18638V1C	UK	2018	709	No
88475	*lactamica*	BT18649V1C	UK	2018	2119	No
88482	*lactamica*	BT18692V1C	UK	2018	990	No
88483	*lactamica*	BT18716V1C	UK	2018	725	No
88491	*lactamica*	BT21019V1C	UK	2018	781	No
88493	*lactamica*	BT21047V1C	UK	2018	992	No
88504	*lactamica*	BT21252V1C	UK	2018	1874	No
88517	*lactamica*	BT21831V1C	UK	2018	725	No
88524	*lactamica*	BT21968V1C	UK	2018	2120	No
88526	*lactamica*	BT21997V1C	UK	2018	364	No
88527	*lactamica*	BT22034V1C	UK	2018	753	No
88530	*lactamica*	BT22114V1C	UK	2018	994	No
88534	*lactamica*	BT22244V1C	UK	2018	NA	No
88547	*lactamica*	BT24241V1C	UK	2018	94	No
88557	*lactamica*	BT24555V1C	UK	2018	995	No
88559	*lactamica*	BT24644V1C2	UK	2018	517	No
88562	*lactamica*	BT24726V1C	UK	2018	725	No
88569	*lactamica*	BT24798V1C	UK	2018	2039	No
88570	*lactamica*	BT24860V1C	UK	2018	725	No
88580	*lactamica*	BT25002V1C	UK	2018	48	No
88587	*lactamica*	BT25065V1C	UK	2018	250	No
88591	*lactamica*	BT25137V1C	UK	2018	250	No
88594	*lactamica*	BT25223V1C	UK	2018	472	No
88596	*lactamica*	BT25268V1C	UK	2018	709	No
88598	*lactamica*	BT30034V1C	UK	2018	2121	No
88599	*lactamica*	BT30105V1C	UK	2018	2122	No
88606	*lactamica*	BT30272V1C	UK	2018	997	No
88609	*lactamica*	BT33032V1C	UK	2018	709	No
88612	*lactamica*	BT33184V1C	UK	2018	2123	No
88613	*lactamica*	BT33243V1C	UK	2018	94	No
88616	*lactamica*	BT36018V1C	UK	2018	754	No
88619	*lactamica*	BT36099V1C	UK	2018	2027	No
88634	*lactamica*	BT36341V1C	UK	2018	725	No
88639	*lactamica*	BT36377V1C	UK	2018	2118	No
88642	*lactamica*	BT36387V1C	UK	2018	2124	No
88651	*lactamica*	BT36807V1C2	UK	2018	94	No
88659	*lactamica*	BT37076V1C	UK	2018	2125	No
88660	*lactamica*	BT37156V1C	UK	2018	2025	No
88663	*lactamica*	BT37266V1C	UK	2018	733	No
88666	*lactamica*	BT37413V1C	UK	2018	195	No
88670	*lactamica*	BT37513V1C	UK	2018	4	No
88682	*lactamica*	BT37703V1C	UK	2018	364	No
88685	*lactamica*	BT37756V1C	UK	2018	997	No
88686	*lactamica*	BT37760V1C	UK	2018	998	No
88691	*lactamica*	BT37849V1C	UK	2018	725	No
88695	*lactamica*	BT37921V1C	UK	2018	480	No
88698	*lactamica*	BT37982V1C	UK	2018	725	No
88704	*lactamica*	BT38065V1C	UK	2018	2126	No
88706	*lactamica*	BT38074V1C	UK	2018	999	No
88716	*lactamica*	BT39220V1C	UK	2018	2039	No
88722	*lactamica*	BT39320V1C	UK	2018	753	No
88731	*lactamica*	BT39463V1C	UK	2018	364	No
88738	*lactamica*	BT51142V1C	UK	2018	2127	No
88739	*lactamica*	BT51148V1C	UK	2018	2127	No
88744	*lactamica*	BT6115V1C	UK	2018	754	No
88764	*lactamica*	BT6710V1C	UK	2018	763	No
88770	*lactamica*	BT6785V1C	UK	2018	754	No
88780	*lactamica*	BT6997V1C	UK	2018	4	No
88789	*lactamica*	M37058	Brazil	2014	95	No
88790	*lactamica*	M37063	Brazil	2014	2128	No
88791	*lactamica*	M37071	Brazil	2014	2128	No

88792	*lactamica*	M37073	Brazil	2014	725	No
88793	*lactamica*	M37078	Brazil	2014	707	No
88794	*lactamica*	M37079	Brazil	2014	480	No
88795	*lactamica*	M37083	Brazil	2014	2128	No
88796	*lactamica*	M37084	Brazil	2014	1895	No
88797	*lactamica*	M37091	Brazil	2014	2129	No
88798	*lactamica*	M37094	Brazil	2014	733	No
88799	*lactamica*	M37101	Brazil	2014	709	No
88800	*lactamica*	M37105	Brazil	2014	733	No
88801	*lactamica*	M37107	Brazil	2014	2130	No
88802	*lactamica*	M37110	Brazil	2014	2129	No
88803	*lactamica*	M37119	Brazil	2014	2131	No
88804	*lactamica*	M37123	Brazil	2014	1992	No
88805	*lactamica*	M37130	Brazil	2014	2128	No
88806	*lactamica*	M37131	Brazil	2014	95	No
88807	*lactamica*	M37132	Brazil	2014	725	No
88808	*lactamica*	M37136	Brazil	2014	2131	No
88809	*lactamica*	M37137	Brazil	2014	709	No
88810	*lactamica*	M37140	Brazil	2014	725	No
88811	*lactamica*	M37142	Brazil	2014	2128	No
88812	*lactamica*	M37143	Brazil	2014	2131	No
88813	*lactamica*	M37146	Brazil	2014	2128	No
88814	*lactamica*	M37147	Brazil	2014	2131	No
88815	*lactamica*	M37155	Brazil	2014	2132	No
88816	*lactamica*	M37157	Brazil	2014	725	No
88817	*lactamica*	M37158	Brazil	2014	2133	No
88818	*lactamica*	M37159	Brazil	2014	1896	No
88819	*lactamica*	M37160	Brazil	2014	2134	No
88820	*lactamica*	M37162	Brazil	2014	767	No
88821	*lactamica*	M37163	Brazil	2014	725	No
88822	*lactamica*	M37164	Brazil	2014	2135	No
88823	*lactamica*	M37166	Brazil	2014	725	No
88824	*lactamica*	M37167	Brazil	2014	1942	No
88825	*lactamica*	M37168	Brazil	2014	2136	No
88826	*lactamica*	M37169	Brazil	2014	95	No
88827	*lactamica*	M37170	Brazil	2014	725	No
88828	*lactamica*	M37171	Brazil	2014	725	No
88829	*lactamica*	M37172	Brazil	2014	2137	No
88830	*lactamica*	M37173	Brazil	2014	725	No
88831	*lactamica*	M37174	Brazil	2014	532	No
88832	*lactamica*	M37175	Brazil	2014	1896	No
88833	*lactamica*	M37176	Brazil	2014	95	No
88834	*lactamica*	M37177	Brazil	2014	2138	No
88835	*lactamica*	M37178	Brazil	2014	725	No
88836	*lactamica*	M37179	Brazil	2014	725	No
88837	*lactamica*	M37957	Brazil	2014	2131	No
88838	*lactamica*	M37161	Brazil	2014	725	No
89163	*lactamica*	Nei0082	China	2014	990	No
89164	*lactamica*	Nei0153	China	2015	990	No
89165	*lactamica*	Nei0343	China	2016	708	No
89760	*lactamica*	MB15646	Australia	2017	195	No
90078	*lactamica*	MB27896	Australia	2017	48	No
90341	*lactamica*	MB37068	Australia	2017	2145	No
90480	*lactamica*	MB41129A	Australia	2017	48	No
90541	*lactamica*	MB44238	Australia	2017	195	No
92872	*lactamica*	Nei0068	China	2014	2158	No
92873	*lactamica*	Nei0090	China	2014	990	No
92874	*lactamica*	Nei0198	China	2015	2159	No
92875	*lactamica*	Nei0409	China	2016	NA	No
105709	*lactamica*	Nei0171	China	2015	NA	No
105710	*lactamica*	Nei0264	China	2016	725	No
108165	*lactamica*	Nei0157	China	2015	990	No
108166	*lactamica*	Nei0386	China	2016	510	No
111269	*lactamica*	Nei0152	China	2015	95	No
111270	*lactamica*	Nei0282	China	2016	1680	No
111271	*lactamica*	Nei0432	China	2016	2228	No
111272	*lactamica*	Nei0439	China	2014	990	No
115357	*lactamica*	BT03414V3C	UK	2019	754	No

115368	*lactamica*	BT04272V3C	UK	2020	2118	No
115369	*lactamica*	BT04288V1C	UK	2019	2118	No
115394	*lactamica*	BT18228V3C	UK	2019	733	No
115396	*lactamica*	BT18245V3C	UK	2020	754	No
115398	*lactamica*	BT18375V3C	UK	2019	725	No
115399	*lactamica*	BT18381V3C	UK	2019	725	No
115410	*lactamica*	BT18521V3C	UK	2019	2235	No
115420	*lactamica*	BT18768V3C	UK	2020	NA	No
115425	*lactamica*	BT21001V3C	UK	2019	706	No
115432	*lactamica*	BT21088V3C	UK	2019	625	No
115450	*lactamica*	BT21252V3C	UK	2019	1874	No
115460	*lactamica*	BT21389V3C	UK	2019	764	No
115480	*lactamica*	BT21521V3C	UK	2019	1874	No
115484	*lactamica*	BT21557V3C	UK	2019	997	No
115502	*lactamica*	BT21747V3C	UK	2019	1504	No
115505	*lactamica*	BT21831V3C	UK	2019	725	No
115511	*lactamica*	BT27691V1C	UK	2020	1496	No
115517	*lactamica*	BT27825V1C	UK	2020	727	No
115524	*lactamica*	BT36432V3C	UK	2019	48	No
115536	*lactamica*	BT36660V3C	UK	2019	709	No
115537	*lactamica*	BT36675V3C	UK	2019	663	No
115541	*lactamica*	BT36706V3C	UK	2019	725	No
115552	*lactamica*	BT36807V3C	UK	2019	94	No
115567	*lactamica*	BT37017V3C	UK	2019	734	No
115569	*lactamica*	BT37076V3C	UK	2019	725	No
115575	*lactamica*	BT37145V3C	UK	2019	94	No
115584	*lactamica*	BT37209V3C	UK	2019	2027	No
115641	*lactamica*	BT38320V3C	UK	2019	4	No
115643	*lactamica*	BT39009V3C	UK	2019	706	No
115665	*lactamica*	BT39277V3C	UK	2019	706	No
115674	*lactamica*	BT39462V3C	UK	2019	725	No
115679	*lactamica*	BT39487V3C	UK	2019	476	No
115698	*lactamica*	BT39711V3C	UK	2020	2134	No
115715	*lactamica*	BT39985V1C	UK	2020	1498	No
115731	*lactamica*	BT51572V1C	UK	2019	706	No
120674	*lactamica*	BT00050V3C	UK	2019	480	No
120688	*lactamica*	BT00192V3C	UK	2019	734	No
120695	*lactamica*	BT00257V3C	UK	2019	987	No
120707	*lactamica*	BT00531V3C	UK	2019	1517	No
120724	*lactamica*	BT00828V3C	UK	2020	997	No
120727	*lactamica*	BT03306V3C	UK	2019	95	No
120746	*lactamica*	BT09274V3C	UK	2020	1486	No
120752	*lactamica*	BT09312V3C	UK	2020	736	No
120790	*lactamica*	BT16661V3C	UK	2019	41	No
120841	*lactamica*	BT30031V3C	UK	2019	2241	No
120843	*lactamica*	BT30039V3C	UK	2019	725	No
120844	*lactamica*	BT30061V3C	UK	2019	706	No
120845	*lactamica*	BT30105V3C	UK	2019	2122	No
120859	*lactamica*	BT33076V3C	UK	2019	740	No
122310	*lactamica*	BT00010V3C	UK	2019	195	No
122314	*lactamica*	BT00052V3C	UK	2019	1506	No
122318	*lactamica*	BT00273V3C	UK	2019	997	No
122333	*lactamica*	BT00451V3C	UK	2019	NA	No
122335	*lactamica*	BT00502V3C	UK	2019	734	No
122343	*lactamica*	BT00730V1C	UK	2019	997	No
122352	*lactamica*	BT00768V1C	UK	2019	736	No
122361	*lactamica*	BT00828V1C	UK	2019	997	No
122379	*lactamica*	BT15758V3C	UK	2019	94	No
122382	*lactamica*	BT15946V3C	UK	2019	195	No
122384	*lactamica*	BT16021V3C	UK	2020	2118	No
122385	*lactamica*	BT16045V3C	UK	2019	706	No
122387	*lactamica*	BT16353V3C	UK	2019	2242	No
122395	*lactamica*	BT18351V3C	UK	2019	1498	No
122403	*lactamica*	BT22117V3C	UK	2019	758	No
122404	*lactamica*	BT22128V3C	UK	2019	364	No
122405	*lactamica*	BT24031V3C	UK	2019	4	No
122424	*lactamica*	BT24347V3C	UK	2019	1508	No
122426	*lactamica*	BT24373V3C	UK	2019	736	No

122437	*lactamica*	BT24644V3C	UK	2020	480	No
122441	*lactamica*	BT24685V3C	UK	2019	736	No
122445	*lactamica*	BT24707V3C	UK	2019	2241	No
122448	*lactamica*	BT24743V3C	UK	2019	1560	No
122451	*lactamica*	BT24914V3C	UK	2019	48	No
122452	*lactamica*	BT24916V3C	UK	2019	997	No
122456	*lactamica*	BT24959V3C	UK	2019	997	No
122462	*lactamica*	BT25065V3C	UK	2019	250	No
122468	*lactamica*	BT25154V3C	UK	2019	195	No
122472	*lactamica*	BT25194V3C	UK	2019	1511	No
122475	*lactamica*	BT25268V3C	UK	2019	709	No
122480	*lactamica*	BT25475V3C	UK	2020	48	No
122483	*lactamica*	BT25500V3C	UK	2020	2243	No
122486	*lactamica*	BT27123V3C	UK	2019	2244	No
122487	*lactamica*	BT27199V3C	UK	2019	562	No
122497	*lactamica*	BT30137V3C	UK	2019	997	No
122498	*lactamica*	BT30272V3C	UK	2019	997	No
122503	*lactamica*	BT30572V3C	UK	2020	706	No
122504	*lactamica*	BT30579V3C	UK	2020	663	No
122505	*lactamica*	BT30656V3C	UK	2020	706	No
122529	*lactamica*	BT33456V3C	UK	2019	706	No
122532	*lactamica*	BT33530V1C	UK	2019	4	No
122534	*lactamica*	BT33564V1C	UK	2019	1891	No
122568	*lactamica*	BT38121V3C	UK	2019	96	No
122577	*lactamica*	BT38247V3C	UK	2019	626	No
122584	*lactamica*	BT40026V1C	UK	2019	2245	No
122591	*lactamica*	BT50602V1C	UK	2019	706	No
122594	*lactamica*	BT50665V1C	UK	2019	94	No
122595	*lactamica*	BT50760V1C	UK	2019	2246	No
122612	*lactamica*	M17105	Unknown	Unknown	1514	No
122613	*lactamica*	M17106	Unknown	Unknown	710	No
122614	*lactamica*	NCTC10616	USA	1965	1515	No
122615	*lactamica*	NCTC10617	USA	1967	1874	No
122616	*lactamica*	NCTC10618	Unknown	2014	2247	No
122617	*lactamica*	NIUS07x1	USA	2007	1894	No
123025	*lactamica*	Co000761/1	Belgium	2020	2041	No
123044	*lactamica*	Co000771/1	Belgium	2020	1548	No
131428	*lactamica*	BT6517V3C	UK	2019	709	No
131446	*lactamica*	BT07245V3C	UK	2020	736	No
131469	*lactamica*	BT15597V3C	UK	2019	754	No
131487	*lactamica*	BT24007V3C	UK	2019	472	No
131495	*lactamica*	BT24198V3C	UK	2019	2041	No
131515	*lactamica*	BT25223V3C	UK	2019	472	No
131517	*lactamica*	BT30165V3C	UK	2019	736	No
131520	*lactamica*	BT33166V3C	UK	2019	727	No
131524	*lactamica*	BT07193V1C	UK	2019	706	No
131525	*lactamica*	BT07245V1C	UK	2019	736	No
131526	*lactamica*	BT07257V1C	UK	2019	710	No
131527	*lactamica*	BT16920V1C	UK	2019	1627	No
131535	*lactamica*	BT25459V1C	UK	2019	725	No
131610	*lactamica*	01MO1	UK	2023	997	No
131611	*lactamica*	01MO1	UK	2023	997	No
131612	*lactamica*	06MO1	UK	2023	2262	No
131613	*lactamica*	06MO1	UK	2023	2262	No
131614	*lactamica*	11MO1	UK	2023	195	No
131615	*lactamica*	11MO1	UK	2023	195	No
131616	*lactamica*	02MO3	UK	2023	48	No
131617	*lactamica*	04MO3	UK	2023	48	No
131618	*lactamica*	04MO3	UK	2023	48	No
131619	*lactamica*	04MO3	UK	2023	48	No
131620	*lactamica*	17MO1	UK	2023	997	No
131621	*lactamica*	17MO1	UK	2023	997	No
131622	*lactamica*	07MO3	UK	2023	48	No
131623	*lactamica*	07MO3	UK	2023	48	No
131624	*lactamica*	07MO3	UK	2023	48	No
131625	*lactamica*	04MO4	UK	2023	48	No
131626	*lactamica*	04MO4	UK	2023	48	No
131627	*lactamica*	04MO4	UK	2023	48	No

131628	*lactamica*	13MO3	UK	2023	48	No
131629	*lactamica*	13MO3	UK	2023	48	No
131630	*lactamica*	13MO3	UK	2023	48	No
131631	*lactamica*	03MO3	UK	2023	48	No
131632	*lactamica*	03MO3	UK	2023	48	No
131633	*lactamica*	06MO5	UK	2023	2262	No
131634	*lactamica*	06MO5	UK	2023	2262	No
131635	*lactamica*	06MO5	UK	2023	2262	No
131636	*lactamica*	10MO3	UK	2023	48	No
131637	*lactamica*	10MO3	UK	2023	48	No
131638	*lactamica*	10MO3	UK	2023	48	No
131639	*lactamica*	12MO3	UK	2023	48	No
131640	*lactamica*	12MO3	UK	2023	48	No
131641	*lactamica*	12MO3	UK	2023	48	No
131642	*lactamica*	10IS3	UK	2023	48	No
131643	*lactamica*	10IS3	UK	2023	48	No
131644	*lactamica*	10IS3	UK	2023	48	No
131645	*lactamica*	04MO5	UK	2023	48	No
131646	*lactamica*	04MO5	UK	2023	48	No
131647	*lactamica*	04MO5	UK	2023	48	No
131648	*lactamica*	11IS5	UK	2023	195	No
131649	*lactamica*	11IS5	UK	2023	195	No
131650	*lactamica*	11IS5	UK	2023	195	No
131651	*lactamica*	07MO5	UK	2023	48	No
131652	*lactamica*	07MO5	UK	2023	48	No
131653	*lactamica*	07MO5	UK	2023	48	No
131654	*lactamica*	14MO3	UK	2023	48	No
131655	*lactamica*	14MO3	UK	2023	48	No
131656	*lactamica*	14MO3	UK	2023	48	No
131657	*lactamica*	01MO5	UK	2023	997	No
131658	*lactamica*	01MO5	UK	2023	997	No
131659	*lactamica*	01MO5	UK	2023	997	No
131660	*lactamica*	09MO5	UK	2023	48	No
131661	*lactamica*	09MO5	UK	2023	48	No
131662	*lactamica*	09MO5	UK	2023	48	No
131663	*lactamica*	13MO5	UK	2023	48	No
131664	*lactamica*	13MO5	UK	2023	48	No
131665	*lactamica*	13MO5	UK	2023	48	No
131666	*lactamica*	10MO5	UK	2023	48	No
131667	*lactamica*	10MO5	UK	2023	48	No
131668	*lactamica*	10MO5	UK	2023	48	No
131669	*lactamica*	12MO5	UK	2023	48	No
131670	*lactamica*	12MO5	UK	2023	48	No
131671	*lactamica*	12MO5	UK	2023	48	No
131672	*lactamica*	17MO5	UK	2023	997	No
131673	*lactamica*	17MO5	UK	2023	997	No
131674	*lactamica*	17MO5	UK	2023	997	No
131675	*lactamica*	18MO3	UK	2023	48	No
131676	*lactamica*	18MO3	UK	2023	48	No
131677	*lactamica*	18MO3	UK	2023	48	No
131678	*lactamica*	14MO5	UK	2023	48	No
131679	*lactamica*	14MO5	UK	2023	48	No
131680	*lactamica*	14MO5	UK	2023	48	No
131681	*lactamica*	08MO3	UK	2023	48	No
131682	*lactamica*	21MO3	UK	2023	48	No
131683	*lactamica*	18MO4	UK	2023	48	No
131684	*lactamica*	18MO4	UK	2023	48	No
131685	*lactamica*	18MO4	UK	2023	48	No
131686	*lactamica*	24MO1	UK	2023	997	No
131687	*lactamica*	24MO1	UK	2023	997	No
131688	*lactamica*	28MO1	UK	2023	2121	No
131689	*lactamica*	28MO1	UK	2023	2121	No
131690	*lactamica*	27MO1	UK	2023	997	No
131691	*lactamica*	27MO1	UK	2023	997	No
131692	*lactamica*	20MO3	UK	2023	48	No
131693	*lactamica*	20MO3	UK	2023	48	No
131694	*lactamica*	20MO3	UK	2023	48	No
131695	*lactamica*	18MO5	UK	2023	48	No

131696	*lactamica*	18MO5	UK	2023	48	No
131697	*lactamica*	18MO5	UK	2023	48	No
131698	*lactamica*	11IS6	UK	2023	195	No
131699	*lactamica*	11IS6	UK	2023	195	No
131700	*lactamica*	11IS6	UK	2023	195	No
131701	*lactamica*	20MO5	UK	2023	48	No
131702	*lactamica*	20MO5	UK	2023	48	No
131703	*lactamica*	20MO5	UK	2023	48	No
131704	*lactamica*	07MO6	UK	2023	48	No
131705	*lactamica*	07MO6	UK	2023	48	No
131706	*lactamica*	07MO6	UK	2023	48	No
131707	*lactamica*	28MO4	UK	2023	2121	No
131708	*lactamica*	28MO4	UK	2023	2121	No
131709	*lactamica*	28MO4	UK	2023	2121	No
131710	*lactamica*	26MO3	UK	2023	48	No
131711	*lactamica*	26MO3	UK	2023	48	No
131712	*lactamica*	26MO3	UK	2023	48	No
131713	*lactamica*	13MO6	UK	2023	48	No
131714	*lactamica*	13MO6	UK	2023	48	No
131715	*lactamica*	13MO6	UK	2023	48	No
131716	*lactamica*	01MO6	UK	2023	997	No
131717	*lactamica*	01MO6	UK	2023	997	No
131718	*lactamica*	01MO6	UK	2023	997	No
131719	*lactamica*	01CS6	UK	2023	706	No
131720	*lactamica*	01CS6	UK	2023	706	No
131721	*lactamica*	01CS6	UK	2023	706	No
131722	*lactamica*	27MO3	UK	2023	997	No
131723	*lactamica*	27MO3	UK	2023	997	No
131724	*lactamica*	27MO3	UK	2023	997	No
131725	*lactamica*	09MO6	UK	2023	48	No
131726	*lactamica*	09MO6	UK	2023	48	No
131727	*lactamica*	09MO6	UK	2023	48	No
131728	*lactamica*	03MO6	UK	2023	48	No
131729	*lactamica*	03MO6	UK	2023	48	No
131730	*lactamica*	03MO6	UK	2023	48	No
131731	*lactamica*	24MO4	UK	2023	997	No
131732	*lactamica*	24MO4	UK	2023	997	No
131733	*lactamica*	24MO4	UK	2023	997	No
131734	*lactamica*	06MO6	UK	2023	2262	No
131735	*lactamica*	06MO6	UK	2023	2262	No
131736	*lactamica*	06MO6	UK	2023	2262	No
131737	*lactamica*	06CS6	UK	2023	2039	No
131738	*lactamica*	06CS6	UK	2023	2039	No
131739	*lactamica*	06CS6	UK	2023	2039	No
131740	*lactamica*	10CS6	UK	2023	1625	No
131741	*lactamica*	10CS6	UK	2023	1625	No
131742	*lactamica*	10CS6	UK	2023	1625	No
131743	*lactamica*	27MO4	UK	2023	997	No
131744	*lactamica*	27MO4	UK	2023	997	No
131745	*lactamica*	27MO4	UK	2023	997	No
131746	*lactamica*	10MO6	UK	2023	48	No
131747	*lactamica*	10MO6	UK	2023	48	No
131748	*lactamica*	10MO6	UK	2023	48	No
131749	*lactamica*	29MO3	UK	2023	48	No
131750	*lactamica*	29MO3	UK	2023	48	No
131751	*lactamica*	29MO3	UK	2023	48	No
131752	*lactamica*	12MO6	UK	2023	48	No
131753	*lactamica*	12MO6	UK	2023	48	No
131754	*lactamica*	12MO6	UK	2023	48	No
131755	*lactamica*	17MO6	UK	2023	997	No
131756	*lactamica*	17MO6	UK	2023	997	No
131757	*lactamica*	17MO6	UK	2023	997	No
131758	*lactamica*	26MO5	UK	2023	48	No
131759	*lactamica*	26MO5	UK	2023	48	No
131760	*lactamica*	26MO5	UK	2023	48	No
131761	*lactamica*	14MO6	UK	2023	48	No
131762	*lactamica*	14MO6	UK	2023	48	No
131763	*lactamica*	14MO6	UK	2023	48	No

131764	*lactamica*	29MO5	UK	2023	48	No
131765	*lactamica*	29MO5	UK	2023	48	No
131766	*lactamica*	29MO5	UK	2023	48	No
131767	*lactamica*	20MO6	UK	2023	48	No
131768	*lactamica*	20MO6	UK	2023	48	No
131769	*lactamica*	20MO6	UK	2023	48	No
131770	*lactamica*	28CS6	UK	2023	2121	No
131771	*lactamica*	28CS6	UK	2023	2121	No
131772	*lactamica*	28CS6	UK	2023	2121	No
131773	*lactamica*	26MO6	UK	2023	48	No
131774	*lactamica*	26MO6	UK	2023	48	No
131775	*lactamica*	26MO6	UK	2023	48	No
131776	*lactamica*	29MO6	UK	2023	48	No
131777	*lactamica*	29MO6	UK	2023	48	No
131778	*lactamica*	29MO6	UK	2023	48	No
131779	*lactamica*	24MO6	UK	2023	997	No
131780	*lactamica*	24MO6	UK	2023	997	No
131781	*lactamica*	24MO6	UK	2023	997	No
131782	*lactamica*	24CS6	UK	2023	997	No
131783	*lactamica*	24CS6	UK	2023	997	No
131784	*lactamica*	24CS6	UK	2023	997	No
131785	*lactamica*	901MO	UK	2023	997	No
131786	*lactamica*	906MO	UK	2023	2262	No
131787	*lactamica*	911MO	UK	2023	195	No
131788	*lactamica*	917MO	UK	2023	997	No
131789	*lactamica*	924MO	UK	2023	997	No
131790	*lactamica*	928MO	UK	2023	2121	No
131791	*lactamica*	927MO	UK	2023	997	No
131792	*lactamica*	LyoN127	UK	2023	48	No
144072	*lactamica*	BT00965V1C	UK	2019	754	No
144073	*lactamica*	BT00983V1C	UK	2019	736	No
144084	*lactamica*	BT01137V1C	UK	2019	2121	No
144085	*lactamica*	BT01138V1C	UK	2019	706	No
144086	*lactamica*	BT01175V1C	UK	2019	725	No
144087	*lactamica*	BT01216V1C	UK	2019	1678	No
144096	*lactamica*	BT04749V1C	UK	2019	625	No
144104	*lactamica*	BT07097V1C	UK	2019	725	No
144107	*lactamica*	BT07201V1C	UK	2019	4	No
144137	*lactamica*	BT07559V1C	UK	2019	725	No
144139	*lactamica*	BT07604V1C	UK	2019	725	No
144147	*lactamica*	BT07701V1C	UK	2019	1860	No
144148	*lactamica*	BT07706V1C	UK	2019	1861	No
144168	*lactamica*	BT17411V1C	UK	2019	2242	No
144194	*lactamica*	BT27482V1C	UK	2019	736	No
144198	*lactamica*	BT27576V1C	UK	2019	758	No
144208	*lactamica*	BT38559V1C	UK	2019	2126	No
144211	*lactamica*	BT50570V1C	UK	2019	1864	No
144219	*lactamica*	BT50812V1C	UK	2019	2309	No
144222	*lactamica*	BT50878V1C	UK	2019	758	No
144223	*lactamica*	BT50886V1C	UK	2019	1865	No
144228	*lactamica*	BT50922V1C	UK	2019	48	No
147836	*lactamica*	BT00863V1C	UK	2019	195	No
147838	*lactamica*	BT01065V1C	UK	2019	754	No
147847	*lactamica*	BT04160V1C	UK	2019	754	No
147849	*lactamica*	BT04228V1C	UK	2019	2337	No
147852	*lactamica*	BT04236V1C	UK	2019	2337	No
147854	*lactamica*	BT04244V1C	UK	2019	2337	No
147859	*lactamica*	BT04655V1C	UK	2019	1891	No
147866	*lactamica*	BT07211V1C	UK	2019	997	No
147867	*lactamica*	BT07219V1C	UK	2019	95	No
147868	*lactamica*	BT07227V1C	UK	2019	733	No
147876	*lactamica*	BT07570V1C	UK	2019	725	No
147877	*lactamica*	BT07687V1C	UK	2019	725	No
147879	*lactamica*	BT07859V1C	UK	2019	725	No
147883	*lactamica*	BT07935V1C	UK	2019	96	No
147884	*lactamica*	BT07942V1C	UK	2019	2338	No
147887	*lactamica*	BT09451V1C	UK	2019	2339	No
147888	*lactamica*	BT13017V1C	UK	2019	2241	No

147889	*lactamica*	BT13054V1C	UK	2019	736	No
147890	*lactamica*	BT13092V1C	UK	2019	736	No
147893	*lactamica*	BT16882V1C	UK	2018	2121	No
147897	*lactamica*	BT17440V1C	UK	2019	2340	No
147900	*lactamica*	BT17472V1C	UK	2019	94	No
147906	*lactamica*	BT17758V1C	UK	2019	997	No
147907	*lactamica*	BT17759V1C	UK	2019	997	No
147908	*lactamica*	BT17893V1C	UK	2019	736	No
147910	*lactamica*	BT17946V1C	UK	2019	4	No
147912	*lactamica*	BT22331V1C	UK	2019	706	No
147914	*lactamica*	BT22356V1C	UK	2019	2341	No
147915	*lactamica*	BT22357V1C	UK	2019	744	No
147916	*lactamica*	BT22370V1C	UK	2019	725	No
147937	*lactamica*	BT22711V1C	UK	2019	2342	No
147945	*lactamica*	BT25412V1C	UK	2019	619	No
147948	*lactamica*	BT25526V1C	UK	2019	706	No
147949	*lactamica*	BT25539V1C	UK	2019	725	No
147954	*lactamica*	BT25576V1C	UK	2019	725	No
147957	*lactamica*	BT25593V1C	UK	2019	997	No
147960	*lactamica*	BT25618V1C	UK	2019	706	No
147963	*lactamica*	BT25670V1C	UK	2019	195	No
147964	*lactamica*	BT25672V1C	UK	2019	476	No
147969	*lactamica*	BT25731V1C	UK	2019	476	No
147978	*lactamica*	BT25947V1C	UK	2019	472	No
148000	*lactamica*	BT27834V1C	UK	2019	997	No
148001	*lactamica*	BT30404V1C	UK	2018	195	No
148002	*lactamica*	BT38465V1C	UK	2019	706	No
148005	*lactamica*	BT38677V1C	UK	2019	988	No
148010	*lactamica*	BT38755V1C	UK	2019	2027	No
148011	*lactamica*	BT38758V1C	UK	2019	562	No
148012	*lactamica*	BT39986V1C	UK	2019	744	No
148015	*lactamica*	BT40036V1C	UK	2019	4	No
148018	*lactamica*	BT50870V1C	UK	2019	725	No
148020	*lactamica*	BT50891V1C	UK	2019	4	No
148028	*lactamica*	BT51694V1C	UK	2019	472	No
148029	*lactamica*	BT51712V1C	UK	2019	997	No
148030	*lactamica*	BT51739V1C	UK	2019	2349	No
148031	*lactamica*	BT04616V1C1	UK	Unknown	2350	No
148618	*lactamica*	BT19199V1C	UK	2019	4	No
148621	*lactamica*	BT27753V1C	UK	2019	532	No
148628	*lactamica*	BT27945V1C	UK	2019	725	No
148633	*lactamica*	BT27975V1C	UK	2019	2427	No
148635	*lactamica*	BT27986V1C	UK	2019	NA	No
148642	*lactamica*	BT31180V1C	UK	2019	1582	No
148658	*lactamica*	BT31725V1C	UK	2019	2433	No
148660	*lactamica*	BT31836V1C	UK	2019	195	No
148662	*lactamica*	BT31870V1C	UK	2019	2118	No
148667	*lactamica*	BT32039V1C	UK	2019	2435	No
148669	*lactamica*	BT32096V1C	UK	2019	2436	No
148677	*lactamica*	BT33731V1C	UK	2019	2041	No
148679	*lactamica*	BT33743V1C	UK	2019	706	No
148681	*lactamica*	BT33822V1C	UK	2019	4	No
148688	*lactamica*	BT34066V1C	UK	2019	195	No
148690	*lactamica*	BT34075V1C	UK	2019	2039	No
148692	*lactamica*	BT34091V1C	UK	2019	4	No
148697	*lactamica*	BT38850V1C	UK	2019	2427	No
148704	*lactamica*	BT51082V3C	UK	2018	736	No
148718	*lactamica*	BT51810V1C	UK	2019	997	No
148719	*lactamica*	BT51818V1C	UK	2019	997	No
148732	*lactamica*	BT51940V1C	UK	2019	NA	No
148735	*lactamica*	BT51968V1C	UK	2019	4	No
168187	*lactamica*	LN202134	China	2021	2629	No
168324	*lactamica*	SCLN2022083	China	2022	725	No
169283	*lactamica*	RT3	China	2024	2720	No
169284	*lactamica*	RT5	China	2024	706	No
178191	*lactamica*	5518	Unknown	Unknown	2778	No
178192	*lactamica*	5519	Unknown	Unknown	2779	No
178193	*lactamica*	6139	Unknown	Unknown	2779	No

178197	*lactamica*	6940	Unknown	Unknown	2779		No
178198	*lactamica*	6941	Unknown	Unknown	2780		No
3416	*cinerea*	OX9931913	UK	1999	1813		No
14731	*cinerea*	ATCC_14685	Unknown	Unknown	1730		No
21041	*cinerea*	CCUG_53043	Sweden	2006	1814		No
26871	*cinerea*	12008_2012	Ireland	2012	1815		No
26876	*cinerea*	12013_2012	Ireland	2012	650		No
36161	*cinerea*	12040_2014	Ireland	2014	1816		No
38978	*cinerea*	LNP18981	France	Unknown	1817		No
38984	*cinerea*	LNP26654	France	Unknown	1818		No
38986	*cinerea*	LNP23132	France	Unknown	1819		No
39717	*cinerea*	LNP20692	France	2003	1821		No
39721	*cinerea*	LNP981	France	1979	1822		No
39724	*cinerea*	LNP23872	France	2007	1823		No
42057	*cinerea*	LNP28127	France	2015	651		No
42058	*cinerea*	LNP28155	France	2015	650		No
42059	*cinerea*	LNP28158	France	2015	1820		No
42061	*cinerea*	LNP28181	France	2015	747		No
42062	*cinerea*	LNP28272	France	2015	479		No
42421	*cinerea*	LNP28525	France	2016	1824		No
42462	*cinerea*	M16_240153	UK	2016	1913		No
42471	*cinerea*	M15_240769	UK	2015	1904		No
42503	*cinerea*	M16_240038	UK	2016	1914		No
44493	*cinerea*	OX40632	UK	2015	747		No
44631	*cinerea*	OX42181	UK	2015	649	40	No
47345	*cinerea*	M16_240660	UK	2016	1830		No
49341	*cinerea*	CCUG_346_T	Unknown	Unknown	1825		No
49342	*cinerea*	CCUG_5746	Sweden	1977	1826		No
49343	*cinerea*	CCUG_25879	Sweden	1989	1827		No
49344	*cinerea*	CCUG_27178_A	Unknown	1983	1828		No
50859	*cinerea*	16-530	Sweden	2016	1830		No
63425	*cinerea*	M19_240435	UK	2019	1817		No
63426	*cinerea*	M19_240436	UK	2019	1831		No
63428	*cinerea*	M19_240438	UK	2019	1832		No
63430	*cinerea*	M19_240440	UK	2019	1833		No
63431	*cinerea*	M19_240441	UK	2019	1834		No
63432	*cinerea*	M19_240442	UK	2019	649	40	No
63433	*cinerea*	M19_240443	UK	2019	1835		No
72035	*cinerea*	SMG-20-140	UK	2020	2045		No
84201	*cinerea*	Nei0028	China	2014	1565		No
93282	*cinerea*	McBar-2122	Sweden	Unknown	649	40	No
103530	*cinerea*	AK-2152	Greece	2020	1836		No
105711	*cinerea*	Nei0798	China	2019	2212		No
105712	*cinerea*	Nei1004	China	2019	2213		No
122993	*cinerea*	19061666/1	Belgium	2019	798		No
123009	*cinerea*	19091461/1	Belgium	2019	1837		No
123040	*cinerea*	Co000769/3	Belgium	2020	1838		No
123056	*cinerea*	Co000776/4	Belgium	2020	1839		No
123065	*cinerea*	Co000782/1	Belgium	2020	1840		No
127318	*cinerea*	H11-RESP-003	Spain	2018	1826		No
140841	*cinerea*	H04-RESP-016	Spain	2023	2298		No
148086	*cinerea*	H15-RESP-030	Spain	2024	650		No
167250	*cinerea*	0058/15	Czech Republic	2015	2604		No
167253	*cinerea*	0072/19	Czech Republic	2019	2605		No
167255	*cinerea*	0040/22	Czech Republic	2022	2607		No
167257	*cinerea*	0034/23	Czech Republic	2023	2609		No
167260	*cinerea*	0038/24	Czech Republic	2024	2611		No
167261	*cinerea*	0051/24	Czech Republic	2024	2612		No
171017	*cinerea*	0090/16	Unknown	2016	1830		No
171018	*cinerea*	0040/18	Czech Republic	2018	2740		No
171024	*cinerea*	0087/24	Czech Republic	2024	2611		No
171028	*cinerea*	0042/25	Czech Republic	2025	1852		No
178196	*cinerea*	6148	Unknown	Unknown	1730		No
14730	*polysaccharea*	ATCC_43768	Belgium	Unknown	785		No
21047	*polysaccharea*	CCUG_4790	Unknown	Unknown	77		No
36140	*polysaccharea*	12015_2014	Ireland	2014	NA		No
36142	*polysaccharea*	12017_2014	Ireland	2014	726		No
36148	*polysaccharea*	12024_2014	Ireland	2014	1888		No

36153	*polysaccharea*	12030_2014	Ireland	2014	1889	No
36167	*polysaccharea*	12046_2014	Ireland	2014	574	No
38980	*polysaccharea*	LNP22706	France	Unknown	1902	No
38981	*polysaccharea*	LNP24574	France	Unknown	1903	No
40248	*polysaccharea*	LNP28315	France	2015	1907	No
41474	*polysaccharea*	M15_240880	UK	2015	1908	No
41528	*polysaccharea*	M15_240955	UK	2015	442	No
41652	*polysaccharea*	2748	Italy	2015	732	No
42468	*polysaccharea*	M16_240183	UK	2016	712	No
42472	*polysaccharea*	M15_240827	UK	2015	713	No
43335	*polysaccharea*	01_00098_XS1_1	Mali	2010	808	No
43413	*polysaccharea*	01_07665_XS1_1	Mali	2010	809	No
43415	*polysaccharea*	01_03967_XS1_1	Mali	2010	810	No
43416	*polysaccharea*	01_07343_XS1_1	Mali	2010	811	No
43417	*polysaccharea*	01_08009_XS1_1	Mali	2010	812	No
43418	*polysaccharea*	01_13553_XS3_1	Mali	2010	813	No
43419	*polysaccharea*	01_13554_XS3_1	Mali	2010	813	No
43420	*polysaccharea*	01_13564_XS3_1	Mali	2010	511	No
43987	*polysaccharea*	LNP28609	France	2016	522	No
44084	*polysaccharea*	ST41786	UK	2015	785	No
44102	*polysaccharea*	BR40761B	UK	2014	80	No
44630	*polysaccharea*	OX42150	England	2015	573	No
44655	*polysaccharea*	52189	South Africa	2017	1950	No
44837	*polysaccharea*	M16_240285	UK	2016	573	No
45356	*polysaccharea*	LNP28667	France	2016	713	No
46136	*polysaccharea*	G97_6093	The Gambia	1997	1951	No
46143	*polysaccharea*	G97_5907	The Gambia	1997	1952	No
46173	*polysaccharea*	G97_2571	The Gambia	1997	1952	No
46186	*polysaccharea*	G97_1651	The Gambia	1997	1952	No
46196	*polysaccharea*	G97_1162	The Gambia	1997	809	No
46197	*polysaccharea*	G97_1151	The Gambia	1997	828	No
46199	*polysaccharea*	G97_1072	The Gambia	1997	829	No
46200	*polysaccharea*	G97_1061	The Gambia	1997	1954	No
46409	*polysaccharea*	M-080	Malawi	Unknown	1960	No
46417	*polysaccharea*	M-104	Malawi	Unknown	NA	No
46418	*polysaccharea*	M-104A	Malawi	Unknown	NA	No
46428	*polysaccharea*	M-198	Malawi	Unknown	1962	No
46437	*polysaccharea*	M-223	Malawi	Unknown	1963	No
46449	*polysaccharea*	M-264	Malawi	Unknown	1965	No
46451	*polysaccharea*	M-286	Malawi	Unknown	1965	No
46462	*polysaccharea*	M-442	Malawi	Unknown	NA	No
46467	*polysaccharea*	M-571	Malawi	Unknown	NA	No
46475	*polysaccharea*	M-856	Malawi	Unknown	1970	No
46727	*polysaccharea*	PL40288	UK	2015	573	No
49356	*polysaccharea*	CCUG_18031	Germany	1984	785	No
49357	*polysaccharea*	CCUG_24845	Unknown	Unknown	80	No
49359	*polysaccharea*	CCUG_27182	Unknown	Unknown	76	No
53400	*polysaccharea*	M17_240155	Unknown	Unknown	NA	No
58873	*polysaccharea*	LNP29576	France	2017	713	No
59953	*polysaccharea*	AG2-156	South Africa	2017	2010	No
63424	*polysaccharea*	M19_240434	UK	2019	2018	No
63427	*polysaccharea*	M19_240437	UK	2019	2019	No
63429	*polysaccharea*	M19_240439	UK	2019	2019	No
63842	*polysaccharea*	BT27107V1C	England	2018	1522	No
63962	*polysaccharea*	BT39793V1C	England	2019	573	No
71450	*polysaccharea*	Mcbar-1617	Sweden	Unknown	NA	No
72290	*polysaccharea*	M18_240317	UK	2019	NA	No
75034	*polysaccharea*	P206399499U	Australia	2020	713	No
77854	*polysaccharea*	SMG-20-1074	UK	2019	1875	No
82539	*polysaccharea*	P206414321C	Australia	2020	2080	No
84258	*polysaccharea*	Nei0367	China	2018	2098	No
85853	*polysaccharea*	EF-190	Greece	2017	NA	No
88571	*polysaccharea*	BT24866V1C	UK	2018	650	No
89663	*polysaccharea*	Nmen72	Spain	2019	2141	No
91772	*polysaccharea*	3_MEX	Mexico	2019	NA	No
95834	*polysaccharea*	LNP31683	France	2020	2175	No
120714	*polysaccharea*	BT00702V3C	UK	2020	573	No
123078	*polysaccharea*	Co000790/1	Belgium	2020	2251	No

127276	*polysaccharea*	Nmen49	Spain	2019	2141	No
127317	*polysaccharea*	H11-RESP-002	Spain	2022	1592	No
127328	*polysaccharea*	H14-RESP-013	Spain	2014	2254	No
136063	*polysaccharea*	SMG-23-1917	UK	2023	2297	No
140842	*polysaccharea*	H04-RESP-017	Spain	2023	2333	No
143426	*polysaccharea*	NML2023-133	Canada	2023	1849	No
144078	*polysaccharea*	BT01068V1C	UK	2019	573	No
147976	*polysaccharea*	BT25943V1C	UK	2019	1903	No
150142	*polysaccharea*	IE23Nm35	Ireland	2023	712	No
154711	*polysaccharea*	NML2024-118	Canada	2024	712	No
161271	*polysaccharea*	NML2024-131	Canada	2024	713	No
161331	*polysaccharea*	M18661	USA	Unknown	785	No
162584	*polysaccharea*	NML2024-155	Canada	2024	2558	No
162585	*polysaccharea*	NML2024-156	Canada	2024	2558	No
164715	*polysaccharea*	LNP35267	France	2024	76	No
167251	*polysaccharea*	0042/17	Czech Republic	2017	712	No
167252	*polysaccharea*	0044/18	Czech Republic	2018	712	No
167254	*polysaccharea*	0018/22	Czech Republic	2022	2606	No
167256	*polysaccharea*	0046/22	Czech Republic	2022	2608	No
167258	*polysaccharea*	0053/23	Czech Republic	2023	712	No
171020	*polysaccharea*	0076/19	Czech Republic	2019	1082	No
171021	*polysaccharea*	0077/19	Czech Republic	2019	1082	No
171022	*polysaccharea*	0042/24	Czech Republic	2024	712	No
171025	*polysaccharea*	0007/25	Czech Republic	2025	2741	No
171026	*polysaccharea*	0031/25	Czech Republic	2025	712	No
171027	*polysaccharea*	0032/25	Czech Republic	2025	NA	No
171029	*polysaccharea*	0048/25	Czech Republic	2025	76	No
171030	*polysaccharea*	0050/25	Czech Republic	2025	712	No
178189	*polysaccharea*	0079/25	Czech Republic	2025	77	No
178227	*polysaccharea*	PMS5061	Ghana	2023	2782	No
6415	*subflava*	OX9931485	UK	1999	NA	No
14732	*subflava*	NRL30031	Unknown	Unknown	NA	No
14733	*subflava*	NJ9703	Unknown	Unknown	1479	No
21060	*subflava*	C102	Unknown	Unknown	1878	No
21063	*subflava*	CCUG 24841	Unknown	Unknown	NA	No
21064	*subflava*	CCUG 24844	Unknown	Unknown	NA	No
26870	*subflava*	12007_2012	Ireland	2012	1883	No
38976	*subflava*	LNP17345	France	Unknown	1901	No
38979	*subflava*	LNP19085	France	Unknown	NA	No
39722	*subflava*	LNP26332	France	2011	NA	No
39723	*subflava*	LNP26308	France	2011	1905	No
40261	*subflava*	LNP28331	France	2015	NA	No
40270	*subflava*	LNP28340	France	2015	NA	No
42060	*subflava*	LNP28165	France	2015	NA	No
43570	*subflava*	BR40103	UK	2014	739	No
43658	*subflava*	BR41637	UK	2015	743	No
43740	*subflava*	BR40599	UK	2014	759	No
43741	*subflava*	BR40608	UK	2014	761	No
43793	*subflava*	BR40664	UK	2014	777	No
43794	*subflava*	BR41470	UK	2015	783	No
44065	*subflava*	ST41782	UK	2015	784	No
44613	*subflava*	OX41971	UK	2015	786	No
44615	*subflava*	OX42005	UK	2015	787	No
44616	*subflava*	OX42006	UK	2015	788	No
44620	*subflava*	OX42049	UK	2015	789	No
46032	*subflava*	12026-15	Ireland	2015	793	No
46033	*subflava*	12027-15A	Ireland	2015	843	No
46034	*subflava*	12029-15A	Ireland	2015	843	No
46079	*subflava*	T-20455-10	Ireland	2015	NA	No
46268	*subflava*	583_NLAC	USA	Unknown	NA	No
46269	*subflava*	595_NLAC	USA	Unknown	NA	No
46753	*subflava*	C6A	Malaysia	2012	NA	No
49346	*subflava*	CCUG 4788	Unknown	Unknown	NA	No
49347	*subflava*	CCUG 29761	Sweden	1992	NA	No
49348	*subflava*	CCUG 17913 T	USA	Unknown	NA	No
49349	*subflava*	CCUG 806	Unknown	Unknown	NA	No
49353	*subflava*	CCUG 7826	Sweden	1979	802	No
49362	*subflava*	CCUG 24918	Sweden	1989	1876	No

49363	*subflava*	CCUG 23930 T	Unknown	Unknown	NA	No
49364	*subflava*	CCUG 800	Unknown	Unknown	NA	No
49365	*subflava*	CCUG 801	Unknown	Unknown	NA	No
49366	*subflava*	CCUG 24960	Unknown	Unknown	NA	No
49367	*subflava*	CCUG 25198	Sweden	1989	NA	No
49373	*subflava*	SK114	Unknown	Unknown	NA	No
49571	*subflava*	CA42438	UK	2015	791	No
49863	*subflava*	PL40548	UK	2015	792	No
50889	*subflava*	MT40035	UK	2015	793	No
50940	*subflava*	PL42224	UK	2015	794	No
52432	*subflava*	LNP28992	France	2017	NA	No
61329	*subflava*	20925-23	UK	2018	NA	No
82618	*subflava*	ExNm709	Australia	Unknown	NA	No
82623	*subflava*	ExNm715	Australia	Unknown	NA	No
82624	*subflava*	ExNm716	Australia	Unknown	1690	No
82626	*subflava*	ExNm719	Australia	Unknown	1234	No
82629	*subflava*	ExNm725	Australia	Unknown	NA	No
82631	*subflava*	ExNm727	Australia	Unknown	1545	No
84263	*subflava*	Nei0437	China	2014	NA	No
88486	*subflava*	BT18757V1C	UK	2018	991	No
88688	*subflava*	BT37822V1C	UK	2018	793	No
91511	*subflava*	CNF	Italy	2013	NA	No
92876	*subflava*	Nei0438	China	2014	NA	No
111367	*subflava*	SC34	Saudi Arabia	2019	1763	No
115603	*subflava*	BT37503V3C	UK	2019	1497	No
120677	*subflava*	BT00069V3C	UK	2019	1558	No
120837	*subflava*	BT25333V3C	UK	2020	1561	No
122455	*subflava*	BT24957V3C	UK	2019	1509	No
122966	*subflava*	19040900/1	Belgium	2019	NA	No
122968	*subflava*	19041324/2	Belgium	2019	NA	No
122969	*subflava*	19041762/1	Belgium	2019	1534	No
122971	*subflava*	19041780/1	Belgium	2019	1535	No
122972	*subflava*	19042150/1	Belgium	2019	NA	No
122974	*subflava*	19042756/1	Belgium	2019	1534	No
122977	*subflava*	19043201/1.1	Belgium	2019	NA	No
122979	*subflava*	19043274/3	Belgium	2019	NA	No
122980	*subflava*	19050474/1	Belgium	2019	NA	No
122981	*subflava*	19050514/1	Belgium	2019	NA	No
122982	*subflava*	19050949/1	Belgium	2019	NA	No
122985	*subflava*	19051904/2	Belgium	2019	NA	No
122986	*subflava*	19052394/1	Belgium	2019	NA	No
122991	*subflava*	19060089/1	Belgium	2019	1536	No
122996	*subflava*	19062994/1	Belgium	2019	1537	No
122997	*subflava*	19063007/1	Belgium	2019	NA	No
122998	*subflava*	19070015/1	Belgium	2019	NA	No
122999	*subflava*	19070019/1	Belgium	2019	1538	No
123001	*subflava*	19070047/1	Belgium	2019	2250	No
123003	*subflava*	19070909/1	Belgium	2019	NA	No
123004	*subflava*	19072168/1	Belgium	2019	NA	No
123005	*subflava*	19081774/1	Belgium	2019	786	No
123007	*subflava*	19082621/1	Belgium	2019	1539	No
123010	*subflava*	19091480/1	Belgium	2019	1540	No
123012	*subflava*	19092429/1	Belgium	2019	NA	No
123015	*subflava*	19103457/2	Belgium	2019	802	No
123017	*subflava*	19110394/2	Belgium	2019	1541	No
123018	*subflava*	19111422/1	Belgium	2019	1542	No
123020	*subflava*	19111441/1	Belgium	2019	1541	No
123022	*subflava*	19113052/1	Belgium	2019	1543	No
123023	*subflava*	Co000759/1	Belgium	2020	1544	No
123024	*subflava*	Co000760/1	Belgium	2020	NA	No
123026	*subflava*	Co000761/2	Belgium	2020	NA	No
123028	*subflava*	Co000762/3	Belgium	2020	1545	No
123030	*subflava*	Co000763/2	Belgium	2020	1546	No
123031	*subflava*	Co000764/1	Belgium	2020	1547	No
123033	*subflava*	Co000765/2	Belgium	2020	1566	No
123034	*subflava*	Co000766/1	Belgium	2020	1479	No
123036	*subflava*	Co000767/1	Belgium	2020	NA	No
123038	*subflava*	Co000768/1	Belgium	2020	NA	No

123039	*subflava*	Co000769/1	Belgium	2020	NA	No
123042	*subflava*	Co000770/2	Belgium	2020	991	No
123045	*subflava*	Co000771/2	Belgium	2020	1549	No
123047	*subflava*	Co000772/1	Belgium	2020	1550	No
123049	*subflava*	Co000773/1	Belgium	2020	NA	No
123052	*subflava*	Co000774/2	Belgium	2020	NA	No
123053	*subflava*	Co000775/1	Belgium	2020	NA	No
123055	*subflava*	Co000776/2	Belgium	2020	1479	No
123057	*subflava*	Co000777/1	Belgium	2020	1551	No
123058	*subflava*	Co000778/1	Belgium	2020	1552	No
123062	*subflava*	Co000780/4	Belgium	2020	NA	No
123064	*subflava*	Co000781/1	Belgium	2020	1553	No
123066	*subflava*	Co000782/2	Belgium	2020	NA	No
123067	*subflava*	Co000783/1	Belgium	2020	1554	No
123070	*subflava*	Co000784/1	Belgium	2020	1555	No
123071	*subflava*	Co000785/1	Belgium	2020	NA	No
123072	*subflava*	Co000786/1	Belgium	2020	1556	No
123073	*subflava*	Co000787/1	Belgium	2020	1561	No
123076	*subflava*	Co000788/2	Belgium	2020	NA	No
123077	*subflava*	Co000789/1	Belgium	2020	1557	No
123079	*subflava*	Co000790/2	Belgium	2020	NA	No
143425	*subflava*	NML2023-131	Canada	2023	1557	No
144064	*subflava*	BT00870V1C	UK	2019	NA	No
144079	*subflava*	BT01077V1C	UK	2019	1867	No
144080	*subflava*	BT01083V1C	UK	2019	1858	No
144083	*subflava*	BT01117V1C	UK	2019	1859	No
144192	*subflava*	BT27478V1C	UK	2019	1862	No
144193	*subflava*	BT27478V1C2	UK	2019	1862	No
144217	*subflava*	BT50802V1C	UK	2019	NA	No
144220	*subflava*	BT50832V1C	UK	2019	1869	No
144224	*subflava*	BT50902V1C	UK	2019	NA	No
144225	*subflava*	BT50910V1C	UK	2019	1868	No
144226	*subflava*	BT50911V1C	UK	2019	1866	No
147839	*subflava*	BT01069V1C	UK	2019	NA	No
147840	*subflava*	BT01115V1C	UK	2019	2334	No
147841	*subflava*	BT01121V1C	UK	2019	NA	No
147843	*subflava*	BT01180V1C	UK	2019	2336	No
147950	*subflava*	BT25553V1C	UK	2019	2369	No
147958	*subflava*	BT25602V1C	UK	2019	2343	No
147959	*subflava*	BT25615V1C	UK	2019	2368	No
147961	*subflava*	BT25659V1C	UK	2019	NA	No
147965	*subflava*	BT25678V1C	UK	2019	NA	No
147966	*subflava*	BT25699V1C	UK	2019	2344	No
147967	*subflava*	BT25714V1C	UK	2019	2345	No
147972	*subflava*	BT25885V1C	UK	2019	NA	No
147973	*subflava*	BT25899V1C	UK	2019	2346	No
147980	*subflava*	BT25988V1C	UK	2019	2367	No
147985	*subflava*	BT26040V1C	UK	2019	2347	No
147986	*subflava*	BT26041V1C	UK	2019	1556	No
148019	*subflava*	BT50875V1C	UK	2019	802	No
148620	*subflava*	BT19213V1C	UK	2019	NA	No
148631	*subflava*	BT27955V1C	UK	2019	2426	No
148643	*subflava*	BT31455V1C	UK	2019	2429	No
148644	*subflava*	BT31456V1C	UK	2019	NA	No
148656	*subflava*	BT31681V1C	UK	2019	2431	No
148657	*subflava*	BT31721V1C	UK	2019	2432	No
148659	*subflava*	BT31829V1C	UK	2019	2434	No
148664	*subflava*	BT31981V1C	UK	2019	NA	No
148666	*subflava*	BT32028V1C	UK	2019	2346	No
148675	*subflava*	BT33690V1C	UK	2019	2437	No
148680	*subflava*	BT33757V1C	UK	2019	NA	No
148687	*subflava*	BT33975V1C	UK	2019	2438	No
148708	*subflava*	BT51446V3C	UK	2018	NA	No
148722	*subflava*	BT51862V1C	UK	2019	NA	No
148723	*subflava*	BT51863V1C	UK	2019	NA	No
148724	*subflava*	BT51864V1C	UK	2019	802	No
148725	*subflava*	BT51869V1C	UK	2019	777	No
148726	*subflava*	BT51876V1C	UK	2019	2415	No

148727	*subflava*	BT51885V1C	UK	2019	NA	No
148728	*subflava*	BT51888V1C	UK	2019	NA	No
148731	*subflava*	BT51919V1C	UK	2019	1557	No
148733	*subflava*	BT51947V1C	UK	2019	802	No
148736	*subflava*	BT51982V1C	UK	2019	2382	No
161269	*subflava*	NML2024-133	Canada	2024	NA	No
161715	*subflava*	FDAARGOS_758	USA	Unknown	1878	No
161716	*subflava*	BgEED22	USA	2021	NA	No
167262	*subflava*	414	Czech Republic	Unknown	NA	No
167264	*subflava*	2151	Czech Republic	Unknown	NA	No
170037	*subflava*	NML2025-094	Canada	2025	NA	No
178190	*subflava*	0665	Unknown	Unknown	NA	No
178201	*subflava*	7440	Unknown	Unknown	NA	No
2863	*mucosa*	ATCC 29256	Unknown	Unknown	NA	No
3565	*mucosa*	ATCC 25996	Unknown	Unknown	NA	No
5197	*mucosa*	ATCC 9913	USA	1952	NA	No
5204	*mucosa*	ATCC 19243	USA	1973	NA	No
5354	*mucosa*	ATCC 19696	France	1959	NA	No
21043	*mucosa*	CCUG 26474	UK	Unknown	NA	No
21061	*mucosa*	CCUG 17327	Sweden	1985	NA	No
26577	*mucosa*	GT4A_CT1	Unknown	Unknown	NA	No
27632	*mucosa*	CCUG 805	Unknown	Unknown	NA	No
37778	*mucosa*	M14 240642	UK	2014	NA	No
38974	*mucosa*	LNP16475	France	Unknown	NA	No
38977	*mucosa*	LNP17862	France	Unknown	NA	No
39715	*mucosa*	LNP16858	France	1999	NA	No
45355	*mucosa*	LNP28655	France	2016	NA	No
46114	*mucosa*	LNP28729	France	2016	NA	No
46270	*mucosa*	919_NLAC	USA	Unknown	NA	No
49351	*mucosa*	CCUG 41451 T	Unknown	Unknown	NA	No
49352	*mucosa*	CCUG 12106	Sweden	1982	NA	No
49354	*mucosa*	CCUG 32036	Sweden	1993	NA	No
49355	*mucosa*	CCUG 32112	Sweden	1993	NA	No
49360	*mucosa*	CCUG 431	Unknown	Unknown	NA	No
49361	*mucosa*	CCUG 24847	Unknown	Unknown	NA	No
82612	*mucosa*	EXNM702	Australia	Unknown	NA	No
82613	*mucosa*	ExNm703	Australia	Unknown	NA	No
82617	*mucosa*	ExNm708	Australia	Unknown	NA	No
82619	*mucosa*	ExNm711	Australia	Unknown	NA	No
82621	*mucosa*	ExNm713	Australia	Unknown	NA	No
111346	*mucosa*	SC13	Saudi Arabia	2019	NA	No
122964	*mucosa*	19040874/1	Belgium	2019	NA	No
122970	*mucosa*	19041762/2	Belgium	2019	NA	No
122984	*mucosa*	19051904/1	Belgium	2019	NA	No
122987	*mucosa*	19052394/2	Belgium	2019	NA	No
122994	*mucosa*	19062968/1	Belgium	2019	NA	No
123006	*mucosa*	19081774/2	Belgium	2019	NA	No
123019	*mucosa*	19111422/4	Belgium	2019	NA	No
123043	*mucosa*	Co000770/5	Belgium	2020	NA	No
123046	*mucosa*	Co000771/4	Belgium	2020	NA	No
123048	*mucosa*	Co000772/3	Belgium	2020	NA	No
123050	*mucosa*	Co000773/3	Belgium	2020	NA	No
123059	*mucosa*	Co000779/1	Belgium	2020	NA	No
123068	*mucosa*	Co000783/3	Belgium	2020	NA	No
123074	*mucosa*	Co000787/3	Belgium	2020	NA	No
123075	*mucosa*	Co000788/1	Belgium	2020	NA	No
123931	*mucosa*	IE22Nm14	Ireland	2022	NA	No
167265	*mucosa*	3834	Czech Republic	Unknown	NA	No
171016	*mucosa*	0041/15	Czech Republic	2015	NA	No
178194	*mucosa*	6143	Unknown	Unknown	NA	No
178195	*mucosa*	6144	Unknown	Unknown	NA	No
39718	*bergeri*	LNP23737	France	2006	1886	No
41220	*bergeri*	LNP28396	France	2016	77	No
42909	*bergeri*	02_11162_XS2_1	Mali	2011	1918	No
43020	*bergeri*	02_11069_XS2_1	Mali	2011	1918	No
43021	*bergeri*	02_11081_XS2_1	Mali	2011	1918	No
43022	*bergeri*	02_11161_XS2_1	Mali	2011	1918	No
43023	*bergeri*	02_11615_XS2_1	Mali	2011	1918	No

43024	*bergeri*	02_11164_XS2_1	Mali	2011	1918	No
43025	*bergeri*	02_11253_XS2_1	Mali	2011	1918	No
43026	*bergeri*	02_11666_XS2_1	Mali	2011	NA	No
43027	*bergeri*	02_11761_XS2_1	Mali	2011	1918	No
43028	*bergeri*	02_11322_XS2_1	Mali	2011	NA	No
43029	*bergeri*	02_11327_XS2_1	Mali	2011	NA	No
43030	*bergeri*	02_11362_XS2_1	Mali	2011	1918	No
43031	*bergeri*	02_11346_XS2_1	Mali	2011	1918	No
43032	*bergeri*	02_11474_XS2_1	Mali	2011	1918	No
43033	*bergeri*	02_11433_XS2_1	Mali	2011	1918	No
43034	*bergeri*	02_13924_XS3_1	Mali	2012	1918	No
43035	*bergeri*	02_13976_XS3_1	Mali	2012	1918	No
43036	*bergeri*	02_14067_XS3_1	Mali	2012	1918	No
43037	*bergeri*	02_13995_XS3_1	Mali	2012	1918	No
43038	*bergeri*	02_14121_XS3_1	Mali	2012	1918	No
43039	*bergeri*	01_13562_XS3_1	Mali	2012	1918	No
43040	*bergeri*	01_13695_XS3_1	Mali	2012	540	No
43041	*bergeri*	01_13712_XS3_1	Mali	2012	1918	No
43042	*bergeri*	01_12742_XS3_1	Mali	2012	NA	No
43043	*bergeri*	01_13770_XS3_1	Mali	2012	1918	No
43421	*bergeri*	01_00119_XS1_1	Mali	2010	1947	No
43422	*bergeri*	01_03638_XS1_1	Mali	2010	1948	No
43423	*bergeri*	01_03957_XS1_1	Mali	2010	NA	No
43424	*bergeri*	02_01070_XS1_1	Mali	2010	1918	No
43426	*bergeri*	02_01287_XS1_1	Mali	2010	1918	No
43427	*bergeri*	02_07958_XS1_1	Mali	2010	1918	No
43428	*bergeri*	01_00007_XS1_1	Mali	2010	1947	No
43429	*bergeri*	01_03581_XS1_1	Mali	2010	NA	No
43430	*bergeri*	01_07472_XS1_1	Mali	2010	1918	No
43431	*bergeri*	01_07795_XS1_1	Mali	2010	1918	No
43432	*bergeri*	02_01039_XS1_1	Mali	2010	1918	No
46230	*bergeri*	M_676M	Malawi	2006	754	No
46400	*bergeri*	M-037	Malawi	Unknown	1958	No
46401	*bergeri*	M-038	Malawi	Unknown	1958	No
46404	*bergeri*	M-052	Malawi	Unknown	72	No
46406	*bergeri*	M-065	Malawi	Unknown	1959	No
46421	*bergeri*	M-129	Malawi	Unknown	1958	No
46422	*bergeri*	M-130	Malawi	Unknown	1958	No
46424	*bergeri*	M-161	Malawi	Unknown	1958	No
46427	*bergeri*	M-190	Malawi	Unknown	1958	No
46434	*bergeri*	M-214	Malawi	Unknown	584	No
46443	*bergeri*	M-250	Malawi	Unknown	1958	No
46444	*bergeri*	M-252	Malawi	Unknown	1958	No
46445	*bergeri*	M-256C	Malawi	Unknown	1964	No
46448	*bergeri*	M-262	Malawi	Unknown	1958	No
46450	*bergeri*	M-274	Malawi	Unknown	1966	No
46452	*bergeri*	M-290	Malawi	Unknown	1958	No
46459	*bergeri*	M-357	Malawi	Unknown	1968	No
46460	*bergeri*	M-366	Malawi	Unknown	1958	No
46469	*bergeri*	M-718M	Malawi	Unknown	1958	No
46470	*bergeri*	M-760	Malawi	Unknown	NA	No
49259	*bergeri*	LNP28180	France	2015	1502	No
49339	*bergeri*	15883	Germany	1984	1873	No
58874	*bergeri*	LNP_MET	France	2017	NA	No
151003	*bergeri*	0016/24	Czech Republic	2024	2465	No
167259	*bergeri*	0032/24	Czech Republic	2024	2610	No
178225	*bergeri*	PMS4349	Ghana	2023	2781	No
178226	*bergeri*	PMS4821	Ghana	2023	NA	No
178228	*bergeri*	PMS5101	Ghana	2023	2783	No
178229	*bergeri*	PMS5160	Ghana	2023	NA	No
178230	*bergeri*	PMS5594	Ghana	2023	2783	No
178231	*bergeri*	PMS5659	Ghana	2023	2783	No
178232	*bergeri*	PMS5733	Ghana	2023	1984	No
178233	*bergeri*	PMS5734	Ghana	2023	1984	No
178235	*bergeri*	PMS5961	Ghana	2023	2781	No
178237	*bergeri*	PMS6290	Ghana	2023	2781	No
178240	*bergeri*	PMS6338	Ghana	2023	NA	No
178241	*bergeri*	PMS6359	Ghana	2023	NA	No

178243	*bergeri*	PMS6536	Ghana	2023	2785	No
178244	*bergeri*	PMS6701	Ghana	2023	2786	No
178245	*bergeri*	PMS6882	Ghana	2023	2785	No
178246	*bergeri*	PMS6896	Ghana	2023	2783	No
178247	*bergeri*	PMS6912	Ghana	2023	2785	No
178248	*bergeri*	PMS6949	Ghana	2023	2785	No
178249	*bergeri*	PMS6969	Ghana	2023	NA	No
178251	*bergeri*	PMS7380	Ghana	2023	NA	No
178253	*bergeri*	WA/SW/010	Ghana	2024	2785	No
178254	*bergeri*	WA/WE/043	Ghana	2024	2787	No

* NA (allele not assigned)

Supplementary Table 5. *Neisseria meningitidis* and commensal *Neisseria* spp. used in this study

PubMLST ID	Species (Neisseria)	Isolate	Country	Year	MtrE (NEIS1632) allele	Reference
698	*meningitidis*	Fam18	USA	1983	1	Feavers et al (1999)
1038	*meningitidis*	8013	France	1989	16	Rusniok et al. (2009)
240	*meningitidis*	MC58	UK	1983	3	Tettelin et al. (2005)
34542	*meningitidis*	NZ 98/254	New Zealand	1998	19	Findlow et al. (2006)
1773	*lactamica*	042-12	UK	1998	-	Bennett et al. (2005)
1775	*lactamica*	047-24	UK	1999	-	Bennett et al. (2005)
1781	*lactamica*	004-24	UK	1998	-	Bennett et al. (2005)
1793	*lactamica*	005-24	UK	1998	-	Bennett et al. (2005)
8851	*lactamica*	020-06	UK	1997	4	Bennett et al. (2005)
49342	*cinerea*	5746	Sweden	1977	1826	Bennett et al. (2012)
49343	*cinerea*	CCUG 25879	Sweden	1989	1827	Bennett et al. (2012)
49344	*cinerea*	CCUG 27178A	Unknown	1983	1828	Bennett et al. (2012)
21041	*cinerea*	CCUG 53043	Sweden	2006	1814	Bennett et al. (2013)
49341	*cinerea*	CCUG 346T	Unknown	Unknown	1825	Bennett et al. (2012)
49356	*polysaccharea*	CCUG 18031	Germany	1984	785	Bennett et al. (2012)
49358	*polysaccharea*	CCUG 24846	Unknown	Unknown	1875	Bennett et al. (2012)
49359	*polysaccharea*	CCUG 27182	Unknown	Unknown	76	Bennett et al. (2012)
49357	*polysaccharea*	CCUG 24845	Unknown	Unknown	80	Bennett et al. (2012)
49339	*bergeri*	15883	Germany	1984	1873	Bennett et al. (2012)

Supplementary Table 6. Presence or absence of IgAP in *N. meningitidis* and commensal *Neisseria* spp.

Species	Presence of NEIS0651 (IgAP)
Neisseria gonorrhoeae	19,865 / 19,865
Neisseria lactamica	0 / 1206
Neisseria polysaccharea	0 / 103
Neisseria cinerea	0 / 61
Neisseria subflava	0 / 187
Neisseria bergeri	0 / 85
Neisseria mucosa	0 / 48

Supplementary Table 7. *N. gonorrhoeae* strains used in this study

PubMLST ID	Isolate	Country	Year	Core genome group	MtrE (NEIS1632) allele	MtrE protein variant	Reference
2855	FA1090 G4	USA	1980	286	245	52	Seifert et al 1994
-	FA1090Δ*mtrE*	-	-	-	-	-	This study
-	FA1090Δ*igAP*	-	-	-	245	52	This study
27228	MS11 E1 p+	USA	1960	288	234	2	Haas, R. et al. 1987
-	MS11Δ*mtrE*	-	-	-	-	-	This study
-	MS11Δ*igAP*	-	-	-	234	2	This study
-	G97687	UK	2018	-	237	1	Eyre, D. W. et al. 2018
36303	NG102	UK	2012	296	238	1	Jongerius, I. et al. 2013
36304	NG104	UK	2012	3	233	5	Jongerius, I. et al. 2013
36305	NG106	UK	2012	3	233	5	Jongerius, I. et al. 2013
36306	NG107	UK	2012	3	233	5	Jongerius, I. et al. 2013
36307	NG109	UK	2012	339	234	2	Jongerius, I. et al. 2013
36308	NG113	UK	2012	159	234	2	Jongerius, I. et al. 2013
36309	NG114	UK	2012	159	234	2	Jongerius, I. et al. 2013
36311	NG118	UK	2012	296	238	1	Jongerius, I. et al. 2013
36312	NG119	UK	2012	314	237	1	Jongerius, I. et al. 2013
36837	NG103	UK	2012	296	238	1	Jongerius, I. et al. 2013
36838	NG105	UK	2012	296	238	1	Jongerius, I. et al. 2013
36839	NG108	UK	2012	302	234	2	Jongerius, I. et al. 2013
36840	NG111	UK	2012	18	237	1	Jongerius, I. et al. 2013
36841	NG112	UK	2012	3	233	5	Jongerius, I. et al. 2013
36842	NG115	UK	2012	159	234	2	Jongerius, I. et al. 2013
36843	NG116	UK	2012	159	234	2	Jongerius, I. et al. 2013
31562	WHO_F	Canada	1990	316	246	7	Unemo et al. 2024
31758	WHO_G	Australia	2007	54	238	1	Unemo et al. 2016
31695	WHO_K	Japan	2003	8	238	1	Unemo et al. 2024
31664	WHO_L	Australia	1996	312	234	2	Unemo et al. 2024
31931	WHO_M	Philippines	1992	3	234	2	Unemo et al. 2024
31764	WHO_N	Australia	2001	54	292	2	Unemo et al. 2016
31461	WHO_O	Canada	1991	37	234	2	Unemo et al. 2024
31566	WHO_P	USA	1999	3	234	2	Unemo et al. 2024
36238	51212	Kenya	2011	364	280	3	Cehovin et al. 2018
36241	T006	Kenya	2013	365	238	1	Cehovin et al. 2018
36248	43305	Kenya	2010	364	234	2	Cehovin et al. 2018
36250	58767	Kenya	2012	21	238	1	Cehovin et al. 2018
36251	63179	Kenya	2013	367	235	4	Cehovin et al. 2018
36261	48871	Kenya	2011	364	234	2	Cehovin et al. 2018
36274	63518	Kenya	2013	366	288	3	Cehovin et al. 2018
36280	47956	Kenya	2011	364	234	2	Cehovin et al. 2018
36297	52492	Kenya	2012	34	234	2	Cehovin et al. 2018

39081	64204	Kenya	2014	366	288	3	Cehovin et al. 2018
39082	64500	Kenya	2014	34	234	2	Cehovin et al. 2018
39083	64579	Kenya	2014	366	288	3	Cehovin et al. 2018
39085	64680	Kenya	2014	364	238	1	Cehovin et al. 2018
39089	64203	Kenya	2014	366	288	3	Cehovin et al. 2018
39092	65320	Kenya	2014	366	288	3	Cehovin et al. 2018
39095	65737	Kenya	2014	366	288	3	Cehovin et al. 2018
39096	56474	Kenya	2012	34	234	2	Cehovin et al. 2018
39097	56327	Kenya	2012	21	234	2	Cehovin et al. 2018
39100	57568	Kenya	2012	34	234	2	Cehovin et al. 2018
39112	44271	Kenya	2010	34	234	2	Cehovin et al. 2018
39114	44254	Kenya	2010	34	234	2	Cehovin et al. 2018
39119	47547	Kenya	2011	375	238	1	Cehovin et al. 2018
39120	47548	Kenya	2011	364	234	2	Cehovin et al. 2018
39124	48563	Kenya	2011	365	238	1	Cehovin et al. 2018
39125	48848	Kenya	2011	365	238	1	Cehovin et al. 2018
39126	48213	Kenya	2011	364	234	2	Cehovin et al. 2018
39127	48502	Kenya	2011	365	238	1	Cehovin et al. 2018

Supplementary Table 8. Primers used in this study

Primer name	Sequence (restriction site underlined)
1632_Upstream_fwd	agtgaattcgagctcggtacccgggGGATCCCATGCATCAAAAG
1632_Upstream_rev	atcgatgatggttAACGCTGGCAAAATAGCC
Kan_fwd	ttttgccagcgttAACCATCATCGATGAATTGTG
Kan_rev	catcgcggttggcCGATGCATGCCAACAGATAAAAC
1632_Downstream_fwd	ttggcatgcatcgGCCAACCGCGATGCGGCA
1632_Downstream_rev	aagcttgcatgcctgcaggtcgactCCTAGGCGGGGCAACGCC
1632_Seq_fwd	ATGAATACTACATTGAAAACTACCTTGACCTCTG
1632_Seq_rev	TTATTTGCCGGTTTGGGTATCCC
Fwd1632	AGCCTTTGCATTGCATATGTGCACCATGATT
Rev1632	TTGTTTGCCCGGATCCTTATTTGCCGGTTT
0651_Upstream_fwd	GCAAACGCAAAACGCCTTGC
0651_Kan_Upstream_rev	caaatggttcgctggttcagacggcGGCGTTAATTTTAAAACGTTTGGCTTTCA
0651_Kan_Downstream_fwd	gttttagtacctagatttagatgacCGTAAATGGCAGTATTACAAAAGGAAAACAATTG
0651_Downstream_rev	GCCATCGGTTTGTTCTATCCCTATATCG
0651_Seq_fwd	GCCTTTGCCGCAAACTGCAC
0651_Seq_rev	GAAATGGTGCGTGATTTCCAATGC
